# Latent Dirichlet Allocation modeling of environmental microbiomes

**DOI:** 10.1371/journal.pcbi.1011075

**Published:** 2023-06-08

**Authors:** Anastasiia Kim, Sanna Sevanto, Eric R. Moore, Nicholas Lubbers

**Affiliations:** 1 Computer, Computational, and Statistical Sciences Division, Los Alamos National Laboratory, Los Alamos, New Mexico, United States of America; 2 Earth and Environmental Sciences Division, Los Alamos National Laboratory, Los Alamos, New Mexico, United States of America; 3 Bioscience Division, Los Alamos National Laboratory, Los Alamos, New Mexico, United States of America; European Molecular Biology Laboratory, GERMANY

## Abstract

Interactions between stressed organisms and their microbiome environments may provide new routes for understanding and controlling biological systems. However, microbiomes are a form of high-dimensional data, with thousands of taxa present in any given sample, which makes untangling the interaction between an organism and its microbial environment a challenge. Here we apply Latent Dirichlet Allocation (LDA), a technique for language modeling, which decomposes the microbial communities into a set of topics (non-mutually-exclusive sub-communities) that compactly represent the distribution of full communities. LDA provides a lens into the microbiome at broad and fine-grained taxonomic levels, which we show on two datasets. In the first dataset, from the literature, we show how LDA topics succinctly recapitulate many results from a previous study on diseased coral species. We then apply LDA to a new dataset of maize soil microbiomes under drought, and find a large number of significant associations between the microbiome topics and plant traits as well as associations between the microbiome and the experimental factors, e.g. watering level. This yields new information on the plant-microbial interactions in maize and shows that LDA technique is useful for studying the coupling between microbiomes and stressed organisms.

## Author summary

Host-microbe interaction may be an important factor determining the performance and survival of an organism under stress. Understanding how microbiomes influence organisms under stress is a challenging new area of research because microbiomes are complex with the potential for complex responses and adaptations to stress that influence their interactions with the other stressed organisms. We show the use of LDA, a data-science technique in the context of environmental microbial datasets to break down the thousands of microbes present in samples into groups called topics; each topic is a group of organisms that occur together as a common pattern in the dataset. We show that this technique, combined with correlation analyses, provides a way to view a very large set of microbes in a sample as a smaller, more manageable set of communities of microbial taxa related to experimental conditions and plant traits. In this way, LDA helps to unravel complex interactions between organisms and their microbiome, which could help to better predict the behavior of real-world ecological systems, and perhaps support them through many challenges brought by changing climate and environment.

This is a *PLOS Computational Biology* Methods paper.

## 1 Introduction

Many interacting factors, such as microbiome community composition, soil chemistry, plant traits, and plant chemistry, may play a crucial role in plant ability to withstand limited water availability. As part of these systems, the microbiome plays a vital role in plant functioning and development and can potentially improve the performance of many biological systems [1–3].

To understand plant-microbiome interaction and effectively reduce the dimensionality of taxonomic microbial data, we use Latent Dirichlet Allocation (LDA) [4], which is a probabilistic generative model developed for language modeling of a corpus, that is, a set of documents. In LDA, each document is represented by the count of the words present in the document. The key assumption behind LDA is that documents are represented as probabilistic mixtures over latent topics, where each topic is characterized by a distribution of words. This factorization of the data is a method to associate words with each other, and, at the same time, perform dimensionality reduction on the documents. Topic modeling with LDA, widely applied for text mining and image retrieval, has been successfully applied in a few biological studies primarily to identify human gut microbial communities [5–8]. However, fewer topic modeling studies are available on microbiomes of non-human organisms, which are primarily analyzed with differential abundance analysis, clustering, dimensional reduction methods, and tests of statistical significance [9–13].

LDA has an advantage over direct clustering techniques in biological settings, because it does not assume that microbial species belong to only one of a set of mutually exclusive clusters; LDA topic compositions may overlap with each other. In a microbial setting, the assumption of exclusivity may oversimplify results because each species may play a different role in different clusters of other microbial species. Instead, in LDA a species may be associated with any number of topics. LDA also provides advantages over correlation analyses that examine individual species; the topic decomposition of a sample provides an interpretable dimensionality reduction, reducing the amount of variables to be analyzed from thousands to a far small number (in our case, between 6 and 25 appeared optimal) by deciding what groups of distributions of components most succinctly explain the data. LDA may reveal topics that are responsible for different ecological functions in plant-microbiome interaction.

LDA can be used not only with 16s rRNA gene amplicon sequencing data but with other types of -omics data, including metagenomics, transcriptomics, proteomics and metabolomics, to find associations between genes/transcripts/metabolites and other metadata. LDA has been previously used to classify RNAseq data and link gene expression profiles to healthy or cancerous tissues [14]. LDA has also been used on a complex multi-omics dataset to identify probable gut microbiota genes, proteins, and metabolites associated with autism [15].

Amplicon sequencing data typically requires some form of normalization due to uneven sequence read depths across samples. Approaches commonly used to address this challenge vary and are widely debated. In summary, normalizing by proportions or using rarefied data are regarded to perform best for community-level comparisons, but face difficulties with spare/rare taxa and data loss; while approaches that transform data using models, such as DESeq2, may perform better for differential abundance analyses, but may result in a higher false-positive rate, overemphasize importance of rare taxa, and are less accurate for community-level analyses [16–18]. While normalization can influence the results obtained from some methods, LDA is not affected by this. LDA handles the count data in a probabilistic way by computing conditional probability of a word given the topic and conditional probability of the topic given a document.

In this work we analyze two datasets of environmental microbiomes of organisms under stress. It is essential to consider the impact of environmental stress factors for a proper management of the plant-microbiome interactions. In the first analysis, to demonstrate the power of LDA and its ability to reproduce analysis results obtained by other means, we performed LDA on a dataset from the literature of coral microbiomes [19] from an experiment to probe disease susceptibility. This study was chosen on the basis of its sample size (95 samples) and because the authors identified specific taxa linked to various traits of interest; it allows us to assess the credibility of LDA analysis for a small dataset of non-human microbiomes. We show that LDA readily reveals the same associations of specific microbial species with experimental conditions as found in [19]. Furthermore, LDA identifies other groups of microbial communities, suggesting further studies to probe their links to biological function (e.g., coral disease susceptibility).

In the second analysis, we conduct LDA on maize microbiomes under drought. It is known that microbial community composition is significantly impacted by drought. Several recent studies on cotton, grass, rice, and peanut root microbiomes have revealed certain phyla that are enriched in water-limited soil [12, 20–22]. Bacterial responses to drought are generally well conserved at the phylum level [23]. Across different soils and plant systems, if phylum has a certain response to drought, this remains generally consistent at lower taxonomic levels due to the broad characteristics, such as Gram +/- classification [23] that promote drought tolerance; however, lineage specific adaptations that are not unique to certain phyla, including trophic strategy, resting stages, or osmolyte production [21, 24], and environmental context [25] explain differing trends between broad and specific taxonomic scales across different soils. The variability in trends at different taxonomic levels highlights the challenges of analyzing the large set of taxa found in microbiome samples. This motivates the use of data-science techniques to view the data en masse.

The dataset of maize microbiomes (119 samples) contains more than 3946 unique bacterial taxa (ASVs) from 27 phyla, 63 classes, 152 orders, and 247 families. We find many significant links between LDA topics, experimental conditions, and plant functional traits. We then identify which taxa contribute most to the topics that can be associated with treatment conditions and plant traits at multiple taxonomic levels.

## 2 Methods

### 2.1 Coral dataset and experiment

The experimental design and detailed results of the coral dataset we performed LDA on can be found in [19]. The following few sentences summarize the key experimental features and study goals by Rosales et al. [19], who identified bacterial taxa linked to resistance and susceptibility to White Band Disease (WBD) in two species of *Acropora* coral. In their experiment, diseased tissue was grafted to healthy *Acropora cervicornis* and *Acropora palmata* to induce a diseased state. Bacterial communities were sampled prior to grafting (control) and seven days after inoculation (treated). Seven days after inoculation, corals were also examined for visual signs of disease lesions and classified based upon their disease status (diseased or visually unaffected) and severity (low, mid, or high disease susceptibility). Microbial community composition at the ASV level was then compared across treatment and outcome groups using “traditional” community-based methods, including ordinations, differential abundance of taxa, and core microbiome analysis, to identify taxa that 1) may cause WBD, 2) are associated with microbiome changes during disease, and 3) potentially increase *Acropora* resistance to WBD. Rosales et al. [19] used pairwise comparisons with a Kruskal-Wallis test to assess the significance of Shannon diversity and evenness in host species. ANOVA and PERMANOVA were used to test the significance of dispersion of the samples and to find significant interactions between groups, respectively. The authors also present beta-diversity (PCA with Euclidean distance were used for visualization) and microbial differential abundance plots to identify the difference between treatments.

### 2.2 Maize dataset and experiment

The maize dataset for this work comes from a serial microbiome propagation studying on how microbiome composition in soil can affect performance of maize and which plant traits are most affected by the microbiome. In this experiment we were interested in understanding the behavior of several plant traits such as stomatal closure point, water use efficiency, maximum rate of photosynthesis, rate of stomatal conductance, drought time (time to permanent stomatal closure from the beginning of the drought treatment), percentage of leaf water content, leaf mass per area, stem diameter, stem height, and root biomass to see if microbiomes that support these traits will improve plant performance under drought and help to find stable microbiomes that help plants to withstand water-limited conditions. While all plant traits are relevant for assessing plant health under drought conditions, we are mainly interested in behavior of stomatal closure point and water use efficiency. Stomatal closure point (SCP) is an important plant desiccation tolerance metric that is linked to plant hydraulics and productivity under drought [26–29]. Water use efficiency (WUEi), on the other hand, is a traditional metric of plant productivity and drought resistance, and it measures the carbon gain of a plant per water lost [30]. Based on the known influence of rhizosphere microbiomes on plant nitrogen availability [31], and the connection between improved nitrogen availability and higher water use efficiency [32, 33], it is plausible to expect that WUEi and SCP could be influenced by the rhizosphere microbiome.

**Fig 1:**
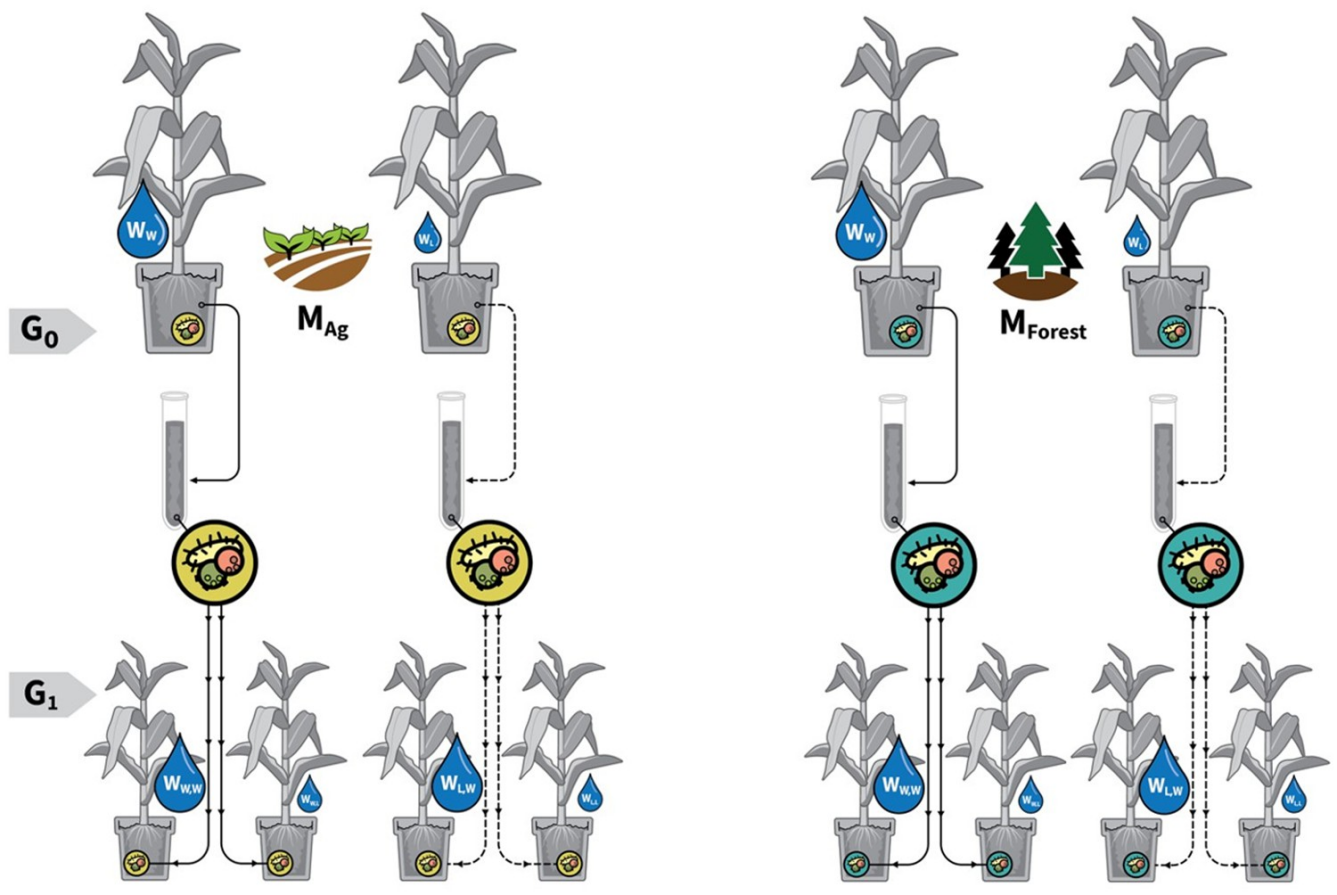
Experimental design for maize. Two generations (G_0_, G_1_) of maize experiment were grown in the greenhouse with microbiomes extracted from agricultural (M_*Ag*_) and forest (M_*Forest*_) soils. Generation 1 plants were grown with microbiomes that were direct descendants of those from generation 0. full-water (*W*_*w*_) and half-water (*L*_*w*_) treatments were imposed to plants during generation 0. The same water treatments were imposed in generation 1 after seed germination, but now so that for half of the plants in each treatment the water treatment was switched.

In this work, we present results from data on 119 plant microbiomes from two generations (called generation 0 and 1) of serial microbiome propagation (Fig 1). These analyses on two generations were performed in order to understand the effect of different water treatments on plant-microbiome interactions with the system. In each generation, 64 maize plants were grown in a greenhouse setting between January and May 2020 in individual 2.6 gal (9.8 L) pots filled with 6L of fritted clay (GreensGrade, 20–50 mesh size: Profile (Buffalo Grove, IL, USA)). The plant seed for each pot in each generation was randomly drawn from a stock of an experimental strain of maize, USDA seed bank inbred line “B73”. For generation 0, 48 of the seeds were inoculated using methods described by [34] by microbial communities originating from one of two natural soils: one collected from an agricultural field near Akron, CO, USA and the other collected from a ponderosa pine forest near Los Alamos, NM, USA, and 16 seeds were planted without inoculation to form a control treatment. After seed germination, the plants were divided into two water treatments: full-water (up to 65% volumetric water content 3 times a week) and half-water (up to 45% volumetric water content 3 times a week). After the plants had grown to a stage showing 9 fully grown leaves (∼ 8 weeks), the watering was stopped and the plants were allowed to dry under complete water withdrawal until stomatal closure occurred (terminal drought). Before the terminal drought, the soil in each pot was sampled for microbiome and soil chemistry analyses, plants were sampled for leaf chemistry analysis, and measured for height, stem diameter, maximum photosynthesis rate, and stomatal conductance (infrared gas analyser; Licor 6400, Licor Inc. (Lincoln, NE, USA)) from which water use efficiency was calculated by dividing the maximum photosynthesis rate by the measured stomatal conductance. During terminal drought, stomatal conductance of each plant was measured daily. When stomatal conductance reached zero, a leaf was cut for leaf water potential measurements (pressure chamber method, PMS Instruments (Al bany, OR, USA)) to determine the stomatal closure point (leaf water potential at which the plant closes stomata), and the time since the beginning of the terminal drought was recorded (drought time). For generation 1, new pots with fritted clay were set up, and new seeds were inoculated by serially transferring the microbial communities from the generation 0 pots with no selection with similar microbiome extraction as for generation 0. Therefore, plants in generation 1 were growing with the microbiomes that were direct descendants of those from generation 0. Due to the high density of roots in the pot, all of the soil was considered “rhizosphere”, thus we chose to use soil cores to sample the rhizosphere community in the pots, rather than performing a separate analysis on soil directly adhered to roots, and because they were a more appropriate reflection of the communities we transferred to the next generation. The same water treatments as in generation 0 were imposed after seed germination, but now so that for half of the plants in each treatment the water treatment was switched. This created two additional treatment categories (stable watering vs. switched watering) for microbiomes from each original soil source and the non-inoculated controls. Similarly to generation 0, generation 1 plants were grown to a stage showing 9 fully grown leaves (∼ 8 weeks), and the plant performance measurements and the terminal drought treatment were conducted as for generation 0. For more detailed information on the experimental setup, plant growing conditions, and plant trait measurements see S1 Text.

Of our original 128 microbiome samples, 9 samples were omitted from the final analysis due to insufficient DNA, sequencing failures, or low read quality and quantity, resulting in the analysis of communities from only 119 plants.

In this paper, we analyze microbiome composition with a focus on the links between microbiomes and watering conditions, initial microbiome soil source, and generation of the experiment.

### 2.3 DNA extraction and microbiome sequencing

Microbial DNA was extracted from homogenized soil core samples using the Qiagen DNeasy PowerSoil kit, with modifications to improve yield from clay soils [35]. 16s rRNA gene amplicon sequencing (V4 region) was used to profile bacterial communities. DNA amplicons were generated using 515F-R806 previously described primers [34] and then sequenced using an Illumina MiSeq (300bp, paired end reads). Raw sequencing reads were demultiplexed using USEARCH11 [36] and dada2 was used to perform quality filtering, primer removal, and read denoising [37]. Default settings were used for dada2 according to the dada2 tutorial (v1.8), except the filterAndTrim function used the following settings to remove primers: truncLen = c(240, 200), truncQ = 2, trimLeft = c(25, 26), maxEE = c(2, 4); only paired reads with a minimum overlap of 100bp were merged, and merged sequences between 250 and 260bp were used to generate the sequence abundance table. Taxonomic classifications were assigned to each unique sequence at the 80% confidence level using the *SILVA* v138.1 database [38, 39]. Unprocessed sequence data have been deposited to the NCBI Sequence Read Archive under the projects *PRJNA*780613 (generation 0 samples) and *PRJNA* 780954 (generation 1 samples).

### 2.4 Topic modeling

LDA, commonly used in text analysis, is fully described in [4]. Sankaran et al. [5] developed some guidelines of the application of probabilistic latent variable models including LDA to human microbiome data. Before we start to discuss our LDA results, let us introduce the analogy between text and soil microbiome analysis we used: the pot samples, bacterial species (taxa), microbial communities are viewed as the documents, words, and topics, respectively. Therefore, the bacterial abundance counts matrix is viewed as the document-term matrix. That is to say that individual pot microbiomes (documents) are broken down into a distribution of topics, and these topics are distributions of taxa (words). A topic is described as a microbial community that may share similar biological functions.

**Fig 2:**
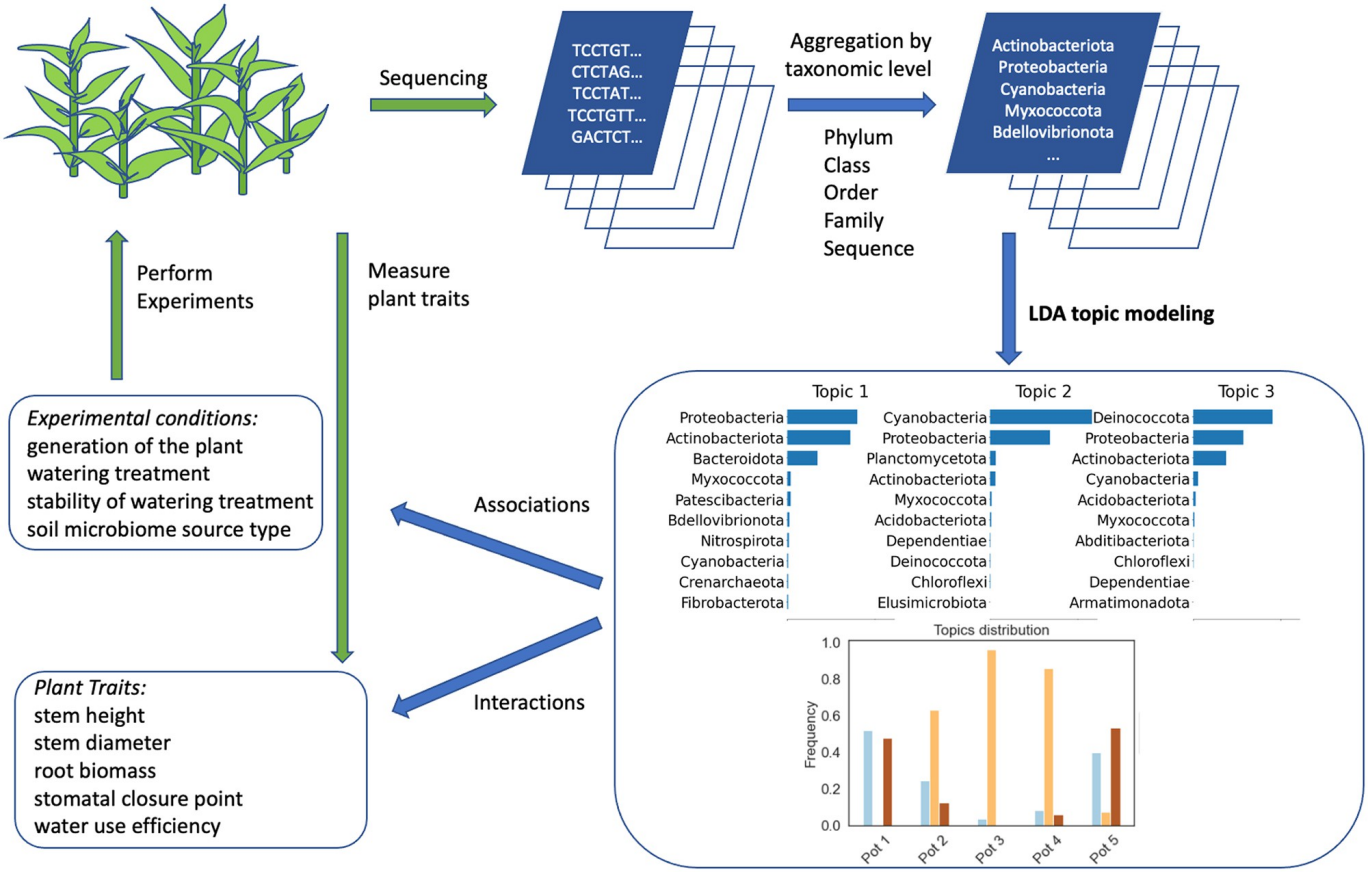
Flowchart of the study design. Schematic LDA topic modeling for *K* = 3 topics.

Fig 2 shows a flow chart of LDA topic modeling at phylum level for *K* = 3 topics. After soil microbiomes were sequenced, microbiome composition was aggregated to a certain taxonomic level, in this case to the phylum level. The desired number of topics *K* (we chose the optimal number as described in Results section) was specified in advance and the bacterial counts matrix was used as an input for the LDA algorithm. LDA infers taxa distribution for each topic and topic distribution for each sample. In this analysis, we used the LDA method implemented in the MALLET software [40] to identify topics at different taxonomic levels. To learn the model, we used Gibbs sampling [41]. We ran LDA for 10,000 iterations to allow log-likelihood per word to stabilize.

### 2.5 Data analysis

Our data is high-dimensional and sparse with a large amount of ASV sequences for which no complete taxonomic classification can be assigned. Due to the differences between text data and microbiome data, some pre-processing approaches and evaluation metrics, such as the removal of common words (stop words) which are assumed not to be useful in describing the document, used for text data were not appropriate in our case. We pre-processed the data by aggregating ASVs to identified taxonomic descriptions associated with the ASV (phylum, class, family, order, and ASV (no aggregation here)), and handled incomplete taxonomic specification for some ASVs by aggregating to the highest known taxonomic level.

Many bacterial taxa present in soils remain unidentified at some taxonomic levels. Despite advances in sequencing technology that allow deep profiling of the taxonomic composition and functional potential of soil microbial communities, databases containing information about environmental microbes remain sparse. Nevertheless, unidentified taxa, also known as “microbial dark matter” are sometimes in high abundance, and can be essential to microbial community interaction networks where they are often found to be major hubs or keystone taxa in these networks, suggesting that they provide important functions and stability to the community [42, 43]. We elected to include unidentified taxa in our analyses, and aggregated these by the most specific known taxonomic classification. Analysis at the ASV taxonomic level (using ASVs directly) avoids the issue of incomplete taxonomic classification. Using this approach, we could perform the analysis on the complete community, rather than only on the classified subset, which may have changed community network structure [43], and thus the outcomes of the LDA analysis.

We omitted the analysis at genus and species levels because more than half of the taxa were unidentified and thus aggregation was harder to perform at these levels. However, we performed the analysis at the ASV (“deepest”) level which allowed us to determine which ASVs (that belong to certain genera/species) were probable in the LDA topics. When analyzing at certain taxonomic level (except at ASV level), we removed taxa with aggregated count of less than 4. We then performed the same analysis described below for each taxonomic level.

The main parameter of LDA that adjusts the level of detail in the resulting model is the number of topics. If one chooses far too many topics, the main drawback is that the factorization becomes less well-conditioned, resulting in redundant topics and an ambiguous document-topic matrix. With too few topics, not all patterns in the data are captured. We chose the number of topics carefully based on topic selection metrics and also checked that using a different number of topics does not reduce the number of associations significantly. We ran 5-fold cross-validation, measured several metrics, and calculated averaged results as a function of the number of topics. We repeated all calculations five times to account for the method’s stochastic nature. We examined the metrics of perplexity, pairwise cosine similarity, coherence, and exclusivity [40] to help us decide how many topics to use at each level of taxonomic identification (S1 Fig). The perplexity score was calculated to see how well a model performs on the unseen held-out test data (20%). A lower perplexity score indicates better generalization performance. A pairwise cosine similarity was calculated between taxa in topics to determine how distinct were the distributions of taxa between topics. We used coherence [44] to measure whether the most probable taxa in a topic tended to co-occur in other topics. The exclusivity metric [40, 45] we report, finds the number of most unique taxa for each topic.

After selecting the number of topics, we ran LDA once again on the full dataset. We then discussed the weighting of topic abundances in terms of experimental conditions, such as water treatment, watering stability treatment, soil source microbiome inoculation type, and generation. We also calculated the number of topics that had strong or moderate association with experimental conditions at each taxonomic level. A topic was moderately associated with the experimental condition (e.g., half-water treatment) if average topic abundance in all pots treated under this condition (e.g., half-water treatment) was at least twice as large (67–80% abundance) but less than four times larger than the average topic abundance under alternative condition (e.g., full-water treatment). A topic was strongly associated with the experimental condition if average topic abundance in all samples treated under this condition was at least four times larger (80–100% abundance) than the average topic abundance under alternative condition. If there were two alternative conditions (e.g., three soil source microbiome inoculation types) then the strength of topic abundance was determined by comparing it to topic abundances under both alternative conditions. We present topic abundance weighting plots that show topics related to different treatments. The weighting was determined by how much the average topic abundance under one treatment was smaller than that under the alternative treatment on a 0–1 scale. LDA outputs probability distribution over topics for each document (sample). To determine topic weighting in case of two treatments (e.g., half-water vs. full-water) for each topic *i* and treatment *t* we first calculated $f_{i,t}=\sum_{j=1}^{n_{t}}r_{j}$, where *r*_*j*_ is a contribution of the topic in document *j* and *n*_*t*_ is the number of samples under a certain treatment (e.g., half-water). For each topic *i* we defined $x_{i,t}=\frac{f_{i,t}}{\sum_{j=1}^{K}f_{j,t}}$, where *K* is the number of topics in the model. We then obtained topic weighting *w*_*i*,*t*_ for each treatment t by normalizing *x*_*i*,*t*_ values across two treatments so that the sum of the contributions for all topics for each treatment is one. To obtain topic abundance weighting plots we calculated plot coordinates where treatment *t*_1_ is located at the bottom or left half of the plot (e.g., half-water, generation 0) and treatment *t*_2_ is located at the top or right half of the plot (e.g., full-water, generation 1) as follows. If $w_{i,t_{1}}>w_{i,t_{2}},w_{i,t_{1}}+w_{i,t_{2}}=1$, then topic coordinate on the plot is $\frac{1-2w_{i,t_{1}}}{w_{i,t_{1}}}$, otherwise if $w_{i,t_{1}}<w_{i,t_{2}},w_{i,t_{1}}+w_{i,t_{2}}=1$, then topic coordinate on the plot is $\frac{2w_{i,t_{2}}-1}{w_{i,t_{2}}}$.

Each LDA microbial community topic is characterized by the distribution of taxa. This allowed us to detect which topics were more associated with pots that were exposed to water-limited treatment or pots with inoculated microbiomes from different soil sources, etc. To determine which taxa abundances were most amplified (exclusive) in the topic in comparison to their abundances in the overall dataset, we defined the term *relative amplification* (also known as *lift* in text analysis [46]) of each taxon within each topic. It is defined as the probability of the taxon given the topic divided by the frequency of that taxon in the overall dataset, and then we normalized each topic across all taxa so that the sum of the relative amplifications for all taxa in each topic is one. In other words, the relative amplification shows which taxon’s abundance is most amplified in the topic in comparison to its abundance in the overall dataset. We also calculated effective number of words for each topic [40], which is computed for each topic as the inverse of the sum of the squared probability of each word in the topic. This metric counts how many taxa are important contributions to each topic, that is the number of taxa that the topic is effectively spread across.

We tested if topics had statistically significant relationships with plant traits. To do this, we calculated a Spearman’s rank correlation coefficient between each topic and each continuous plant trait. Tests were performed at 5% statistical significance level with Holm–Bonferroni correction, which is uniformly more powerful than the Bonferroni correction, to control the family-wise error rate.

To compare the LDA approach with traditional microbiome analysis, we ran Spearman’s rank correlation tests and Indicator Species analysis [47–49] to find significant relationships between individual taxa and plant traits. Indicator Species analysis (using the *multipatt* function in the *IndicSpecies* v1.7.9 *R* package) did not identify any taxa significantly associated with any treatment following multiple test correction. Differential abundance analysis was conducted using *Corncob* *R* package [50]. It identified numerous taxa that significantly differed in abundance between treatments. *Corncob* uses a beta-binomial model to compare taxa abundances using un-normalized sequencing data.

To test the robustness of our LDA results that might vary due to the randomness of LDA, we compared results from three different LDA runs at the phylum level. Cosine similarity between taxa in topics obtained from the different runs was calculated to determine the consistency of the topics from run to run. S5 Table shows the consistency of the topics between three different runs. Although topics related to the watering treatments have lowest cosine similarity between runs, the most probable taxa in these topics were the same.

We also fitted different number of topics at the phylum level to see if this significantly affects the results (S6 Table). Even if using more topics can result in more associations between topics and treatments, cosine similarity between topics within a model can be used to avoid overfitting.

## 3 Results

### 3.1 LDA results on coral dataset

To assess the performance of LDA-based microbiome analysis prior to analyzing our own maize dataset, we performed LDA analysis on a previously published dataset [19] that used “traditional” microbiome analysis methods.

**Fig 3:**
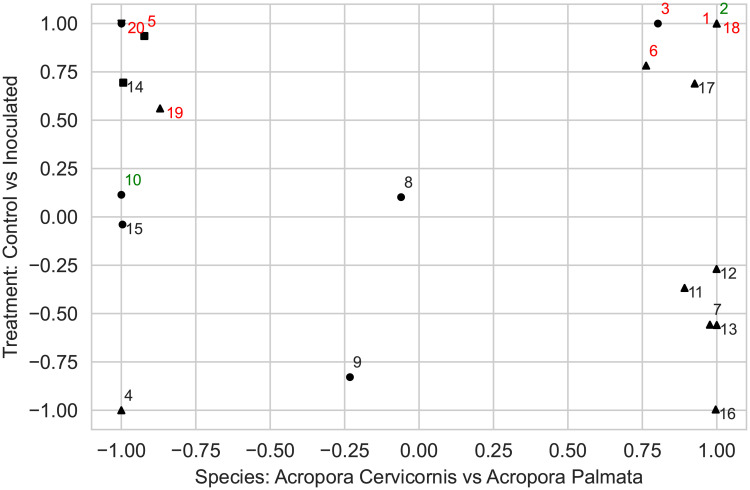
Topic abundance weighting for the treatment and species types. All topics to the left of topic 9 are associated with *Acropora cervicornis* species. All topics to the right of topic 8 are associated with *Acropora palmata* species. All topics below topic 11 are associated with Control treatment whereas all topics above topic 15 are associated with Inoculated (diseased or visually unaffected) treatment. Topics in color are topics associated with an experimental outcome. Topic 20 is associated with Low disease susceptibility; topics 5 and 14 are associated with Medium disease susceptibility; topics marked with triangle up symbols are associated with High disease susceptibility.

**Fig 4:**
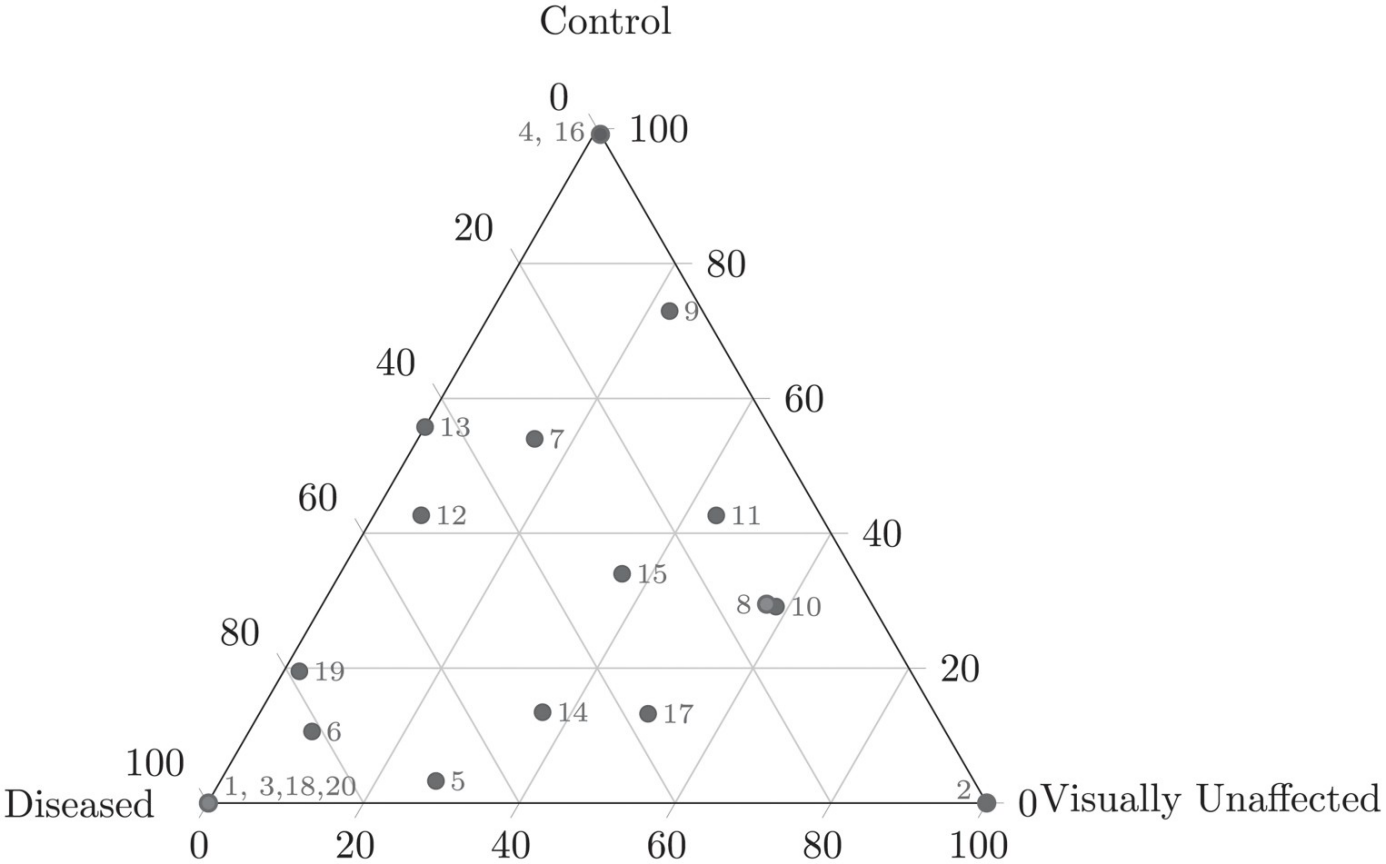
Ternary plot of topic abundances for the outcome type (control, diseased, visually unaffected).

**Fig 5:**
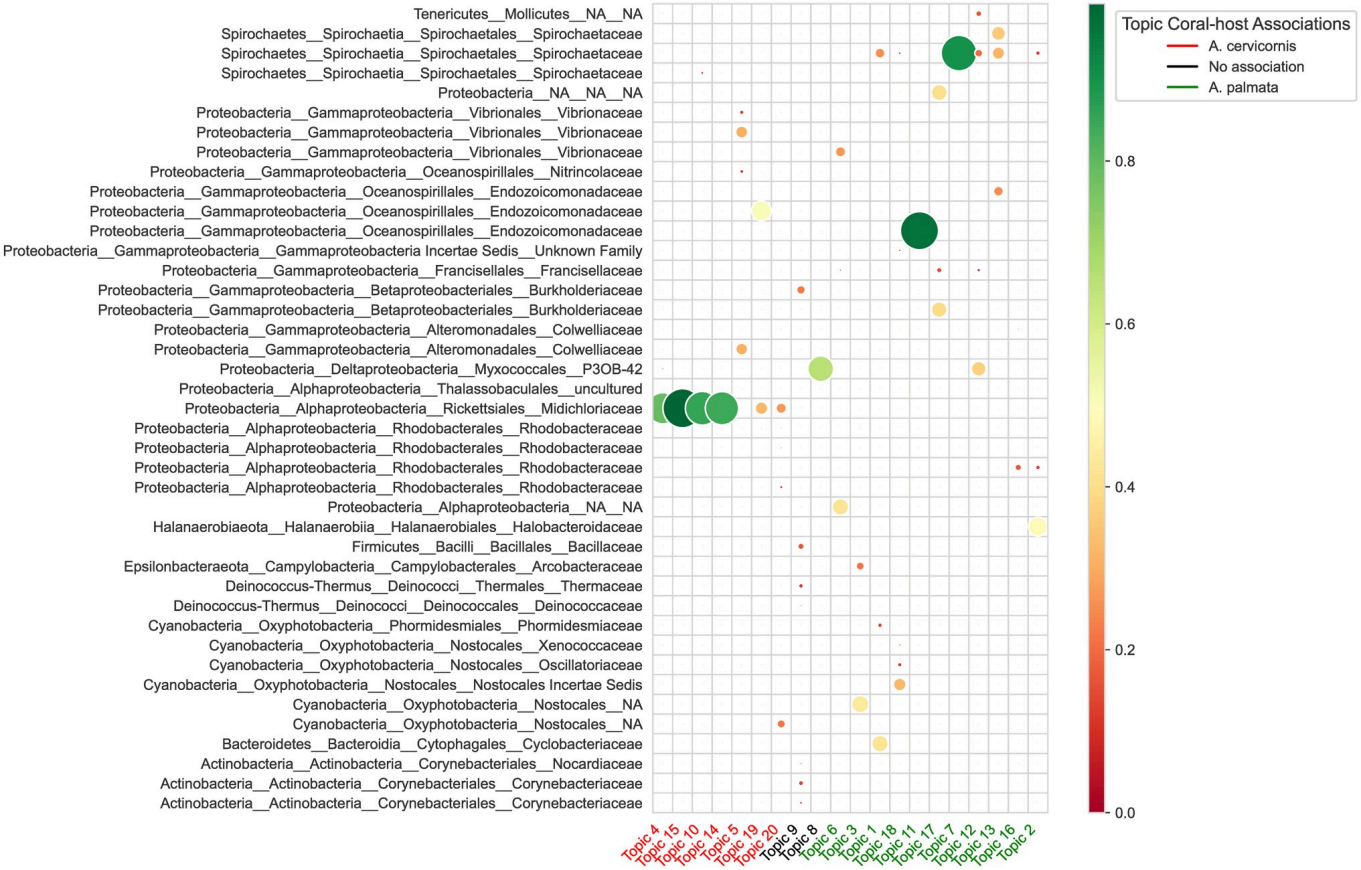
Distribution of ASV sequences in each learned LDA topic. Topics to the left of topic 9 are strongly associated with *Acropora cervicornis* species, and topics to the right of topic 8 are strongly associated with *Acropora palmata* species. The labels are written in the *phylum*_*class*_*order*_*family* format, the names of genera, species, and ASVs are not shown. Only probabilities (color circles) greater than 0.1 are shown. Smaller circles are displayed on the top of larger circles.

By running LDA with 20 topics, we found 7 and 11 topics that were strongly associated with *Acropora cervicornis* and *Acropora palmata* species, respectively. Most of the topics were linked to some experimental conditions such as treatment (control and inoculated), outcome (control, visually unaffected, and diseased), and disease susceptibility (low, mid, and high) (Figs 3 and 4). We found that *Midichloriaceae* was the dominant family in topics relevant to *A. cervicornis* species, and the families *Spirochaetaceae* and *Endozoicomonadaceae* were found to be dominant in topics abundant in samples from *A. palmata* species (Fig 5).

Two topics 4 and 16, each representing different *Acropora* species, were connected to control samples. Topic 4, linked to *Acropora cervicornis*, was dominated by the presence of ASVs from the families *Midichloriaceae, P3OB-42, Endozoicomonadaceae, Nannocystaceae*, and several ASVs from to the order *Phormidesmiales*. On the other hand, topic 16, representing control samples from *A. Palmata* species, was dominated by the presence of ASVs from *Rhodobacteraceae, Colwelliaceae*, and *Phormidesmiaceae* families.

We found three topics related to visually unaffected samples, ASVs from the families *Midichloriaceae, Spirochaetaceae, Francisellaceae, Kiloniellaceae*, and *Endozoicomonadaceae* in *A. cervicornis* were dominant in topic 10, and ASVs from *Halobacteroidaceae, Rhodobacteraceae, Spirochaetaceae, Desulfobacteraceae, Clostridiaceae, Lachnospiraceae*, and *Vibrionaceae* families were more probable in topic 2 related to visually unaffected *A. palmata* species. Topic 20 was the only topic associated with *A. cervicornis* species and low disease susceptibility. It was mainly dominated by the ASVs from *Midichloriaceae* and *Rhodobacteraceae* families and ASVs from the orders *Nostocales* and *Alteromonadales*. These results suggest that these taxa may promote resistance to disease in *Acropora* species. On the contrary, topic 19, associated with *A. cervicornis* species and high disease susceptibility, was dominated by the presence of ASVs mainly from *Endozoicomonadaceae, Midichloriaceae*, and *P3OB-42* families. Several topics were relevant to *A. palmata* species and high disease susceptibility and were dominated by the presence of ASVs from *Fulvivirga, Arcobacter, Oscillatoria* genera, *Nostocales* order, and *Alphaproteobacteria* class.

The LDA results with the most probable taxa in each topic associated with one of two species, certain treatment or outcomes mostly agreed (Table 1) with the findings in [19]. LDA results agreed with the authors findings of the dominant families related to both species, control samples in *Acropora cervicornis* species, and low and high disease susceptibilities in *Acropora cervicornis* species. Rosales et al. [19] found a core bacteria member in both species from the order *Myxococcales (P3OB-42)* at relatively higher abundances in corals with lower rates of disease development following grafting. Though *P3OB-42* was not found among most probable families in topics 2 and 10, it was the most probable family in topic 8 which was related to visually unaffected samples without being ascribed to either species. A single ASV, designated *Sphingobium yanoikuyae* (family *Sphingomonadacea*), was significantly detected in both species of disease-exposed and visually unaffected samples [19]. LDA results in agreement with this finding, *Sphingobium yanoikuyae* was found in topics 17, 19 (diseased) and 8 (visually unaffected). Rosales et al. [19] found *Ralstonia* genus at higher relative abundances in negative control samples. This genus was the most dominant taxon (22.4%) in LDA topic 9 associated with only control samples and nothing else.

There were some taxa-treatment associations identified by Rosales et al. [19] that LDA failed to identify. In particular, LDA did not detect *Spirochaetaceae* and *Endozoicomonadacea* as most dominant families in control samples of *A. Palmata* species. Rosales et al. [19] found relatively higher abundances of the genus *Vibrio* in visually unaffected and diseased corals compared to control corals in *A. cervicornis*. LDA topic associated with visually unaffected corals did not show higher abundances of this genus compared to that in the topic associated with control samples. However, genus *Vibrio* was the most dominant taxon in topic 5 associated with medium disease susceptibility. For outcome in *A. palmata*, the families *Rhodobacteraceae* (genus *HIMB11*) and *Cryomorphaceae* (uncultured genus) were significantly abundant and highly associated with visually unaffected and diseased corals [19]. LDA found higher associations of these genera in topic 6 associated with high disease susceptibility. Therefore, the biggest disagreement between LDA results and relative/differential abundances analyses results from [19] was related to diseased samples in both coral species and to control samples in *A. palmata* species (Table 1). It should be noted that the standard deviations in relative abundance analysis were very high which made the comparison of average relative abundances in samples with LDA probabilities harder.

Though LDA did not detect all associations present in [19], LDA results revealed additional taxa that were linked to certain treatments, outcomes and disease susceptibility that were not previously discussed. Specifically, Rosales et al. [19] did not find any strong associations of taxa and high disease susceptibility in *A. palmata* species samples, while LDA did.

This suggests that LDA was capable not only to detect most associations as were found by the researchers in the previous study but also complement the results with taxa that were less abundant in data but still may interact with other taxa towards the White Band disease resistance.

**Table 1:** Comparison of LDA results and results obtained from the relative abundance analysis (Tables 2 and 3 in [19]). Table shows mean and standard deviation (SD) relative abundances (RA) in % by taxon of core microbiomes per experimental outcome and coral-host [19] as wells as probability % of these taxa in LDA topics associated with corresponding outcome and coral species. Multiple LDA topics were associated with Diseased outcome in both coral species. Topic 20 and topic 5 were associated with low and medium disease susceptibility, respectively, whereas topics 19, 1, 6, and 18 were associated with high disease susceptibility.

Coral species	Outcome	Taxon	Mean/SD RA	LDA probabilities
*Acropora cervicornis*	Control Topic 4	*Midichloriaceae*	93.2/18.6%	79.5%
		*P3OB-42*	4.6/17.7%	6.2%
		*Endozoicomonadaceae*	2.2/2.6%	2.1%
	Visually unaffected Topic 10	*Midichloriaceae*	83.6/33.1%	86.6%
		*P3OB-42*	14.2/32.9%	0.15%
		*Endozoicomonadaceae*	2.2/2.9%	0.6%
	Diseased Topics 20/5/19	*Midichloriaceae*	97.4/3.9%	26.5/0.002/32.1%
		*Endozoicomonadaceae*	1.3/3.6%	0.0002/0.07/55.6%
		*P3OB-42*	0.8/1.2%	0.03/0.007/2.8%
*Acropora palmata*	Control Topic 16	*Spirochaetaceae*	65.1/37.4%	0.19%
		*Endozoicomonadaceae*	26/33.4%	0.03%
		*Midichloriaceae*	8.8/23.2%	0%
	Visually unaffected Topic 2	*Spirochaetaceae*	57/51.3%	15%
		*Proteobacteria*	21.4/42.5%	23.3%
		*P3OB-42*	17.6/39%	0.03%
		*Midichloriaceae*	2.8/2.5%	0.001%
		*Cryomorphaceae*	0.76/1.23%	0.04%
	Diseased Topics 3/1/6/18	*Spirochaetaceae*	79.2/29.1%	0.29/25.4/0/8.4%
		*Endozoicomonadaceae*	13.1/18.1%	0.0002/0.01/0.7/0.07%
		*Midichloriaceae*	1.9/2.6%	0/0/0/0.001%
		*Cyanobiaceae*	4.7/11.9%	0.05/1/5.6/0.07%

### 3.2 LDA results from our maize experiment

LDA results provide a window into which taxa might function together as a system and be related to different experimental conditions and plant traits. In this section, we present the analysis results at phylum, class, order, family, and sequence (ASV) taxonomic levels from the microbiome sequential propagation experiment with maize. Based on the topic selection metrics mentioned above, we decided to use *K* = 6, 8, 10, 20, and 25 topics at the phylum, class, order, family, and ASV levels, respectively, as we did not observe significant improvement in metrics behaviors for larger topic counts. At the phylum level we observed that perplexity dropped at *K* = 6, cosine similarity was the same for *K* < 7 (S1(a) Fig), and coherence was not changing much for *K* = 5, 6, 7 (S1(b) Fig). Therefore, we decided to use *K* = 6 topics at the phylum level. Detailed plots and tables for each taxonomic level are available in S2–S14 Figs and S1–S19 Tables, including explicit lists of all topic compositions and their associations with treatment conditions and interactions with plant traits. We present summary tables (Tables 2, 3, 4 and 5) that capture the main results of the analyses at all taxonomic levels.

**Table 2:** Number of topics associated with experimental conditions at each taxonomic level. Strong (topic abundance is four times (×4) larger than for other treatments) and moderate (topic abundance is twice (×2) as large but less than four times larger than that for other treatments) topic associations are shown for each experimental condition. The numbers in parentheses represent number of topics associated with at least one experimental condition and overall number of topics used in LDA at taxonomic level.

Experimental conditions	Generation				Watering				Stability				Soil source					
	0		1		Half		Full		Stable		Switched		Agricultural		Forest		None	
	×4	×2	×4	×2	×4	×2	×4	×2	×4	×2	×4	×2	×4	×2	×4	×2	×4	×2
Phylum (3/6)		1				1		1										
Class (6/8)		1				2		1						1		1		
Order (9/10)	1	2	1	2	1	2	1	3		2		1	1					1
Family (20/20)	4	2	8	2	3	4	4	3		4		1	3	1	2	1	2	
ASV (24/25)	4	2	15	1	8	3	10		5	1	4	3	8	1	8		4	1

**Table 3:** Number of topics at each taxonomic level that showed statistically significant relationships between topics and plant traits based on a Spearman’s rank correlation coefficient with Holm–Bonferroni correction. The numbers in parentheses represent number of different topics associated with significant relationships and overall number of topics used in LDA at taxonomic level. The last four columns are Leaf mass per area, % Leaf water content, Stomatal conductance, and Water use efficiency.

Taxonomic level	Stem height	Stem diameter	Root biomass	LMA	LWC	Cond	WUEi
Phylum (3/6)	1	3	1	1	0	1	0
Class (3/8)	2	1	1	1	0	0	0
Order (6/10)	4	1	3	2	0	0	0
Family (11/20)	5	3	5	1	3	0	0
ASV (15/25)	13	5	8	0	0	0	1

**Table 4:** Most probable and amplified taxa in some topics associated with experimental conditions at phylum, class, and order taxonomic levels. Left: average percentages of most abundant taxa in pots for each taxonomic level. Middle: topic association with experimental conditions (HW: half-water, FW: full-water, G0: generation 0, G1: generation 1, AG: agricultural soil source). Right: most probable taxa in topic with probabilities are displayed in each upper row; most amplified taxa with its value (see Methods) are displayed in each lower row, some taxa were unidentified at lower levels. Note that unidentified taxa were aggregated to the highest known taxonomic level, meaning that they may represent one or several classes, orders, etc. Not all topics are listed.

Most abundant taxa in data		Experimental conditions	Most probable taxa (upper row) followed by the most amplified taxa (lower row)
Phylum	*Proteobacteria 44.15%*, *Bacteroidota 13.67%*, *Actinobacteriota 13.08%*, *Verrucomicrobiota 9.05%*, *Cyanobacteria 8.01%*	HW	*Actinobacteriota 0.71*, *Bacteroidota 0.15*, *Patescibacteria 0.04*.
			*Fibrobacterota 0.16*, *Patescibacteria 0.15*, *Crenarchaeota 0.15*, *Actinobacteriota 0.14*, *Firmicutes 0.12*.
		FW	*Proteobacteria 0.49*, *Verrucomicrobiota 0.34*, *Bacteroidota 0.11*.
			*WPS-2 0.13*, *Verrucomicrobiota 0.13*, *Sumerlaeota 0.12*, *Desulfobacterota 0.12*, *Gemmatimonadota 0.11*.
Class	*Gammaproteobacteria 25.65%*, *Alphaproteobacteria 18.73%*, *Bacteroidia 13.52%*, *Actinobacteria 12.41%*, *Verrucomicrobiae 8.83%*, *Cyanobacteriia 7.21%*	HW	*Actinobacteria 0.62*, *Alphaproteobacteria 0.26*, *Bacteroidia 0.05*.
			*Bacilli 0.09*, *Nitrososphaeria 0.09*, *Proteobacteria_NA 0.09*, *Ktedonobacteria 0.09*, *Actinobacteria 0.09*.
		FW	*Verrucomicrobiae 0.46*, *Alphaproteobacteria 0.20*, *Bacteroidia 0.17*, *Planctomycetes 0.06*
			*WPS-2_NA 0.11*, *Gemmatimonadetes 0.10*, *Verrucomicrobiae 0.09*, *Bacteroidota_NA 0.09*, *Planctomycetes 0.09*
Order	*Burkholderiales 18.96%*, *Rhizobiales 9.68%*, *Micrococcales 8.1%*, *Verrucomicrobiales 6.01%*, *Sphingobacteriales* 5.83%, *Deinococcales 4.95%*, *Sphingomonadales 4.13%*	G0	*Chitinophagales 0.16*, *Burkholderiales 0.16*, *Rhizobiales 0.11*, *Azospirillales 0.11*.
			*Steroidobacterales 0.05*, *Nannocystales 0.05*, *Thermoanaerobaculales 0.05*, *Azospirillales 0.04*, *Isosphaerales 0.04*.
		G1	*c_Cyanobacteriia_NA 0.42*, *Rhizobiales 0.10*, *Burkholderiales 0.08*, *Sphingomonadales 0.06*.
			*c_Parcubacteria_NA 0.08*, *c_Cyanobacteriia_NA 0.08*.
		HW	*Micrococcales 0.36*, *Rhizobiales 0.18*, *Sphingobacteriales 0.11*, *Propionibacteriales 0.06*.
			*Fibrobacterales 0.04*, *c_MB-A2–108_NA 0.03*, *Solibacterales 0.03*, *PeM15 0.03*, *Saccharimonadales 0.03*.
		FW	*Verrucomicrobiales 0.47*, *Burkholderiales 0.19*, *Rhizobiales 0.08*.
			*c_Actinobacteria_NA 0.09*, *Immundisolibacterales 0.09*, *Verrucomicrobiales 0.08*, *p_WPS-2_NA 0.07*.
		FW	*Chloroplast 0.33*, *Burkholderiales 0.14*, *Rhizobiales 0.11*, *Chthoniobacterales 0.09*, *Sphingobacteriales 0.05*.
			*Chloroplast 0.06*, *Bacillales 0.05*, *RBG-13-54-9 0.05*, *Ga0077536 0.04*, *Silvanigrellales 0.04*.
		G0, FW	*Burkholderiales 0.13*, *Cytophagales 0.13*, *Sphingomonadales 0.10*, *Rhodobacterales 0.08*, *Verrucomicrobiales 0.07*.
			*VC2.1_Bac22 0.04*, *Chloroflexales 0.04*, *Spirochaetales 0.03*, *R7C24 0.03*.
		G1, FW	*Xanthomonadales 0.40*, *Diplorickettsiales 0.07*, *Planctomycetales 0.07*, *Rhizobiales 0.06*, *Cytophagales 0.06*.
			*Xanthomonadales 0.08*, *Diplorickettsiales 0.08*, *Puniceispirillales 0.06*, *Coxiellales 0.06*, *Pirellulales 0.05*.
		G1, HW, AG	*Streptomycetales 0.19*, *Burkholderiales 0.16*, *Rhizobiales 0.12*, *Cytophagales 0.08*, *Vampirovibrionales 0.07*.
			*Streptomycetales 0.10*, *Vampirovibrionales 0.90*, *Caedibacterales 0.08*, *Nitrososphaerales 0.08*.

**Table 5:** Most probable and amplified taxa in topics strongly associated with experimental conditions at family and ASV taxonomic levels. Left: average percentages of most abundant taxa in pots for each taxonomic level. Middle: topic association with experimental conditions (HW: half-water, FW: full-water, G0: generation 0, G1: generation 1, AG: agricultural soil source, FR: forest soil source, ST: stable watering, SW: switched watering). Right: most probable taxa in topic with probabilities are displayed in each upper row; most amplified taxa with its value (see Methods) are displayed in each lower row, some taxa were unidentified at lower levels. Note that unidentified taxa were aggregated to the highest known taxonomic level, meaning that they may represent one or several classes, orders, etc. Not all topics are listed.

Most abundant taxa in data		Experimental conditions	Most probable taxa (upper row) followed by the most amplified taxa (lower row)
Family	*Comamonadaceae 7.78%*, *Micrococcaceae 7.21%*, *Oxalobacteraceae 6.20%*, *Deinococcaceae 4.95%*, *Sphingomonadaceae 4.13%*, *Rhizobiaceae 3.96%*, *Verrucomicrobiaceae 3.90%*	HW	*Micrococcaceae 0.51*, *env.OPS_17 0.10*, *Sphingomonadaceae 0.05*.
			*Solibacteraceae 0.07*, *Oligoflexales 0.05*, *Micromonosporaceae 0.05*.
		FW	*Xanthomonadaceae 0.50*, *Comamonadaceae 0.12*, *Verrucomicrobiaceae 0.07*.
			*Xanthomonadaceae 0.14*, *Coxiellaceae 0.08*, *Pirellulaceae 0.07*.
		FW	*Verrucomicrobiaceae 0.44*, *Comamonadaceae 0.10*, *Crocinitomicaceae 0.07*.
			*Verrucomicrobiaceae 0.12*, *Rickettsiaceae 0.08*, *Crocinitomicaceae 0.07*.
		G0	*Chitinophagaceae 0.18*, *Azospirillaceae 0.11*, *Pseudomonadaceae 0.09*, *Micrococcaceae 0.09*, *Rhizobiaceae 0.06*.
			*Nannocystaceae 0.04*, *Alcaligenaceae 0.04*, *Steroidobacteraceae 0.04*.
		G1	*o_Verrucomicrobiales_NA 0.37*, *Oxalobacteraceae 0.10*, *Micrococcaceae 0.08*.
			*o_Verrucomicrobiales_NA 0.12*, *o_Armatimonadales_NA 0.09*, *o_NRL2_NA 0.07*.
		G1, HW, AG	*Streptomycetaceae 0.27*, *Comamonadaceae 0.12*, *Oxalobacteraceae 0.12*, *Microscillaceae 0.06*.
			*Streptomycetaceae 0.11*, *c_Oligoflexia_NA 0.08*, *Nitrososphaeraceae 0.07*.
		G1, HW, FR	*Burkholderiaceae 0.46*, *Micrococcaceae 0.07*, *Sphingomonadaceae 0.04*, *Pseudonocardiaceae 0.03*.
			*Burkholderiaceae 0.10*, *Nakamurellaceae 0.09*, *Kineosporiaceae 0.06*.
ASV	*genus_Pseudarthrobacter 6.75%,class_Cyanobacteriia 4.06%,genus_Deinococcus 3.69%,family(ies)_Verrucomicrobiaceae 2.74%, family_Comamonadaceae 2.72%*	G1, HW, AG, ST	*g_Streptomyces 0.16* *f_Comamonadaceae 0.08*, *g_Pseudarthrobacter 0.06*, *f_Microscillaceae 0.04*.
			*g_Psychroglaciecola 0.02*, *g_Gordonia 0.02*, *g_Opitutus 0.02*.
		G1, FW, AG, ST	*f_Verrucomicrobiaceae 0.07*, *g_SH-PL14 0.05*, *o_Burkholderiales 0.04*.
			*f_Parachlamydiaceae 0.01*, *f_Holosporaceae 0.01*, *g_Gaiella 0.01*.
		G1, HW, FR	*g_Pseudarthrobacter 0.12*, *f_env.OPS_17 0.04*, *g_Methylorubrum 0.04*.
			*o_Cytophagales 0.01*, *g_Opitutus 0.01*, *s_aurantiaca/mikuniensis 0.01*.
		G1, FW, FR, ST	*o_Chloroplast 0.21*, *f_Rubritaleaceae 0.08*, *s_aerilata/phosphatilytica 0.05*, *s_spinosum 0.04*.
			*Rickettsiaceae 0.01*, *Chlamydiales 0.01*, *67–14 0.01*.
		G1, HW, AG, SW	*g_Pseudarthrobacter 0.08*, *f_env.OPS_17 0.06*, *c_Cyanobacteriia 0.06*.
			*f_Azospirillaceae 0.02*, *g_Conexibacter 0.02*, *f_KD3–93 0.02*.
		G1, FW, FR, SW	*f_Verrucomicrobiaceae 0.15*, *f_Comamonadaceae 0.07*, *f_Dyadobacter 0.04*, *g_Pseudarthrobacter 0.04*.
			*g_Peredibacter 0.01*, *g_Noviherbaspirillum 0.01*, *g_Aminobacter 0.01*.

When analyzed at different taxonomic levels, LDA allowed us to identify topics moderately and strongly related to the experimental conditions at each level (Table 2). We found that microbiome topics were more associated with experimental conditions at lower taxonomic levels than at higher taxonomic levels as expected based on high functional diversity that can be present in groups belonging to the same high taxonomic level (Table 2). Topics were associated mostly with water treatment and plant generation, and less with soil microbiome inoculation source type and stability of the watering treatment. We found that at each taxonomic level there was at least one topic that was associated with the full- or half-water treatments, however, strong associations were only observed starting from the order taxonomic level. Strong topics associations with the generation and particular soil microbiome inoculation source type were detected only at order level and lower. We observed significant skewness of topics toward the stability of watering treatment only at the lowest ASV taxonomic level. At the order level and lower, several topics that were simultaneously strongly associated with multiple experimental conditions appeared. For example, one of the topics was strongly associated with half-water treatment and was more prevalent in generation 1 plants with agricultural microbiome inoculation. At both family and ASV taxonomic levels, all topics were moderately or strongly related to at least one experimental condition. We also detected many moderate associations of topics with experimental conditions at each level.

We found statistically significant relationships between learned topics and plant structural traits, including stem diameter, height, and root biomass at each taxonomic level (Table 3). We did not observe any statistically significant relationships between learned topics and functional plant traits (stomatal closure point (SCP) and water use efficiency (WUEi)) except at the ASV taxonomic level where we found a significant relationship of one topic with WUEi. Overall at each taxonomic level, some relationships were significant (p-value (SCP) < 0.001 and p-value (WUEi) < 0.05), but the corrections for multiple testing precluded their significance.

### 3.3 Identifying taxa related to experimental conditions

We present taxa along with their probabilities and relative amplifications in several selected topics strongly and moderately associated with at least one experimental condition in Tables 4 and 5. For each topic we calculated the effective number of taxa, and we highlighted the taxa whose ranks are under this threshold in bold (S1, S7 and S10 Tables), indicating which taxa are the most important contributions to the content of this topic. For phylum-level analysis, this was the top 2–4 taxa for each phylum in the topic. For the ASV level, each topic was spread across several dozen taxa.

According to the previous studies conducted on different host plants across different soils, *Actinobacteriota* and *Firmicutes* tend to increase in response to drought, *Bacteroidota* and *Verrucomicrobiota* decrease, and *Proteobacteria, Planctomycetota*, and *Acidobacteriota* compositions change [21, 25]. LDA results at phylum taxonomic level mostly agreed with these trends. In particular, *Proteobacteria* and *Verrucomicrobiota* were dominant in the topic linked to full-water treatment, whereas *Actinobacteriota* was dominant phyla in the topic related to half-water treatment. *Bacteroidota* was dominant in both half and full-water associated topics. *Firmicutes* phylum was present only in 1% of data, as expected it did not showed up among most probable phyla in any topic. However, *Firmicutes* was the one of the most amplified phyla in the topic related to half-water treatment (Table 4).

Microbial communities detected in class-level topics related to water treatment followed the same trends as at the phylum level. In particular, *Actinobacteria* was the most probable class at half-water associated topics, and *Verrucomicrobiae* was the most dominant class at full-water associated topics. Classes *Alphaproteobacteria* and *Bacteroidia* were dominant in both half- and full-water associated topics. *Bacilli* in *Firmicutes* phylum was the most amplified class in the topic related to half-water treatment.

Starting from order taxonomic level, most topics have become associated with multiple experimental conditions, and therefore it became more difficult to highlight orders that were only associated with one of the conditions. We note that several most amplified orders were unidentified ones. This result highlights an importance of keeping unidentified taxa in the analysis. Orders *Micrococcales* in *Actinobacteria* class, *Sphingobacteriales* in *Bacteroidia* class, and *Rhizobiales* in *Alphaproteobacteria* class formed a microbial community associated with water-limited soil. On the other hand, orders *Burkholderiales* (*Gammaproteobacteria*) and *Rhizobiales* (*Alphaproteobacteria*) were dominant in the topic linked to full-water treatment. Another *Verrucomicrobiae* order, *Verrucomicrobiales*, was the most probable in the topic linked to full-water treatment and to plants without soil microbiome inoculation.

Analysis at family level showed that family *Micrococcaceae* (*Actinobacteria*) together with a few families from *Sphingobacteriales* order formed a community linked to half-water treatment. Topics mainly consisted of families *Verrucomicrobiaceae* (*Verrucomicrobiae*) and *Comamonadaceae* (*Gammaproteobacteria*) were linked to full-water treatment.

**Fig 6:**
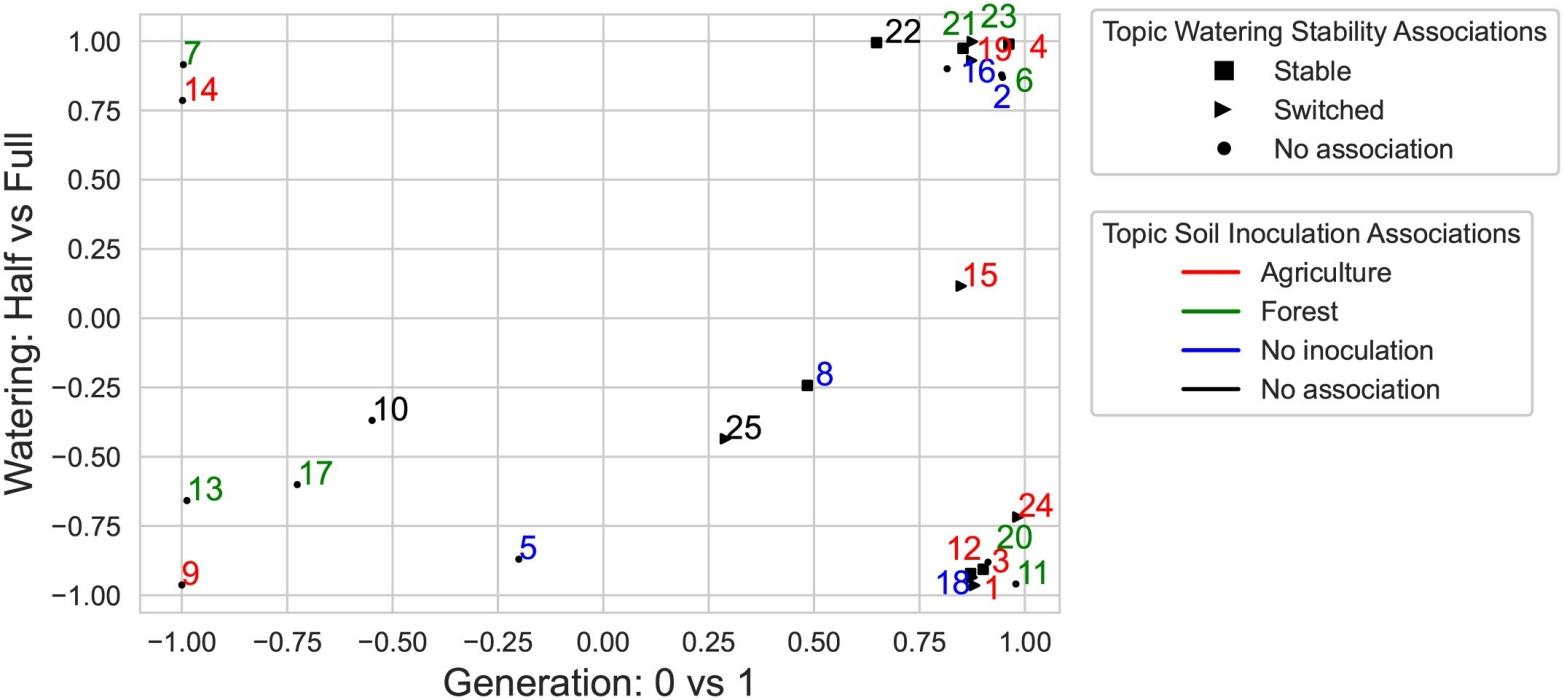
Topic abundance weighting for the water treatment and generation on a 0–1 scale. All topics to the left of topic 5 are associated with generation 0. All topics to the right of topic 25 are associated with generation 1. All topics below topic 25 are associated with half-water treatment whereas all topics above topic 15 are associated with full-water treatment. Topics are colored according to the soil microbiome inoculation source. The shapes of the markers corresponding to the topic association with stability of watering treatment. Note: the weighting was calculated by how much average topic abundance under one treatment was different from that under alternative one (see Methods).

**Fig 7:**
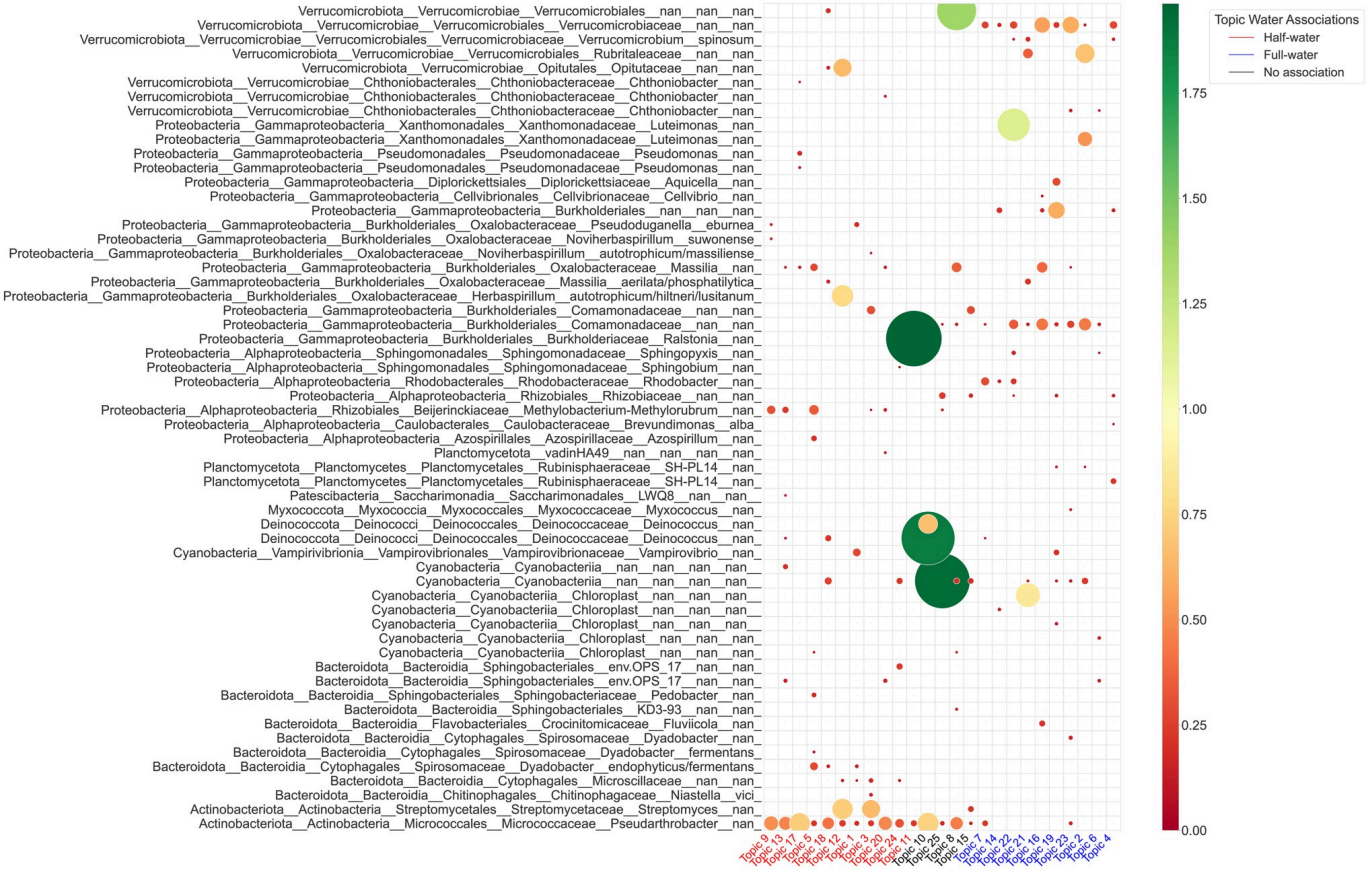
Distribution of ASVs in each learned LDA topic. Topics to the left of topic 10 are associated with half-water treatment, and topics to the right of topic 15 are associated with full-water treatment. The labels are written in the *phylum*_*class*_*order*_*family*_*genus*_*species* format, the names of genera, species, and ASV are not shown. Only probabilities (color circles) greater than 0.025 are shown. The sizes of the circles representing probabilities are multiplied by 4 for visualization purposes. Smaller circles are displayed on the top of larger circles.

All topics at ASV taxonomic level were moderately or strongly related to at least one experimental condition (Fig 6). An ASV from *Pseudarthrobacter* genus (from 5 to 18% in LDA topics) along with an ASV from *Methylobacterium-Methylorubrum* genus (*Alphaproteobacteria* class) (3.5–9%) were found in topics linked to half-water treatment (Fig 7). Several ASVs from *Verrucomicrobiales* order (from 5 to 18% in LDA topics) along with an ASV in *Comamonadaceae* family (*Gammaproteobacteria* class) (3.5–11%) formed a community linked to full-water treatment. The microbial community consisted mainly from three ASVs in *Streptomyces* genus (18.2%), *autotrophicum/hiltneri/lusitanum* species (19.2%), and *Opitutaceae* family (16.2%), respectively, was associated with half-water treatment and generation 1 plants with agriculture soil microbiome inoculation.

The analyses on different taxonomic levels gave insights into the homogeneity of the function of the lower level microbial communities that compose higher level taxonomic communities. Certain taxa were highly probable in topics associated with the same treatments at each taxonomic level. This happened either because a certain ASV was highly abundant in the data and was linked to a certain treatment which caused the same associations at higher taxonomic levels, or because multiple ASVs had the same treatment associations at higher taxonomic levels.

In some cases, single ASVs were associated with the same experimental conditions at different taxonomic levels. An ASV belonging to *Ralstonia* genus (*Gammaproteobacteria* class) was highly probable in one single topic that did not show any associations with treatments at phylum, class, and order levels. However, at family and ASV levels this ASV was the most probable (45.94% at family and 49% at ASV levels) in the topic linked to half-water treatment and generation 1 plants with forest soil microbiome inoculation. An ASV from *autotrophicum/hiltneri/lusitanum* species (*Gammaproteobacteria* class) was detected in topics related to half-water treatment at ASV (19.2%), family (8.6%) and order (16.3%) levels. An ASV from *Rhodobacter* genus (*Alphaproteobacteria* class) was prevalent in topics related to generation 0 and full-water treatment at ASV (7.1%), family (19.5%) and order (8.4%) levels. An ASV from *Vampirovibrio* genus (*Cyanobacteria* phylum) was detected in topics related to plants with agricultural soil microbiome inoculation at ASV (5.3 and 7.1%), family (37.5%), order (7.01%), and class (18.7%) levels. An ASV from *Pseudarthrobacter* genus (*Actinobacteria* class) was highly probable in topics associated with half-water treatment. There were several other ASVs from *Micrococcales* order that contributed to half-water associated topics but their probabilities were much smaller than that of *Pseudarthrobacter*.

Multiple ASVs appeared in the same topics associated with certain experimental conditions. Two ASVs from *Deinococcus* genus were highly probable in only one topic associated with generation 0 at each taxonomic level. Three ASVs from *Streptomyces* genus (*Actinobacteria* class) were abundant in the topic related to stable half-watering treatment and generation 1 plants with agricultural soil microbiome inoculation at order taxonomic level and lower. Four ASVs from *Sphingobacteriales* order (*Bacteroidia* class) were highly abundant in topics linked to half-water treatment and plants without soil microbiome inoculation at family and ASV taxonomic levels. Starting from order level, five ASVs in *Chitinophagacea* family (*Bacteroidia* class) were found in topics associated with half-water treatment, whereas two ASVs in *Dyadobacter* genus (*Bacteroidia* class) were found in topics associated with full-water treatment. At family and ASV taxonomic levels, two ASVs in *Microscillaceae* family (*Bacteroidia* class) were linked to topics related to plants with agricultural soil microbiome inoculation.

### 3.4 Correlation and differential abundance analysis

We saw significant Spearman’s correlations between learned topics and some plant morphological traits at each taxonomic level (Table 3). However, we did not find any statistically significant correlations between topics and plant functional traits, such as SCP and WUEi, except for one topic at ASV taxonomic level that was correlated with WUEi.

To compare the LDA approach with traditional microbiome analysis, we ran Spearman’s rank correlation tests between taxa and plant traits at both phylum and ASV levels. We used an indicator species analysis to find significant relationships between individual taxa and plant traits. We also conducted differential abundance analysis to identify taxa that significantly differed in abundance between treatments. Indicator species analysis did not identify any taxa significantly associated with any treatment following multiple test correction. Both correlation analyses done for individual phyla and on LDA topics mostly agreed with each other. Out of 3946 ASVs, 108 of them were found to have significant correlations with root biomass, drought time, stem height, stem diameter, and leaf mass per area (S19 and S20 Tables). At the phylum level, out of 27 phyla, 11 were found to have significant correlations with root biomass, drought time, stem height, and stem diameter (S3 Table). We expect to see taller plants, plants with increased leaf mass per area, and plants with the smaller values of the drought time and stem diameter for the plants grown under half-water treatment than under full-water treatment. Therefore, taxa abundant in water-limited soil were expected to be positively correlated with the stem height and negatively correlated with the drought time, stem diameter, and root biomass, and show the opposite behavior under the full-water treatment. On average, plants in generation 0 were taller, had thicker stems and larger root biomasses than generation 1 plants. Correlation analysis at the phylum level showed that *Actinobacteriota* was negatively correlated with the drought time. Both *Crenarchaeota* and *Bdellovibrionota* were negatively correlated with the stem diameter, *Crenarchaeota* was also negatively correlated with the root biomass, and *Bdellovibrionota* was positively correlated with the stem height. *Thermoplasmatota* was positively correlated with the stem diameter and root biomass and negatively correlated with the stem height. *Gemmatimonadota* and *Verrucomicrobiota* were positively correlated with the stem diameter and the drought time. *WPS-2* was positively correlated with the stem diameter and *Deinococcota* was positively correlated with the root biomass. These results obtained from the correlation analysis performed on individual taxa were in agreement with the results from LDA analysis. *Actinobacteriota*, *Bdellovibrionota*, *Crenarchaeota* amplified in half-water associated topic were negatively correlated with the stem diameter and positively correlated with the leaf mass per area. *Gemmatimonadota, Verrucomicrobiota*, and *WPS-2* were amplified only in the full-water treatment associated topic and this topic was negatively correlated with the leaf mass per area. *Deinococcota* was found probable in generation 0 associated topic which was positively correlated with root biomass and stem diameter, and negatively correlated with the stem height. LDA topic results mostly agreed with the results obtained from the correlation analysis performed on individual taxa and agreed with the bacteria abundance trends observed in other studies. Instead of analyzing individual taxa, LDA learned topics (groups of microbial communities) that may be associated with experimental conditions and correlated with plant traits. LDA analysis directly highlights communities in the microbiome that can be generically expected to be related to the conditions of the plant and the response of the plant to those conditions.

**Fig 8:**
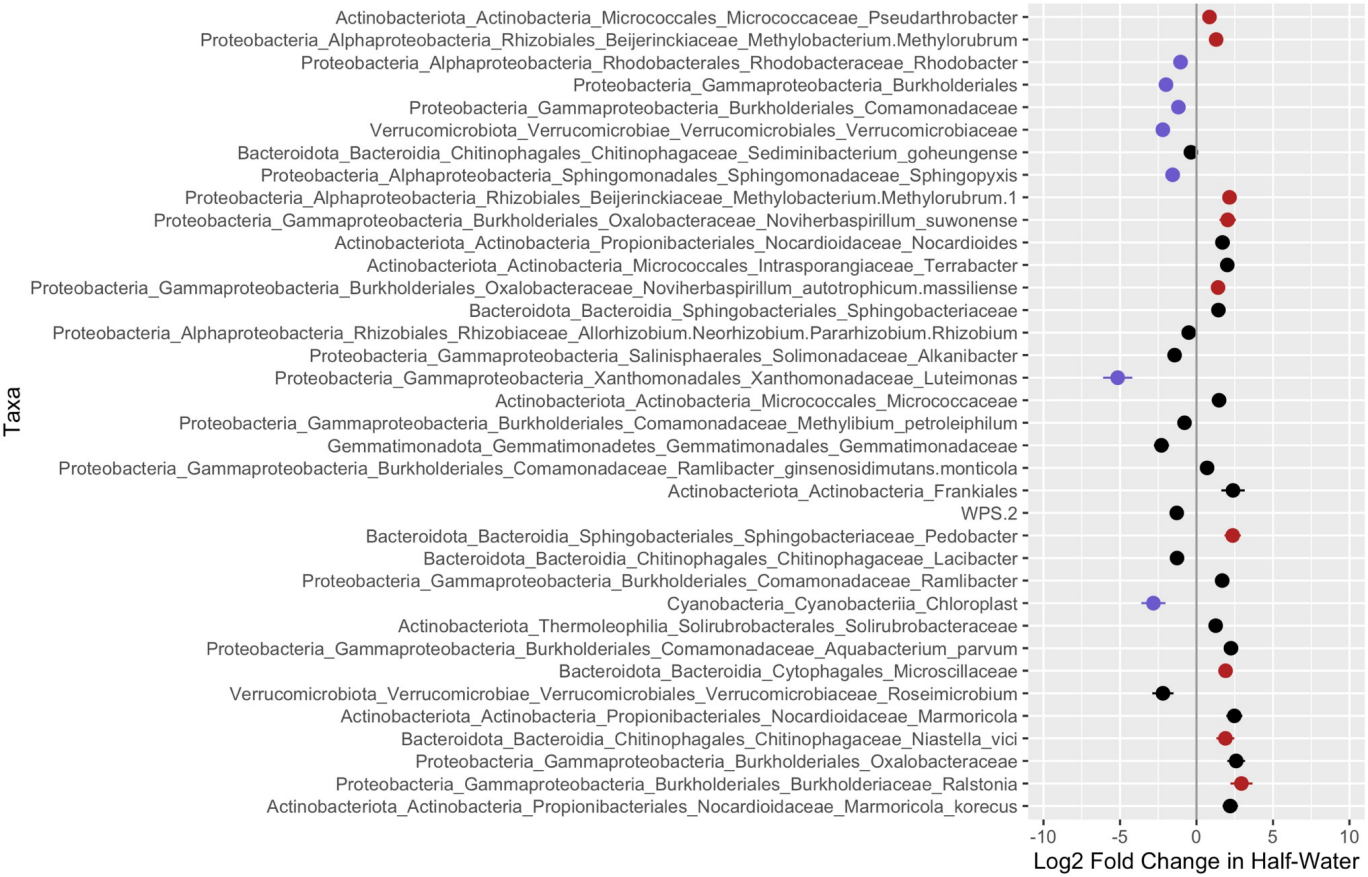
Difference in abundances of ASVs in the half-water treatment relative to the full-water (dashed middle line). Dots represent the differential abundance coefficient and the error bars are standard errors. The taxa shown are only those that are significant after a p-value correction with the FDR set to 0.05. Plots were produced using *corncob* R package [50]. The dots colored in red show taxa that were abundant in half-water related LDA topics. The dots colored in blue show taxa that were abundant in full water related LDA topics. Note that the comparison was made with the Fig 7 where only ASVs with *p* > 0.025 are shown. The second half of the plot with other significant taxa is shown in S12 Fig.

Differential abundance analysis showed plenty of significant ASVs that differ in abundance between treatments (Figs 8 and S12, S13 and S14). Both LDA and differential abundance analyses showed the same ASVs that were related to certain treatments. ASVs from genera such as *Pseudarthrobacter, Methylobacterium-Methylorubrum, Noviherbaspirillum, Pedobacter, Niastella, Ralstonia, SH-PL14* and ASV from *Microscillaceae* family were abundant in the half-water treatment relative to the full-water treatment in both LDA and differential abundance analyses. On the other hand, ASVs from genera such as *Rhodobacter, Sphingopyxis, Luteimonas, Caulobacteraceae*, ASV from *Comamonadaceae, Verrucomicrobiaceae* families, and ASV from *Burkholderiales, Cyanobacteria* orders were abundant in the full-water treatment relative to the half-water treatment in both LDA and differential abundance analyses. When comparing differential abundance analysis results with that from LDA we only considered ASVs with the probabilities larger than 0.025.

## 4 Discussion

To show the utility of LDA for analyzing environmental microbiomes, we performed analysis on two datasets: coral and maize microbiomes. The coral dataset was previously analyzed by Rosales et al. [19] using traditional methods for microbiome analysis. The results from LDA analysis mostly agreed with findings in [19]. We identified most of the same taxa linked to disease resistance. In particular, *Myxococcales (P3OB-42)* was dominant bacteria linked to visually unaffected samples. *Sphingobium yanoikuyae* was detected in topics related to disease-exposed and visually unaffected samples. LDA showed that even when a putative pathogen (*Sphingobium yanoikuyae*) was abundant, certain community structures could favor disease resistance compared to others. LDA also revealed additional associations of several taxa not mentioned by Rosales et al. [19] with disease resistance and susceptibility. The ASV from the *Halobacteroidaceae* family was the most probable bacteria in topic ascribed to visually unaffected samples from *A. Palmata* species. LDA showed that *Fulvivirga, Arcobacter*, and *Oscillatoria* genera were more dominant in topics related to high disease susceptibility in *A. Palmata* species.

The LDA analysis on our own maize dataset showed that microbial community topics were associated with the water treatment at each taxonomic level. In general, we found stronger relationships between topics and experimental conditions at lower taxonomic levels. This is to be expected because analysis at a high taxonomic level is not able to distinguish potential differences between groups belonging to the same high-level classification. Identifying taxonomic groups associated with the environmental conditions at the phylum level does not account for potential heterogeneity of plant traits when analyzing the microbiome at finer taxonomic levels; e.g. not all classes in a given phylum may have the same association to experimental treatments or responses. LDA analysis at finer taxonomic levels thus offers a way to characterize the uniformity of the function and environmental preferences of microbial groups (e.g. classes) belonging to a corresponding higher taxonomic group (e.g. phylum). We found that more topics were associated with generation 1 rather than generation 0. Between each generation, community composition was shifted; and some topics were strongly associated with one generation or another. We believe that was likely due to microbial communities adapting to the pot environment and selection upon the communities by maize [51]. Therefore, topics most associated with generation 1 could be a consequence of adaptation to the maize system, in response to drought stress, or a combination of both factors.

Most of the topics at lower taxonomic levels were associated with some treatment in the maize experiment. This may raise concerns about overfitting. However, the concept of overfitting has some subtitles for LDA. This is because LDA is an unsupervised method—the algorithm that constructs topics from microbiomes does not take experimental treatments and plant trait measurements into account whatsoever. LDA topics therefore cannot overfit to these variables, rather, the LDA topics are a representation of the data which is, in some sense, parsimonious, approximating the data matrix with a factorization into sparse sub-matrices.

We have used arbitrary thresholds to determine whether topics are moderately (2:1) or strongly (4:1) associated with an plant characteristic or experimental condition. Even though we used a 4:1 ratio, many topics ended up very strongly associated with experimental treatments at a 20:1 ratio (Fig 6), especially with water treatment, which is not surprising as water treatment affected maize far more than other experimental variables. By adjusting this threshold, one may see the different number of topic associations with experimental treatments or plant traits. We leave the careful mathematical establishment of a significant threshold for association to further work.

In addition to finding microbial communities associated with experimental conditions, LDA allowed us to connect the expression of certain plant traits to the topic distributions for a better understanding of plant-microbiome interaction. Statistically significant relationships were found between learned topics and plant structural traits. No statistically significant relationships were observed between learned topics and functional plant traits, except only one topic at ASV taxonomic level that was found to be statistically significant with WUEi. WUEi is a composite trait calculated as a ratio of the maximum rate of photosynthesis over the stomatal conductance, which were each measured separately. As such, the combination of measurement errors made it difficult to resolve functional differences in the plants, and this may explain why no significant microbiome associations were found for WUEi. As for SCP, we were unable to measure SCP values in more than half of the plants in generation 0. This might partially explain why no statistically significant relationships were observed between topics and SCP.

Our results are in agreement with the literature where data is available, mostly at the phylum level; for example Gram-positive phyla *Actinobacteriota* and *Firmicutes* were present in topics more abundant in samples exposed to half-water treatment, whereas *Verrucomicrobiota* was dominant in topics connected to full-water treatment. Previous studies have shown that these phyla are associated with these watering conditions in different host plants [21, 25]. There is limited information about which taxa are more associated with water-limited soil at lower taxonomic levels and therefore, verifying our results against previous studies is difficult. We note that even if some information about drought enriched taxa is available from several experiments for different plant hosts, it is not clear how such information relates to our experiment. Nevertheless, our analysis gives some results similar to previous studies of plant microbiomes at the phylum taxonomic level and gives rise to a host of hypotheses that might be tested in more targeted experiments.

In LDA, every topic contains everything but the probability distribution is different. We assessed the number of important taxa for each topic by examining the effective number of words metric, which quantifies how many taxa the topic is effectively spread across. Taxa below this rank are less important to the topic. While we found many significant relationships between topics and traits related to the size of the plant, it is not easy to conjecture what specific ecological functions to associate with each topic. Previous studies revealed links between microbiome content and chemical processes related to plant function both in droughted maize and tropical forests, including enzyme activites related to nitrogen cycling and the degradation of complex plant C, such as cellulose and lignin [52, 53]. Our results allow forming hypotheses on which microbes or microbial communities to choose for target experiments designed to identify the function of the microbial communities. We can only hypothesize how exactly identified microbial species within a topic, strongly associated with specific experimental conditions, may act synergistically towards a biological function across multiple environments. Microbial communities can improve the environmental adaptability and plant performance by contributing to plant protection against stress factors. The one question that may arise is whether the water treatment or the microbiome itself was the main driver of plant traits in our experiment. It is well known that water availability influences plant size [54], but the water treatment also affects the soil microbiome [55, 56]. Further controlled experiments may be useful to disentangle cause and effect between microbiome and plant response to drought.

In our experiment we analyzed 16S rRNA gene amplicon sequencing data. However, LDA can analyze different types of -omics data. LDA also avoids controversial data normalizations because normalization does not affect LDA results. LDA performs dimensionality reduction in a highly interpretable way, relating each sample to a position in low-dimensional “topic” space. Each learned LDA topic represents a probability distribution of microbial taxa that tend to co-occur together in data consistently. LDA analysis provides a different view into the host-microbiome interaction problem compared to the traditional microbiome correlation analysis which identifies relationships between the individual taxa and traits of the organism. LDA is able to affordably and conveniently find significant relationships without using only the most abundant taxa, as it correlates compressed individual microbiome abundances in topics with variables of interest (the longest analysis, at the ASV level, took about 10 hours on a single compute node). This illustrates how the LDA analysis directly highlights communities in the microbiome that can be generally expected to be related to the conditions of the environment and the response of the organism to those conditions. In this way, we may find the relationships that are significant in a different way than that when going through each taxon individually, paving way to effective identification of connections between microbial community composition and ecosystem function.

LDA infers topics representing taxa along with their probabilities, the taxa in each topic are non-mutually exclusive which is more appropriate in biological settings. This is because even if taxa are taxonomically related, they may or may not perform the same ecological function when clustered with other species [57–60]. Rather than looking at the taxa that are genetically similar that is belonging to the same taxonomic groups, LDA aggregates taxa and constructs topics according to co-occurence patterns in data. There is a lot of variability in microbiomes across samples coming from different environments. Compared to traditional microbiome analysis approaches which target individual taxa, the LDA analysis is capable of identifying groups of multiple taxa, that together, may form a cohesive module that is essential for an ecological function of interest, not only in one environmental context, but also perhaps across multiple environments, where the relevance of single taxa may change [61].

We note that in terms of systematic treatment-control experiments, it may be better to use a supervised approach such as hypothesis testing or to explore nonlinear regression using machine learning algorithms. LDA is more appropriate to observational datasets (such as the relationship between maize microbiome and plant trait) where the correlations are not between controlled variables—this is one of its strengths. LDA is useful for untangling the interaction between stressed organisms and their microbiomes, but precise procedures for doing so have not been firmly established in the literature, and future work may refine the workflow we present here.

## 5 Conclusions

We explored how data-driven Latent Dirichlet Allocation and taxonomic classification can be combined to explore the content of complex datasets of coral microbiomes and maize soil microbiome samples. By showing the successful LDA application on a previously published coral microbiome data, we then analyzed our maize data at different taxonomic levels. We detected microbial community topics that were associated with different experimental conditions, such as water-limited soil or soil source type, and found multiple topics that were connected to plant traits.

LDA represents data in a compact, unsupervised way and is able to conveniently find relationships between microbial community topics and traits of organism and environmental conditions. This is far simpler than exploring each taxon individually. The main advantage of using LDA is that this method not only identifies bacteria that are enriched or depleted under under certain conditions; but rather non-mutually exclusive communities of microbial species that are not necessarily taxonomically related but may act together towards some ecological function. This study shows that LDA is a powerful tool for analyzing environmental microbial communities as it can generate novel insights from biodiversity data.

## Supporting information

S1 Text Dataset and Experiment. (PDF)

S1 Fig Averaged perplexity score for cross-validation folds, pairwise cosine similarity between taxa in topics, coherence, and exclusivity of the most abundant taxa in the topics. (PDF)

S2 Fig Phylum level. Topic abundance weighting for the water treatment and generation, and for the different soil microbiome inoculation source types.
(PDF)

S3 Fig Phylum level. Distribution of phyla in each learned LDA topic.
(PDF)

S4 Fig Class level. Topic abundance weighting for the water treatment and generation, and for the different soil microbiome inoculation source types.
(PDF)

S5 Fig Class level. Distribution of classes in each learned LDA topic.
(PDF)

S6 Fig Order level. Topic abundance weighting for the water treatment and generation, and for the different soil microbiome inoculation source types.
(PDF)

S7 Fig Order level. Distribution of orders in each learned LDA topic.
(PDF)

S8 Fig Family level. Topic abundance weighting for the water treatment and generation, and for the different soil microbiome inoculation source types.
(PDF)

S9 Fig Family level. Distribution of families in each learned LDA topic.
(PDF)

S10 Fig ASV level. Distribution of ASVs in each learned LDA topic. Topics are ordered by the association with the soil source inoculation type.
(PDF)

S11 Fig ASV level. Distribution of ASVs in each learned LDA topic. Topics are ordered by the association with the water stability treatment.
(PDF)

S12 Fig ASV level. Difference in abundances of ASVs in the half-water treatment relative to the full-water (dashed middle line).
(PDF)

S13 Fig ASV level. Difference in abundances of ASVs in the forest soil source inoculation and soil without inoculation relative to the agricultural soil source inoculation.
(PDF)

S14 Fig ASV level. Difference in abundances of ASVs in the generation 1 plants relative to the generation 0 plants (dashed middle line).
(PDF)

S1 Table Phylum level. Probability distribution of phyla in each LDA topic.
(PDF)

S2 Table Phylum level. Relative amplifications of phyla in each LDA topic.
(PDF)

S3 Table Phylum level. Statistically significant differences between 17 phyla and plant traits based on Spearman’s rank correlation coefficient with Holm–Bonferroni correction.
(PDF)

S4 Table Phylum level. Statistically significant relationships between topics and plant traits based on Spearman’s rank correlation coefficient with Holm–Bonferroni correction.
(PDF)

S5 Table Phylum level. Consistency of the topics from the different LDA runs.
(PDF)

S6 Table Phylum level. Effect of using different number of topics at phylum level.
(PDF)

S7 Table Class level. Probability distribution of classes in each LDA topic.
(PDF)

S8 Table Class level. Relative amplifications of classes in each LDA topic.
(PDF)

S9 Table Class level. Statistically significant relationships between topics and plant traits based on Spearman’s rank correlation coefficient with Holm–Bonferroni correction.
(PDF)

S10 Table Order level. Probability distribution of orders in each LDA topic.
(PDF)

S11 Table Order level. Relative amplifications of orders in each LDA topic.
(PDF)

S12 Table Order level. Statistically significant relationships between topics and plant traits based on Spearman’s rank correlation coefficient with Holm–Bonferroni correction.
(PDF)

S13 Table Family level. Probability distribution of families in each LDA topic.
(PDF)

S14 Table Family level. Relative amplifications of families in each LDA topic.
(PDF)

S15 Table Family level. Statistically significant relationships between topics and plant traits based on Spearman’s rank correlation coefficient with Holm–Bonferroni correction.
(PDF)

S16 Table ASV level. Probability distribution of ASVs in each LDA topic.
(PDF)

S17 Table ASV level. Relative amplifications of ASVs in each LDA topic.
(PDF)

S18 Table ASV level. Statistically significant relationships between topics and plant traits based on Spearman’s rank correlation coefficient with Holm–Bonferroni correction.
(PDF)

S19 Table ASV level. Statistically significant differences between 212 ASVs and plant traits based on Spearman’s rank correlation coefficient with Holm–Bonferroni correction. Part 1.
(PDF)

S20 Table ASV level. Statistically significant differences between 212 ASVs and plant traits based on Spearman’s rank correlation coefficient with Holm–Bonferroni correction. Part 2.
(PDF)
