# Supplementary material for: Latent Dirichlet Allocation modeling of environmental microbiomes
Source: PLoS Comput Biol. 2023 Jun 8;19(6):e1011075. doi: 10.1371/journal.pcbi.1011075 (PMC10249879; doi:10.1371/journal.pcbi.1011075)
Supplement: S1 Text — (PDF) [file pcbi.1011075.s001.pdf]

Dataset and Experiment

In this work, we present results from data on 119 microbiome-plant systems from two generations (called generation 0 and 1) of serial microbiome propagation experiment. These experiments were performed in order to understand the characteristics of the system, and effects of different watering treatments on plant-microbiome interactions with the system. In each generation 64 maize plants were grown in a greenhouse setting between January and May 2020 in individual 2.6 gal (9.8 L) pots filled with 6L of fritted clay (GreensGrade, 20-50 mesh size: Profile, Buffalo Grove, IL, USA). Fritted clay is a baked ceramic used for land improvement and as a sterile growth medium for plant-microbiome experiments [1]. Prior to planting, the fritted clay in each pot was washed three times with filtered tap water to remove excess nutrients [2]. The greenhouse temperature was controlled to stay between 20-35°C all the time. To equalize the daylight hours, natural light was supplemented with growth lights (Grower's Choice model ROI-E720 (Grower's Choice, Ontario, CA, USA) set to 50% of maximum intensity to produce PPF 1000 μmol/m^2/s) both in the morning and evening to create a 14h/10h day/night cycle. The plant seed for each pot in each generation was randomly drawn from a stock of an experimental strain of maize, USDA seed bank inbred line "B73". For generation 0, 48 of the seeds were inoculated by microbial communities [3] originating from one of two natural soils collected from an agricultural field near Akron, CO, USA or a ponderosa pine forest near Los Alamos, NM, USA, and 16 seeds were planted without inoculation to form a control treatment. After seed germination, the plants were divided into two watering treatments: well watered (up to 65% volumetric water content 3 times a week with filtered tap water fortified with General Hydroponics (Santa Rosa, CA, USA) FloraGro, FloraMicro, and FloraBloom fertilizers with doses 200, 200 and 100 μL/L of water, respectively) and half watered (up to 45% volumetric water content 3 times a week with similar fertilization as with full water treatment). The watering treatments were selected based on tests with fritted clay prior to the experiment to represent no water limitation (watering to field capacity (65%)) and a mild drought where soil moisture content would drop below the limit of water access to plants between the waterings, but would not significantly limit plant growth [4, 1]. Before each watering, volumetric water content of each pot was measured with a soil moisture probe (Teros 10, Meter Environment, Pullman WA, USA) installed in the middle of the pot, and the water addition needed to reach the target water content was calculated using a water retention curve determined for fritted clay in our system during tests conducted before the experiment. After the plants had grown to a stage showing 9 fully grown leaves ($\sim$ 8 weeks), the watering was stopped and the plants were allowed to dry under complete water withdrawal until stomatal closure occurred (terminal drought). Before the terminal drought, the soil in each pot was sampled for microbiome and soil chemistry analysis, plants were sampled for leaf chemistry analysis, and measured for height, stem diameter, maximum photosynthesis rate, and stomatal conductance (Infrared gas analyser; Licor 6400, Licor Inc. (Lincoln, NE, USA)) from which water use efficiency was calculated by dividing the maximum photosynthesis rate by the measured stomatal conductance. During terminal drought, stomatal conductance of each plant was measured daily. When stomatal conductance reached zero (verified with LI6400 infrared gas analyzer, Licor Inc. (Lincoln, NE, USA) using ambient temperature, CO_2 concentration and relative humidity with saturation light), a leaf was cut for leaf water potential measurements using pressure chamber method (pressure chamber model 1005, PMS Instrument Company (Albany, OR, USA)) to determine the stomatal closure point (leaf water potential at which the plant closes stomata), and the time since the beginning of the terminal drought was

recorded (drought time). For generation 1, new pots with fritted clay were set up, and new seeds were inoculated by serially transferring the microbial communities from the generation 0 pots with no selection. Therefore, plants in generation 1 were growing with the microbiomes that were direct descendants of those from generation 0. The same watering treatments as in generation 0 were imposed after seed germination, but now so that for half of the plants in each treatment the watering treatment was switched. This created two additional treatment categories (stable watering vs. switched watering) for microbiomes from each original soil source and the non-inoculated controls to form a total of 6 treatments in generation 0 (3 soil source microbiome inoculations and 2 watering treatments) and 12 treatments in generation 1 (3 soil source microbiome inoculations and 4 watering treatments (stable half-watering, switched half-watering, stable full-watering, switched full-watering)). Similarly to generation 0, generation 1 plants were grown to a stage showing 9 fully grown leaves ($\sim$ 8 weeks), and the plant performance measurements and the terminal drought treatment were conducted as for generation 0. Of our original 128 microbiome samples, 9 samples were omitted from the final analysis due to insufficient DNA, sequencing failures, or low read quality and quantity, resulting in the analysis of communities from only 119 plants. Due to the high density of roots in the pot, all of the soil was considered "rhizosphere", thus we chose to use soil cores to sample the rhizosphere community in the pots, rather than performing a separate analysis on soil directly adhered to roots, and because they were a more appropriate reflection of the communities we transferred to the 152 next generation. We collected one core (roughly 10cm deep) from each pot 4-5 cm from the plant using a sterile pipette tube as the bore. The plant was not removed (we removed the plant after collecting this for root morphology analysis). Prior to the experiment described here, we tested for the homogeneity of the microbiome in the pots by taking three cores from each pot. Comparison of the microbiomes from these cores did not reveal significant differences in diversity or species composition. Therefore, we continued collecting one core per pot.

References

1. Steinberg SL, Kluitenberg GJ, Jones SB, Daidzic NE, Reddi LN, Xiao M, et al. Physical and hydraulic properties of baked ceramic aggregates used for plant growth medium. Journal of the American Society for Horticultural Science. 2005;130(5):767–774.

2. Adams C, Jacobson A, Bugbee B. Ceramic aggregate sorption and desorption chemistry: implications for use as a component of soilless media. Journal of Plant Nutrition. 2014;37(8):1345–1357.

3. Ulrich DE, Sevanto S, Ryan M, Albright MB, Johansen RB, Dunbar JM. Plant-microbe interactions before drought influence plant physiological responses to subsequent severe drought. Scientific reports. 2019;9(1):1–10.

4. Van Bavel C, Lascano R, Wilson D. Water relations of fritted clay. Soil Science Society of America Journal. 1978;42(4):657–659.
