# Supplementary material for: Latent Dirichlet Allocation modeling of environmental microbiomes
Source: PLoS Comput Biol. 2023 Jun 8;19(6):e1011075. doi: 10.1371/journal.pcbi.1011075 (PMC10249879; doi:10.1371/journal.pcbi.1011075)
Supplement: S1 Fig — (PDF) [file pcbi.1011075.s002.pdf]

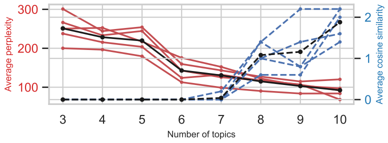

(a) Perplexity for cv folds and cosine similarity between taxa in topics at the phylum level.

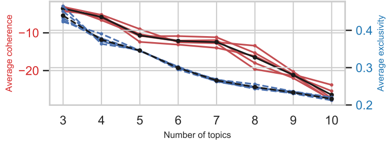

(b) Coherence and exclusivity of the most abundant taxa in the topics at the phylum level.

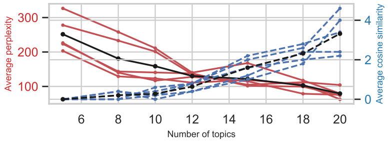

(c) Perplexity for cv folds and cosine similarity between taxa in topics at the class level.

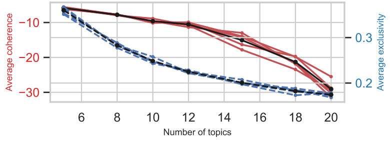

(d) Coherence and exclusivity of the most abundant taxa in the topics at the class level.

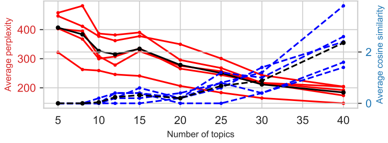

(e) Perplexity for cv folds and cosine similarity between taxa in topics at the order level.

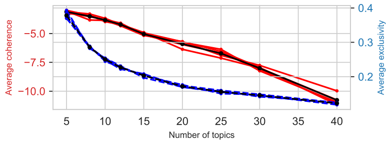

(f) Coherence and exclusivity of the most abundant taxa in the topics at the order level.

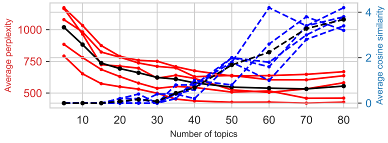

(g) Perplexity for cv folds and cosine similarity between taxa in topics at the family level.

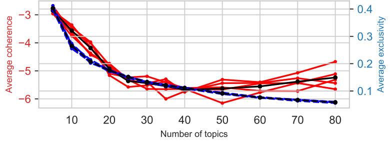

(h) Coherence and exclusivity of the most abundant taxa in the topics at the family level.

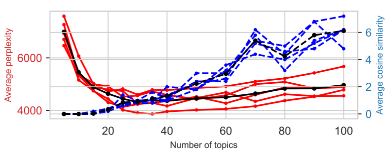

(i) Perplexity for cv folds and cosine similarity between taxa in topics at the ASV level.

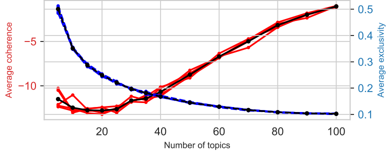

(j) Coherence and exclusivity of the most abundant taxa in the topics at the ASV level.

Figure 1: Averaged perplexity score for cross-validation folds, pairwise cosine similarity between taxa in topics, coherence, and exclusivity of the most abundant taxa in the topics. All topic modeling metrics are measured across 5-folds as functions of number of topics applied at the class, order, family, and ASV levels. Averaged curves over 5 different runs are shown in black.
