# Supplementary figures and images for: Latent Dirichlet Allocation modeling of environmental microbiomes
Source: PLoS Comput Biol. 2023 Jun 8;19(6):e1011075. doi: 10.1371/journal.pcbi.1011075 (PMC10249879; doi:10.1371/journal.pcbi.1011075)

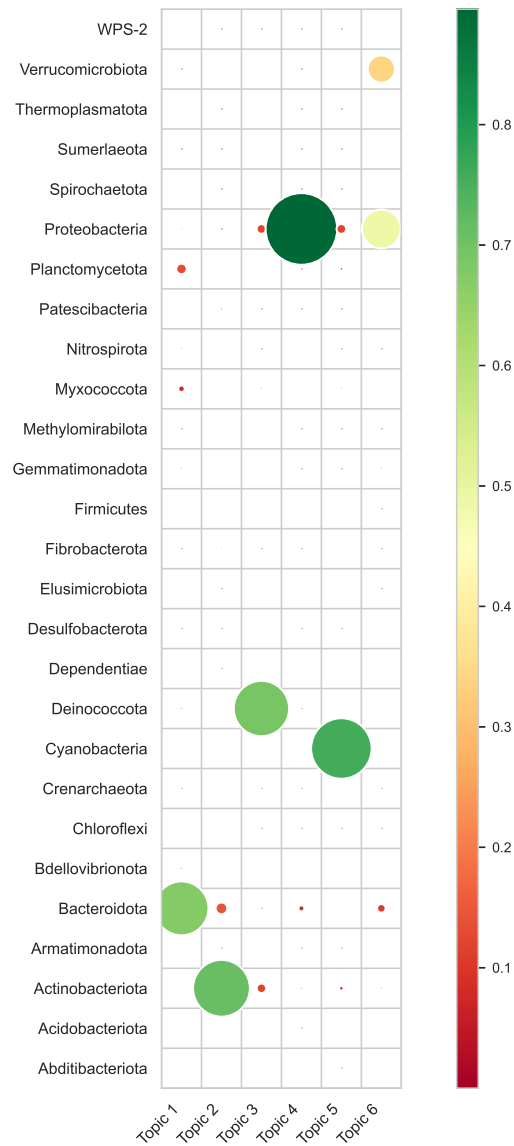

Figure 3: Distribution of phyla in each learned LDA topic.

Supplement: S3 Fig — Distribution of phyla in each learned LDA topic. (PDF) [file pcbi.1011075.s004.pdf]
