# Supplementary material for: Latent Dirichlet Allocation modeling of environmental microbiomes
Source: PLoS Comput Biol. 2023 Jun 8;19(6):e1011075. doi: 10.1371/journal.pcbi.1011075 (PMC10249879; doi:10.1371/journal.pcbi.1011075)
Supplement: S5 Fig — Distribution of classes in each learned LDA topic. (PDF) [file pcbi.1011075.s006.pdf]

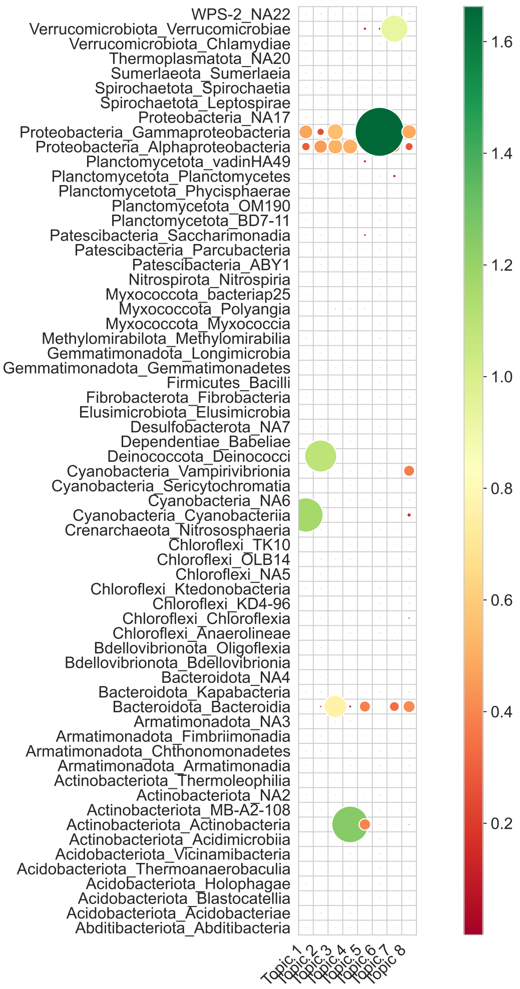

Figure 5: Distribution of classes in each learned LDA topic. The labels are written in the *phylum_class* format. The sizes of the circles representing probabilities are multiplied by 2 for visualization purposes.
