# Supplementary material for: Latent Dirichlet Allocation modeling of environmental microbiomes
Source: PLoS Comput Biol. 2023 Jun 8;19(6):e1011075. doi: 10.1371/journal.pcbi.1011075 (PMC10249879; doi:10.1371/journal.pcbi.1011075)
Supplement: S8 Fig — Topic abundance weighting for the water treatment and generation, and for the different soil microbiome inoculation source types. (PDF) [file pcbi.1011075.s009.pdf]

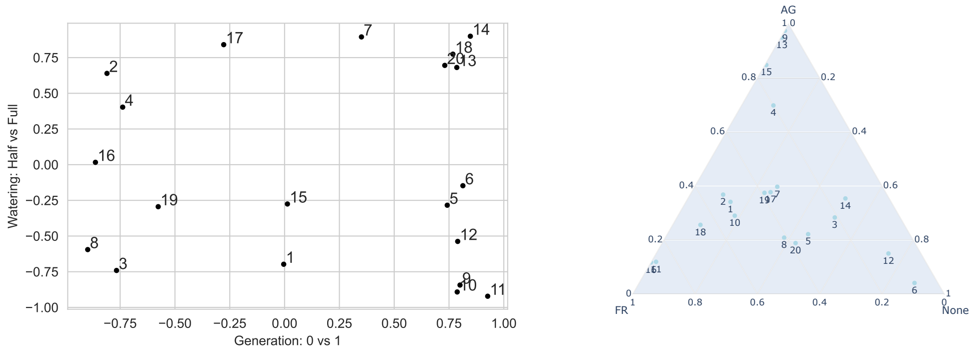

(a) Topic abundance weighting for the water treatment and generation.

(b) Topic abundance weighting for the different soil microbiome inoculation source types.

Figure 8: *Family level*. Topic abundance weighting for the water treatment and generation, and for the different soil microbiome inoculation source types.
