# Supplementary material for: Latent Dirichlet Allocation modeling of environmental microbiomes
Source: PLoS Comput Biol. 2023 Jun 8;19(6):e1011075. doi: 10.1371/journal.pcbi.1011075 (PMC10249879; doi:10.1371/journal.pcbi.1011075)
Supplement: S9 Fig — Distribution of families in each learned LDA topic. (PDF) [file pcbi.1011075.s010.pdf]

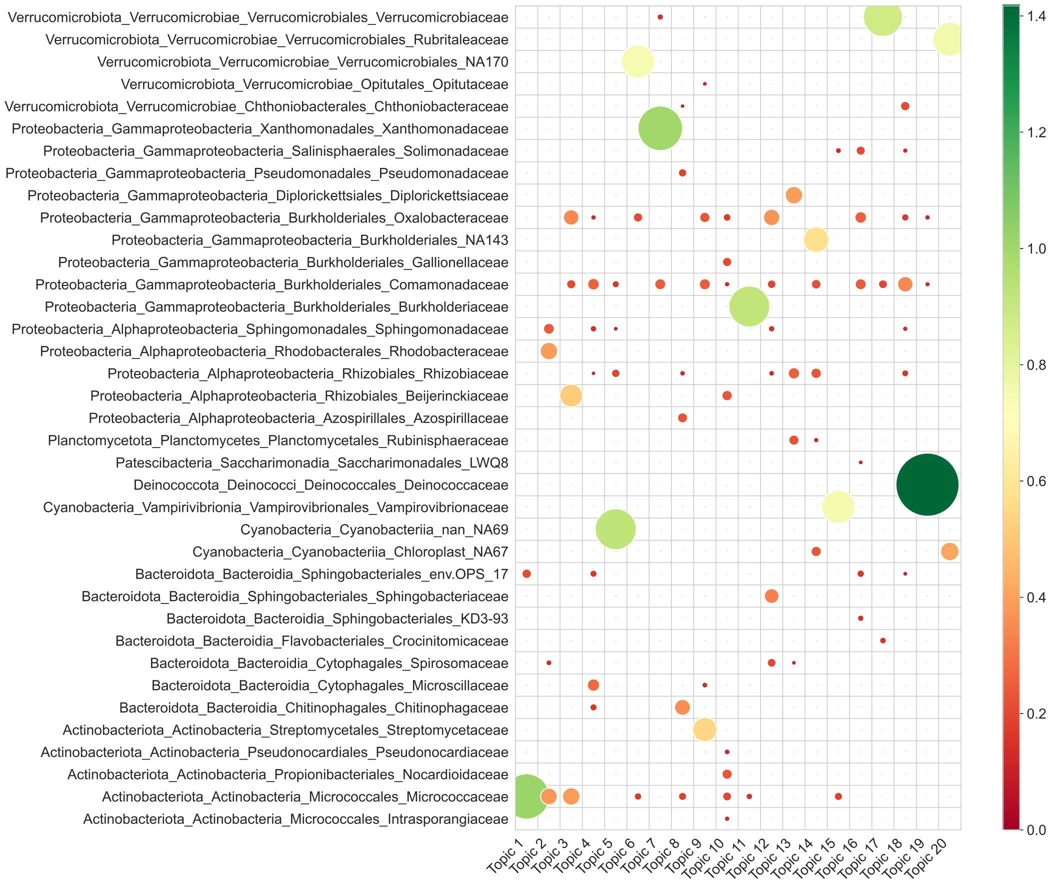

Figure 9: Distribution of families in each learned LDA topic. The labels are written in the *phylum_class_order_family* format. Only probabilities greater than 0.05 are shown. The sizes of the circles representing probabilities are multiplied by 2 for visualization purposes.
