# Supplementary material for: Latent Dirichlet Allocation modeling of environmental microbiomes
Source: PLoS Comput Biol. 2023 Jun 8;19(6):e1011075. doi: 10.1371/journal.pcbi.1011075 (PMC10249879; doi:10.1371/journal.pcbi.1011075)
Supplement: S10 Fig — Distribution of ASVs in each learned LDA topic. Topics are ordered by the association with the soil source inoculation type. (PDF) [file pcbi.1011075.s011.pdf]

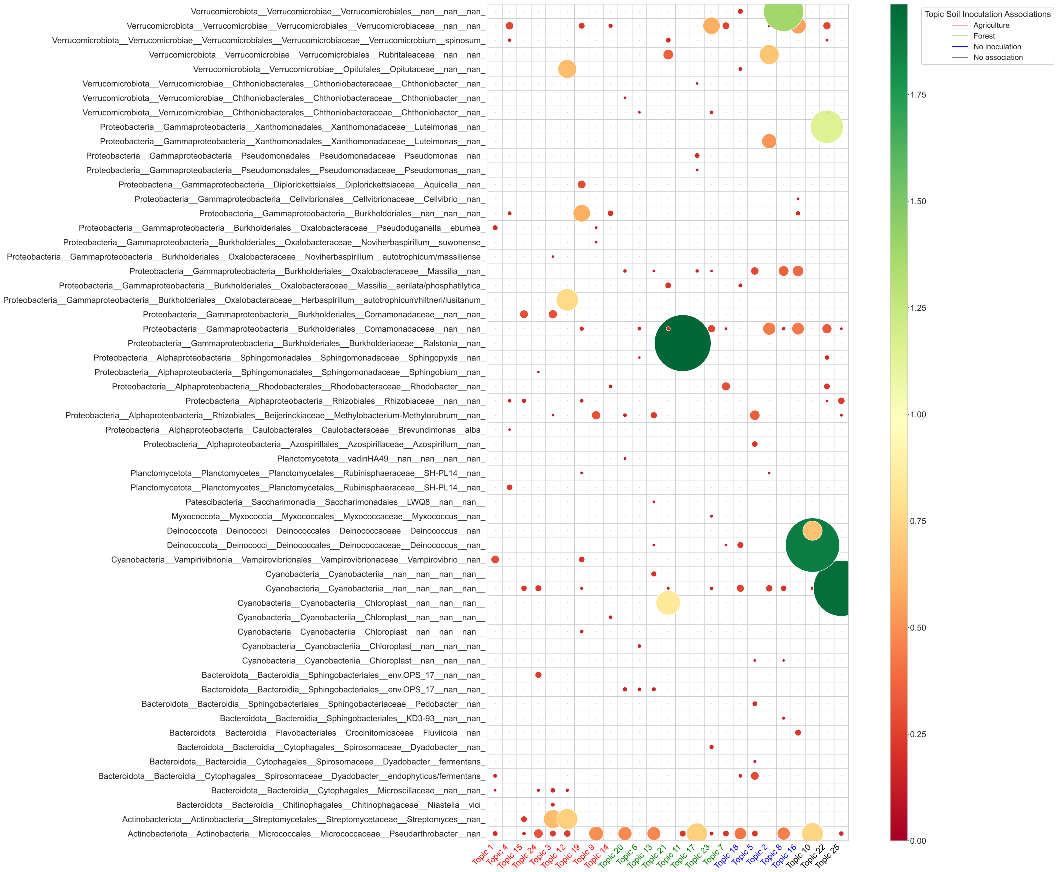

Figure 10: *ASV level*. Distribution of ASVs in each learned LDA topic. Topics are ordered by the association with the soil source inoculation type. The labels are written in the *phylum_class_order_family_genus_species* format, the names of genera, species, and ASV are not shown. Only probabilities greater than 0.025 are shown. The sizes of the circles representing probabilities are multiplied by 4 for visualization purposes.
