# Supplementary material for: Latent Dirichlet Allocation modeling of environmental microbiomes
Source: PLoS Comput Biol. 2023 Jun 8;19(6):e1011075. doi: 10.1371/journal.pcbi.1011075 (PMC10249879; doi:10.1371/journal.pcbi.1011075)
Supplement: S12 Fig — Difference in abundances of ASVs in the half-water treatment relative to the full-water (dashed middle line). (PDF) [file pcbi.1011075.s013.pdf]

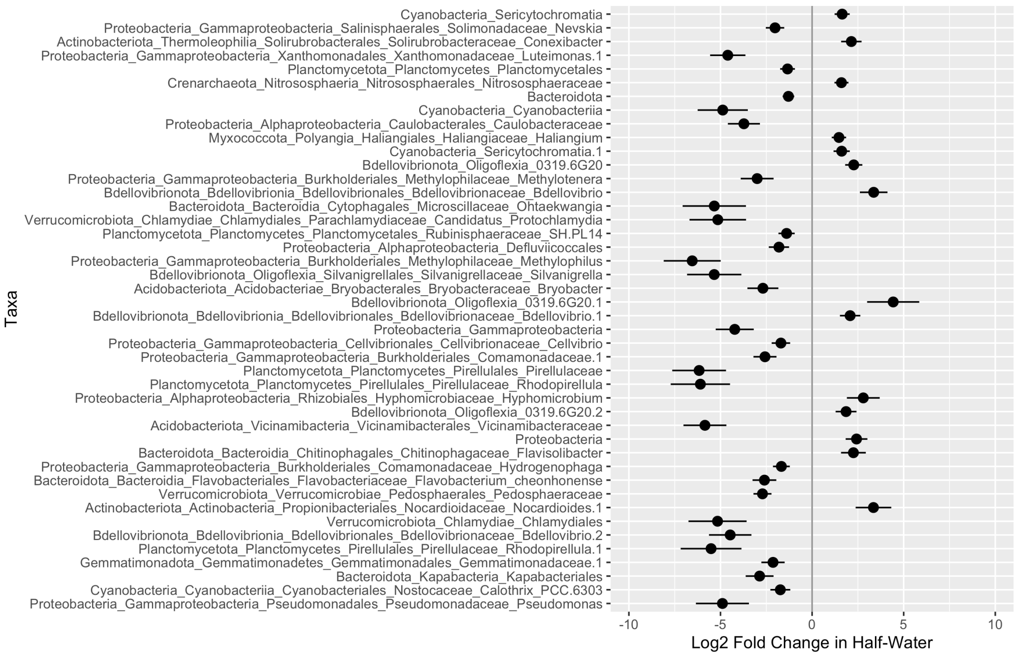

Figure 12: *ASV level.* Difference in abundances of ASVs in the half-water treatment relative to the full-water (dashed middle line). Dots represent the differential abundance coefficient and the error bars are standard errors. The taxa shown are only those that are significant after a p-value correction with the FDR set to 0.05. Plots were produced using *corncob* R package. Note: the other part of the plot is shown in the main text.
