# Supplementary material for: Latent Dirichlet Allocation modeling of environmental microbiomes
Source: PLoS Comput Biol. 2023 Jun 8;19(6):e1011075. doi: 10.1371/journal.pcbi.1011075 (PMC10249879; doi:10.1371/journal.pcbi.1011075)
Supplement: S13 Fig — Difference in abundances of ASVs in the forest soil source inoculation and soil without inoculation relative to the agricultural soil source inoculation. (PDF) [file pcbi.1011075.s014.pdf]

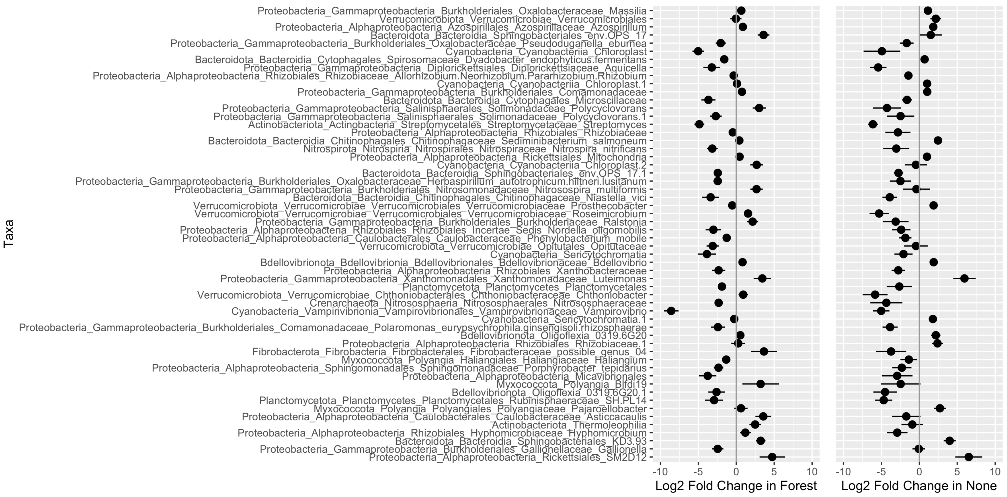

Figure 13: *ASV level*. Difference in abundances of ASVs in the forest soil source inoculation and soil without inoculation relative to the agricultural soil source inoculation. Dots represent the differential abundance coefficient and the error bars are standard errors. The taxa shown are only those that are significant after a p-value correction with the FDR set to 0.05. Plots were produced using *corncob* R package.
