# Supplementary material for: Latent Dirichlet Allocation modeling of environmental microbiomes
Source: PLoS Comput Biol. 2023 Jun 8;19(6):e1011075. doi: 10.1371/journal.pcbi.1011075 (PMC10249879; doi:10.1371/journal.pcbi.1011075)
Supplement: S14 Fig — Difference in abundances of ASVs in the generation 1 plants relative to the generation 0 plants (dashed middle line). (PDF) [file pcbi.1011075.s015.pdf]

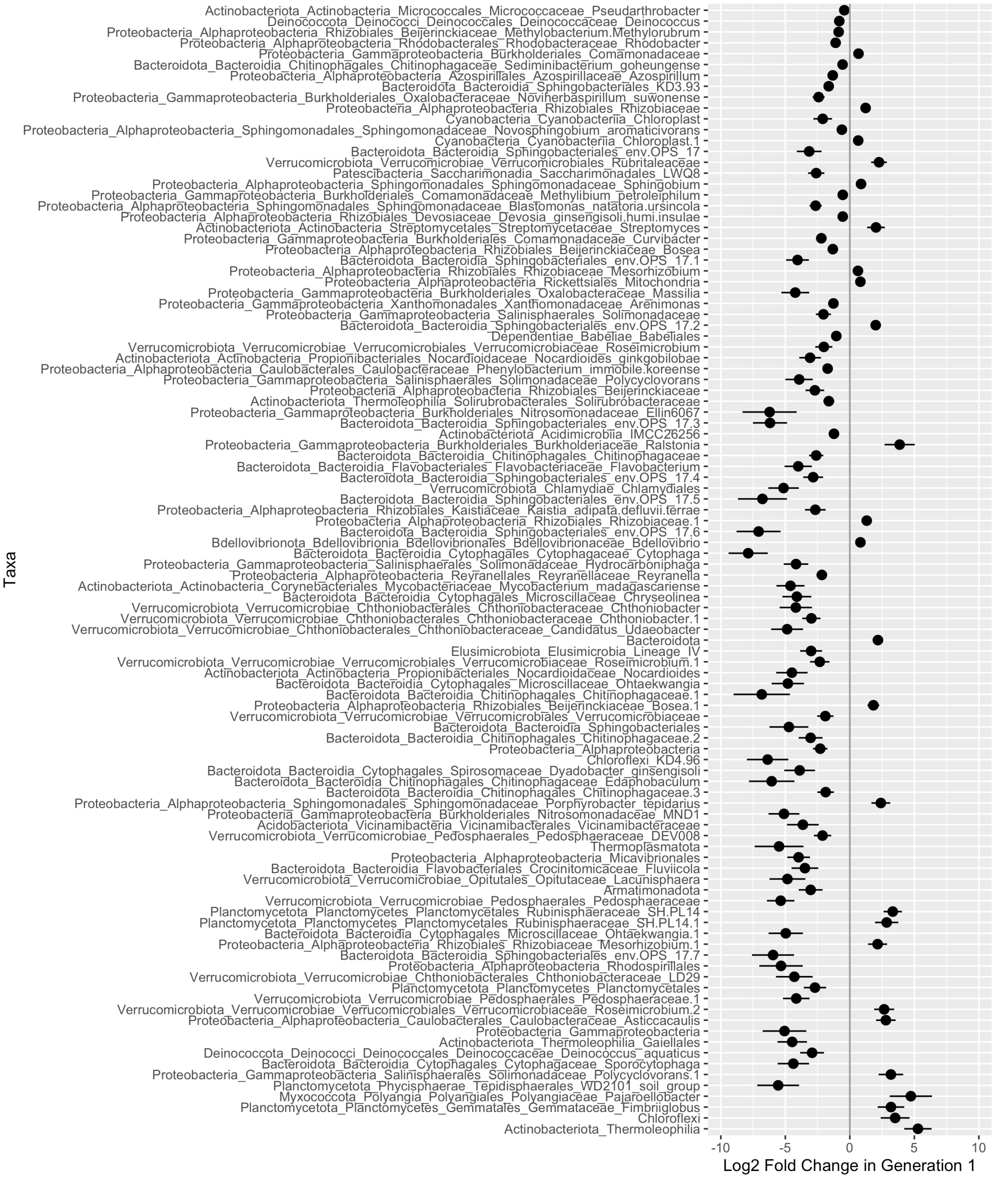

Figure 14: *ASV level*. Difference in abundances of ASVs in the generation 1 plants relative to the generation 0 plants (dashed middle line). Dots represent the differential abundance coefficient and the error bars are standard errors. The taxa shown are only those that are significant after a p-value correction with the FDR set to 0.05. Plots were produced using *corncob* R package.
