# Supplementary material for: Latent Dirichlet Allocation modeling of environmental microbiomes
Source: PLoS Comput Biol. 2023 Jun 8;19(6):e1011075. doi: 10.1371/journal.pcbi.1011075 (PMC10249879; doi:10.1371/journal.pcbi.1011075)
Supplement: S1 Table — Probability distribution of phyla in each LDA topic. (PDF) [file pcbi.1011075.s016.pdf]

	Topic 1	Topic 2	Topic 3	Topic 4	Topic 5	Topic 6
Abditibacteriota	-	-	0.212	0.084	-	-
Acidobacteriota	2.411	-	2.100	-	0.627	-
Actinobacteriota	-	**71.042**	11.867	1.369	5.523	0.975
Armatimonadota	-	-	0.154	-	-	-
Bacteroidota	**67.467**	**14.689**	-	7.447	-	**10.704**
Bdellovibrionota	0.914	1.816	-	0.436	0.080	0.452
Chloroflexi	2.274	-	-	-	-	-
Crenarchaeota	-	0.538	-	-	0.067	-
Cyanobacteria	-	1.742	2.589	-	**75.708**	0.394
Deinococcota	0.833	-	**69.236**	-	0.097	-
Dependentiae	-	-	0.205	0.061	-	-
Elusimicrobiota	-	-	-	0.133	0.060	-
Fibrobacterota	-	0.658	-	-	-	-
Firmicutes	-	0.478	-	0.028	-	-
Gemmatimonadota	0.881	-	0.132	-	-	1.412
Myxococcota	8.204	1.954	1.191	0.145	1.443	0.590
Nitrospirota	1.021	-	-	0.698	-	-
Patescibacteria	-	3.603	-	-	-	-
Planctomycetota	**13.140**	-	-	-	4.219	1.566
Proteobacteria	1.300	-	**12.035**	**89.586**	**12.145**	**48.668**
Verrucomicrobiota	-	2.586	-	-	-	**33.929**
WPS-2	-	-	-	-	-	0.502

Table 1: *Phylum level.* Probability distribution of phyla in each LDA topic. Only ten most probable phyla in each topic are shown. Probabilities were converted to percentages. Effective taxa for each topic highlighted in bold.
