# Supplementary material for: Latent Dirichlet Allocation modeling of environmental microbiomes
Source: PLoS Comput Biol. 2023 Jun 8;19(6):e1011075. doi: 10.1371/journal.pcbi.1011075 (PMC10249879; doi:10.1371/journal.pcbi.1011075)
Supplement: S2 Table — Relative amplifications of phyla in each LDA topic. (PDF) [file pcbi.1011075.s017.pdf]

	Topic 1	Topic 2	Topic 3	Topic 4	Topic 5	Topic 6
Abditibacteriota	-	-	8.895	14.853	-	-
Acidobacteriota	8.061	-	10.365	-	7.756	-
Actinobacteriota	-	13.576	2.193	1.064	2.557	0.255
Armatimonadota	-	-	4.235	-	-	-
Bacteroidota	8.076	2.685	-	5.537	-	2.680
Bdellovibrionota	2.364	7.170	-	6.996	0.767	2.447
Chloroflexi	16.120	-	-	-	-	-
Crenarchaeota	-	14.947	-	-	4.501	-
Cyanobacteria	-	0.543	0.781	-	57.220	0.168
Deinococcota	0.276	-	33.802	-	0.119	-
Dependentiae	-	-	5.443	6.813	-	-
Elusimicrobiota	-	-	-	19.976	5.367	-
Fibrobacterota	-	16.101	-	-	-	-
Firmicutes	-	11.992	-	2.847	-	-
Gemmatimonadota	3.201	-	0.710	-	-	10.736
Myxococcota	9.070	3.299	1.945	0.996	5.903	1.364
Nitrospirota	4.274	-	-	18.152	-	-
Patescibacteria	-	15.099	-	-	-	-
Planctomycetota	10.631	-	-	-	12.628	2.650
Proteobacteria	0.048	-	0.659	20.630	1.666	3.774
Verrucomicrobiota	-	0.714	-	-	-	12.831
WPS-2	-	-	-	-	-	13.288

Table 2: *Phylum level.* Relative amplifications of phyla in each LDA topic. Only ten most amplified phyla in each topic are shown. Amplifications were converted to percentages.
