# Supplementary material for: Latent Dirichlet Allocation modeling of environmental microbiomes
Source: PLoS Comput Biol. 2023 Jun 8;19(6):e1011075. doi: 10.1371/journal.pcbi.1011075 (PMC10249879; doi:10.1371/journal.pcbi.1011075)
Supplement: S3 Table — Statistically significant differences between 17 phyla and plant traits based on Spearman’s rank correlation coefficient with Holm–Bonferroni correction. (PDF) [file pcbi.1011075.s018.pdf]

phylum	plant trait	correlation	p-value
Crenarchaeota	Stem_Diam	-0.496454	9.354124e-09
Thermoplasmatota	Stem_Diam	0.469794	7.038374e-08
Actinobacteriota	DroughtTime	-0.462793	1.162851e-07
Crenarchaeota	RootDry_g	-0.446124	3.677013e-07
WPS-2	Stem_Diam	0.443348	4.427962e-07
Gemmatimonadota	Stem_Diam	0.416235	2.500134e-06
Gemmatimonadota	DroughtTime	0.414275	2.817134e-06
Elusimicrobiota	Height_cm	-0.402450	5.699123e-06
Verrucomicrobiota	Stem_Diam	0.394602	8.964858e-06
Verrucomicrobiota	DroughtTime	0.389987	1.163888e-05
Sumerlaeota	Stem_Diam	0.380931	1.920937e-05
Bdellovibrionota	Stem_Diam	-0.380843	1.930135e-05
Thermoplasmatota	RootDry_g	0.372435	3.032923e-05
Dependentiae	Stem_Diam	0.368467	3.738000e-05
Bdellovibrionota	Height_cm	0.365381	4.389347e-05
Deinococcota	RootDry_g	0.354928	7.473403e-05
Thermoplasmatota	Height_cm	-0.337332	1.757123e-04

Table 3: *Phylum level.* Statistically significant differences between 17 phyla (11 are unique) and plant traits based on Spearman's rank correlation coefficient with Holm–Bonferroni correction.
