# Supplementary material for: Latent Dirichlet Allocation modeling of environmental microbiomes
Source: PLoS Comput Biol. 2023 Jun 8;19(6):e1011075. doi: 10.1371/journal.pcbi.1011075 (PMC10249879; doi:10.1371/journal.pcbi.1011075)
Supplement: S4 Table — Statistically significant relationships between topics and plant traits based on Spearman’s rank correlation coefficient with Holm–Bonferroni correction. (PDF) [file pcbi.1011075.s019.pdf]

topic	plant trait	correlation	p-value
Topic 3	Root biomass	0.381000	0.000019
Topic 2	Leaf mass per area	0.325778	0.000300
Topic 3	Stem diameter	0.324194	0.000322
Topic 3	Stem height	-0.314657	0.000492
Topic 4	Stem diameter	-0.309939	0.000603
Topic 4	Stomatal conductance	-0.307107	0.000681
Topic 2	Stem diameter	-0.301852	0.000849

Table 4: *Phylum level*. Statistically significant relationships between topics and plant traits based on Spearman's rank correlation coefficient with Holm–Bonferroni correction.
