# Supplementary material for: Latent Dirichlet Allocation modeling of environmental microbiomes
Source: PLoS Comput Biol. 2023 Jun 8;19(6):e1011075. doi: 10.1371/journal.pcbi.1011075 (PMC10249879; doi:10.1371/journal.pcbi.1011075)
Supplement: S5 Table — Consistency of the topics from the different LDA runs. (PDF) [file pcbi.1011075.s020.pdf]

Run 1	Run 2	Run 3	Associated Treatment	Correlated plant traits
T1	T1 (0.99)	T6 (0.99)		
T2	T2 (0.69)	T4 (0.69)	Half water	All runs: leaf mass per area, stem diameter
T3	T5 (0.91)	T1 (0.98)	Generation 0	All runs: Root biomass, stem height, stem diameter
T4	T4 (1)	T3 (0.99)		Run 1 only: stomatal conductance, stem diameter
T5	T3 (0.91)	T5 (0.91)		
T6	T6 (0.8)	T2 (0.8)	Full water treatment	

Table 5: *Phylum level.* Consistency of the topics from the different LDA runs. The numbers in parentheses indicate the cosine similarity value of the corresponding topic from runs 2 or 3 compared to the topic from run 1.
