# Supplementary material for: Latent Dirichlet Allocation modeling of environmental microbiomes
Source: PLoS Comput Biol. 2023 Jun 8;19(6):e1011075. doi: 10.1371/journal.pcbi.1011075 (PMC10249879; doi:10.1371/journal.pcbi.1011075)
Supplement: S6 Table — Effect of using different number of topics at phylum level. (PDF) [file pcbi.1011075.s021.pdf]

	# HW topics	# FW topics	# G0 topics	# topic pairs with cosine similarity ≥ 0.9
3 topics	1	1	0	0
6 topics	1	1	1	0
10 topics	2	2	1	3 (6 unique topics)
20 topics	4	4	5	19 (11 unique topics)

Table 6: *Phylum level.* Effect of using different number of topics at phylum level. Number of topics associated with half-water (HW), full-water (FW), and generation 0 treatments. The last column shows how many topic pairs had cosine similarity of ≥ 0.9. Topics linked to half-water treatment were dominated by *Actinobacteria* and *Bacteroidota*. Topics linked to full-water treatment were dominated by *Verrucomicrobiota*, *Proteobacteria*, and *Bacteroidota*.
