# Supplementary material for: Latent Dirichlet Allocation modeling of environmental microbiomes
Source: PLoS Comput Biol. 2023 Jun 8;19(6):e1011075. doi: 10.1371/journal.pcbi.1011075 (PMC10249879; doi:10.1371/journal.pcbi.1011075)
Supplement: S7 Table — Probability distribution of classes in each LDA topic. (PDF) [file pcbi.1011075.s022.pdf]

	Topic 1	Topic 2	Topic 3	Topic 4	Topic 5	Topic 6	Topic 7	Topic 8
Actinobacteriota_Acidimicrobiia	0.187	0.483	0.628	-	-	-	-	-
Acidobacteriota_Acidobacteriae	-	-	-	-	-	0.094	-	-
Actinobacteriota_Actinobacteria	-	-	-	**62.249**	**19.505**	-	2.733	3.417
Proteobacteria_Alphaproteobacteria	14.516	**22.937**	**25.634**	**25.791**	**10.983**	9.921	**20.079**	**13.828**
Bacteroidota_Bacteroidia	-	4.202	**37.971**	5.232	**18.854**	-	**16.847**	**20.508**
Bacteroidota_NA4	-	-	-	-	-	0.278	1.392	-
Bdellovibrionota_Bdellovibrionia	0.165	-	-	0.484	-	0.689	-	-
Acidobacteriota_Blastocatellia	0.418	0.474	0.320	-	-	-	-	-
Verrucomicrobiota_Chlamydiae	-	-	0.332	-	-	-	0.734	-
Chloroflexi_Chloroflexia	-	-	-	-	-	-	-	**3.980**
Cyanobacteria_Cyanobacteriia	**58.345**	-	-	-	-	0.116	0.768	**7.291**
Deinococcota_Deinococci	-	**54.521**	-	0.477	-	-	-	-
Elusimicrobiota_Elusimicrobia	-	-	0.303	-	-	-	-	-
Proteobacteria_Gammaproteobacteria	**23.754**	**12.564**	**27.730**	-	**21.850**	**83.135**	-	**24.474**
Gemmatimonadota_Gemmatimonadetes	-	-	-	-	-	-	2.369	0.866
Myxococcota_Myxococcia	-	-	-	-	1.074	-	0.714	-
Crenarchaeota_Nitrososphaeria	-	-	-	0.461	-	-	-	-
Nitrospirota_Nitrospiria	-	-	1.443	0.757	-	-	-	-
Bdellovibrionota_Oligoflexia	0.167	-	-	-	-	-	-	-
Planctomycetota_Planctomycetes	-	-	1.140	-	-	-	5.775	1.330
Myxococcota_Polyangia	1.175	0.624	3.118	-	**2.934**	0.176	-	2.075
Patescibacteria_Saccharimonadia	-	-	-	-	**4.759**	-	-	-
Cyanobacteria_Sericytochromatia	-	0.447	-	0.862	-	-	-	-
Actinobacteriota_Thermoleophilia	0.162	-	-	1.551	1.421	0.126	-	-
Cyanobacteria_Vampirivibrionia	-	-	-	-	-	-	-	**18.670**
Verrucomicrobiota_Verrucomicrobiae	-	-	-	0.489	**5.740**	5.008	**46.230**	-
Acidobacteriota_Vicinamibacteria	-	0.769	-	-	-	-	-	-
Planctomycetota_vadinHA49	0.354	0.854	-	-	**5.500**	0.167	-	-

Table 7: *Class level.* Probability distribution of classes in each LDA topic. Only ten most probable classes in each topic are shown. Probabilities were converted to percentages. Effective taxa for each topic highlighted in bold.
