# Supplementary material for: Latent Dirichlet Allocation modeling of environmental microbiomes
Source: PLoS Comput Biol. 2023 Jun 8;19(6):e1011075. doi: 10.1371/journal.pcbi.1011075 (PMC10249879; doi:10.1371/journal.pcbi.1011075)
Supplement: S8 Table — Relative amplifications of classes in each LDA topic. (PDF) [file pcbi.1011075.s023.pdf]

	Topic 1	Topic 2	Topic 3	Topic 4	Topic 5	Topic 6	Topic 7	Topic 8
Actinobacteriota_Acidimicrobiia	1.854	1.967	3.237	-	-	-	-	-
Acidobacteriota_Acidobacteriae	-	-	-	-	-	5.723	-	-
Actinobacteriota_Actinobacteria	-	-	-	8.572	1.350	-	0.385	0.221
Proteobacteria_Alphaproteobacteria	2.078	1.349	1.908	2.356	0.504	3.900	1.876	0.593
Bacteroidota_Bacteroidia	-	0.342	3.915	0.662	1.199	-	2.179	1.218
Bacteroidota_NA4	-	-	-	-	-	7.717	9.170	-
Bdellovibrionota_Bdellovibrionia	1.105	-	-	2.067	-	12.655	-	-
Acidobacteriota_Blastocatellia	6.323	2.945	2.519	-	-	-	-	-
Verrucomicrobiota_Chlamydiae	-	-	1.605	-	-	-	4.455	-
Chloroflexi_Chloroflexia	-	-	-	-	-	-	-	22.995
Cyanobacteria_Cyanobacteriia	21.696	-	-	-	-	0.118	0.186	0.812
Deinococcota_Deinococci	-	12.065	-	0.164	-	-	-	-
Elusimicrobiota_Elusimicrobia	-	-	6.182	-	-	-	-	-
Proteobacteria_Gammaproteobacteria	2.484	0.540	1.508	-	0.733	23.863	-	0.766
Gemmatimonadota_Gemmatimonadetes	-	-	-	-	-	-	9.674	1.623
Myxococcota_Myxococcia	-	-	-	-	3.729	-	5.042	-
Crenarchaeota_Nitrososphaeria	-	-	-	8.719	-	-	-	-
Nitrospirota_Nitrospiria	-	-	5.119	3.295	-	-	-	-
Bdellovibrionota_Oligoflexia	1.908	-	-	-	-	-	-	-
Planctomycetota_Planctomycetes	-	-	1.386	-	-	-	8.807	0.931
Myxococcota_Polyangia	2.540	0.554	3.503	-	2.034	1.044	-	1.343
Patescibacteria_Saccharimonadia	-	-	-	-	6.854	-	-	-
Cyanobacteria_Sericytochromatia	-	2.167	-	6.493	-	-	-	-
Actinobacteriota_Thermoleophilia	0.959	-	-	5.845	2.691	2.047	-	-
Cyanobacteria_Vampirivibrionia	-	-	-	-	-	-	-	24.561
Verrucomicrobiota_Verrucomicrobiae	-	-	-	0.095	0.560	4.185	9.181	-
Acidobacteriota_Vicinamibacteria	-	5.853	-	-	-	-	-	-
Planctomycetota_vadinHA49	1.163	1.151	-	-	5.789	1.508	-	-

Table 8: *Class level*. Relative amplifications of classes in each LDA topic. Only ten most amplified classes in each topic are shown. Amplifications were converted to percentages.
