# Supplementary material for: Latent Dirichlet Allocation modeling of environmental microbiomes
Source: PLoS Comput Biol. 2023 Jun 8;19(6):e1011075. doi: 10.1371/journal.pcbi.1011075 (PMC10249879; doi:10.1371/journal.pcbi.1011075)
Supplement: S9 Table — Statistically significant relationships between topics and plant traits based on Spearman’s rank correlation coefficient with Holm–Bonferroni correction. (PDF) [file pcbi.1011075.s024.pdf]

topic	response	correlation	p-value
Topic 2	Root biomass	0.387509	0.000013
Topic 6	Stem height	0.353059	0.000082
Topic 2	Stem diameter	0.328791	0.000261
Topic 2	Stem height	-0.325995	0.000297
Topic 7	Leaf mass per area	-0.314173	0.000502

Table 9: *Class level.* Statistically significant relationships between topics and plant traits based on Spearman's rank correlation coefficient with Holm–Bonferroni correction.
