# Supplementary material for: Latent Dirichlet Allocation modeling of environmental microbiomes
Source: PLoS Comput Biol. 2023 Jun 8;19(6):e1011075. doi: 10.1371/journal.pcbi.1011075 (PMC10249879; doi:10.1371/journal.pcbi.1011075)
Supplement: S10 Table — Probability distribution of orders in each LDA topic. (PDF) [file pcbi.1011075.s025.pdf]

	Topic 1	Topic 2	Topic 3	Topic 4	Topic 5	Topic 6	Topic 7	Topic 8	Topic 9	Topic 10
Proteobacteria_Alphaproteobacteria_Azospirillales	-	**10.521**	-	-	-	-	-	-	-	-
Bacteroidota_nan_NA20	-	-	-	1.483	-	-	-	-	-	-
Proteobacteria_Gammaproteobacteria_Burkholderiales	**14.035**	**15.897**	**16.932**	**18.544**	**60.443**	-	**16.292**	**13.296**	**7.736**	5.392
Proteobacteria_Alphaproteobacteria_Caulobacterales	-	-	3.892	-	-	-	-	-	3.134	2.038
Proteobacteria_Gammaproteobacteria_Cellvibrionales	**3.390**	-	-	-	-	-	-	-	-	-
Bacteroidota_Bacteroidia_Chitinophagales	3.447	**16.172**	-	1.543	1.904	-	**4.368**	**5.044**	-	-
Cyanobacteria_Cyanobacteriia_Chloroplast	**32.533**	-	1.054	-	-	-	-	-	-	-
Verrucomicrobiota_Verrucomicrobiae_Chthoniobacterales	**8.650**	**5.231**	-	-	-	**3.443**	-	-	-	-
Actinobacteriota_Actinobacteria_Corynebacteriales	-	**1.471**	-	-	-	-	-	-	-	-
Cyanobacteria_Cyanobacteriia_NA31	-	-	-	-	-	-	-	-	**41.731**	-
Bacteroidota_Bacteroidia_Cytophagales	-	**5.316**	-	-	1.128	1.747	**77.694**	**12.688**	3.225	**6.375**
Deinococcota_Deinococci_Deinococcales	-	-	**45.102**	-	-	-	-	-	-	-
Proteobacteria_Gammaproteobacteria_Diplorickettsiales	-	-	-	-	-	-	-	-	-	**7.170**
Bacteroidota_Bacteroidia_Flavobacteriales	-	-	-	2.413	-	-	-	**6.805**	-	-
Actinobacteriota_Actinobacteria_Frankiales	-	-	-	-	-	2.679	-	-	-	-
Actinobacteriota_Actinobacteria_Micrococcales	-	**7.038**	**11.245**	2.945	-	**36.016**	**4.020**	-	1.818	-
Verrucomicrobiota_Verrucomicrobiae_Opitutales	-	-	-	-	-	-	**3.882**	-	-	-
Planctomycetota_Planctomycetes_Planctomycetales	2.554	-	-	-	-	-	-	-	-	**6.635**
Planctomycetota_vadinHA49_NA63	-	-	-	-	0.799	-	-	-	2.591	-
Myxococcota_Polyangia_Polyangiales	-	-	-	-	0.977	-	-	-	-	-
Actinobacteriota_Actinobacteria_Propionibacteriales	-	-	0.857	-	-	**5.746**	-	-	**3.240**	-
Proteobacteria_Gammaproteobacteria_Pseudomonadales	-	**7.320**	-	-	-	-	-	-	-	-
Actinobacteriota_Actinobacteria_Pseudonocardiales	-	-	-	-	2.215	-	-	-	-	-
Proteobacteria_Alphaproteobacteria_Rhizobiales	**10.573**	**10.782**	**10.009**	**7.640**	**8.517**	**18.027**	**11.747**	**2.729**	**10.359**	**6.41**
Proteobacteria_Alphaproteobacteria_Rhodobacterales	-	-	3.571	-	-	-	-	**8.402**	-	2.289
Proteobacteria_Alphaproteobacteria_Rickettsiales	-	-	-	1.817	1.523	-	-	-	-	-
Patescibacteria_Saccharimonadia_Saccharimonadales	-	-	-	-	-	2.757	-	-	-	-
Proteobacteria_Gammaproteobacteria_Salinisphaerales	**3.967**	-	1.496	-	-	2.152	-	**6.551**	-	-
Bacteroidota_Bacteroidia_Sphingobacteriales	**4.894**	**2.008**	-	**3.207**	**9.487**	**11.416**	**6.272**	**6.408**	2.814	3.853
Proteobacteria_Alphaproteobacteria_Sphingomonadales	2.437	-	0.743	2.839	4.684	3.331	**3.388**	**9.683**	**6.329**	5.023
Actinobacteriota_Actinobacteria_Streptomycetales	-	-	-	-	-	-	**18.839**	-	-	-
Cyanobacteria_Vampirivibrionia_Vampirovibrionales	-	-	-	-	-	-	**7.052**	-	-	-
Verrucomicrobiota_Verrucomicrobiae_Verrucomicrobiales	-	-	-	**46.979**	-	-	-	**7.414**	-	-
Proteobacteria_Gammaproteobacteria_Xanthomonadales	-	-	-	-	-	-	-	-	-	**40.353**

Table 10: *Order level.* Probability distribution of orders in each LDA topic. Only ten most probable orders in each topic are shown. Probabilities were converted to percentages. Effective taxa for each topic highlighted in bold.
