# Supplementary material for: Latent Dirichlet Allocation modeling of environmental microbiomes
Source: PLoS Comput Biol. 2023 Jun 8;19(6):e1011075. doi: 10.1371/journal.pcbi.1011075 (PMC10249879; doi:10.1371/journal.pcbi.1011075)
Supplement: S11 Table — Relative amplifications of orders in each LDA topic. (PDF) [file pcbi.1011075.s026.pdf]

	Topic 1	Topic 2	Topic 3	Topic 4	Topic 5	Topic 6	Topic 7	Topic 8	Topic 9	Topic 10
Proteobacteria_Alphaproteobacteria_Azospirillales	-	4.359	-	-	-	-	-	-	-	-
Bacteroidota_nan_NA20	-	-	-	6.475	-	-	-	-	-	-
Proteobacteria_Gammaproteobacteria_Burkholderiales	0.373	0.212	0.662	1.027	4.591	-	0.595	0.352	0.230	0.154
Proteobacteria_Alphaproteobacteria_Caulobacterales	-	-	2.428	-	-	-	-	-	1.485	0.929
Proteobacteria_Gammaproteobacteria_Cellvibrionales	3.129	-	-	-	-	-	-	-	-	-
Bacteroidota_Bacteroidia_Chitinophagales	0.635	1.496	-	0.593	1.004	-	1.107	0.926	-	-
Cyanobacteria_Cyanobacteriia_Chloroplast	6.226	-	0.297	-	-	-	-	-	-	-
Verrucomicrobiota_Verrucomicrobiae_Chthoniobacterales	2.631	0.799	-	-	-	1.341	-	-	-	-
Actinobacteriota_Actinobacteria_Corynebacteriales	-	1.831	-	-	-	-	-	-	-	-
Cyanobacteria_Cyanobacteriia_NA31	-	-	-	-	-	-	-	-	7.705	-
Bacteroidota_Bacteroidia_Cytophagales	-	0.373	-	-	0.451	0.313	1.478	1.766	0.504	0.958
Deinococcota_Deinococci_Deinococcales	-	-	6.704	-	-	-	-	-	-	-
Proteobacteria_Gammaproteobacteria_Diplorickettsiales	-	-	-	-	-	-	-	-	-	7.740
Bacteroidota_Bacteroidia_Flavobacteriales	-	-	-	2.057	-	-	-	2.771	-	-
Actinobacteriota_Actinobacteria_Frankiales	-	-	-	-	-	2.336	-	-	-	-
Actinobacteriota_Actinobacteria_Micrococcales	-	0.218	1.021	0.379	-	2.844	0.341	-	0.125	-
Verrucomicrobiota_Verrucomicrobiae_Opitutales	-	-	-	-	-	-	4.790	-	-	-
Planctomycetota_Planctomycetes_Planctomycetales	1.589	-	-	-	-	-	-	-	-	4.446
Planctomycetota_vadinHA49_NA63	-	-	-	-	2.847	-	-	-	3.608	-
Myxococcota_Polyangia_Polyangiales	-	-	-	-	2.855	-	-	-	-	-
Actinobacteriota_Actinobacteria_Propionibacteriales	-	-	0.449	-	-	2.615	-	-	1.288	-
Proteobacteria_Gammaproteobacteria_Pseudomonadales	-	4.277	-	-	-	-	-	-	-	-
Actinobacteriota_Actinobacteria_Pseudonocardiales	-	-	-	-	5.848	-	-	-	-	-
Proteobacteria_Alphaproteobacteria_Rhizobiales	0.548	0.281	0.765	0.826	1.264	1.197	0.838	0.141	0.601	0.358
Proteobacteria_Alphaproteobacteria_Rhodobacterales	-	-	1.500	-	-	-	-	2.388	-	0.703
Proteobacteria_Alphaproteobacteria_Rickettsiales	-	-	-	2.993	3.443	-	-	-	-	-
Patescibacteria_Saccharimonadia_Saccharimonadales	-	-	-	-	-	2.977	-	-	-	-
Proteobacteria_Gammaproteobacteria_Salinisphaerales	1.060	-	0.589	-	-	0.736	-	1.744	-	-
Bacteroidota_Bacteroidia_Sphingobacteriales	0.422	0.087	-	0.577	2.341	1.261	0.744	0.551	0.271	0.358
Proteobacteria_Alphaproteobacteria_Sphingomonadales	0.298	-	0.134	0.725	1.642	0.522	0.571	1.182	0.867	0.663
Actinobacteriota_Actinobacteria_Streptomycetales	-	-	-	-	-	-	9.891	-	-	-
Cyanobacteria_Vampirivibrionia_Vampirovibrionales	-	-	-	-	-	-	8.906	-	-	-
Verrucomicrobiota_Verrucomicrobiae_Verrucomicrobiales	-	-	-	8.290	-	-	-	0.625	-	-
Proteobacteria_Gammaproteobacteria_Xanthomonadales	-	-	-	-	-	-	-	-	-	8.002

Table 11: *Order level.* Relative amplifications of orders in each LDA topic. Only ten most amplified orders in each topic are shown. Amplifications were converted to percentages.
