# Supplementary material for: Latent Dirichlet Allocation modeling of environmental microbiomes
Source: PLoS Comput Biol. 2023 Jun 8;19(6):e1011075. doi: 10.1371/journal.pcbi.1011075 (PMC10249879; doi:10.1371/journal.pcbi.1011075)
Supplement: S12 Table — Statistically significant relationships between topics and plant traits based on Spearman’s rank correlation coefficient with Holm–Bonferroni correction. (PDF) [file pcbi.1011075.s027.pdf]

topic	response	correlation	p-value
Topic 9	Stem height	0.467979	8.025462e-08
Topic 2	Stem height	-0.442553	4.668667e-07
Topic 3	Root biomass	0.399039	6.949396e-06
Topic 4	Leaf mass per area	-0.377003	2.376296e-05
Topic 6	Leaf mass per area	0.361012	5.495218e-05
Topic 9	Root biomass	-0.354309	7.708443e-05
Topic 3	Stem height	-0.352856	8.286636e-05
Topic 3	Stem diameter	0.341337	1.452856e-04
Topic 8	Stem height	-0.330276	2.441251e-04
Topic 2	Root biomass	0.319484	3.976095e-04

Table 12: *Order level.* Statistically significant relationships between topics and plant traits based on Spearman's rank correlation coefficient with Holm–Bonferroni correction.
