# Supplementary material for: Latent Dirichlet Allocation modeling of environmental microbiomes
Source: PLoS Comput Biol. 2023 Jun 8;19(6):e1011075. doi: 10.1371/journal.pcbi.1011075 (PMC10249879; doi:10.1371/journal.pcbi.1011075)
Supplement: S13 Table — Probability distribution of families in each LDA topic. (PDF) [file pcbi.1011075.s028.pdf]

	Topic 1	Topic 2	Topic 3	Topic 4	Topic 5	Topic 6	Topic 7	Topic 8	Topic 9	Topic 10	Topic 11	Topic 12	Topic 13	Topic 14	Topic 15	Topic 16	Topic 17	Topic 18	Topic 19	Topic 20
Proteobacteria_Alphaproteobacteria_Azospirillales_Azospirillaceae	-	-	-	-	-	-	-	11.175	-	-	-	-	-	-	-	-	-	-	-	-
Proteobacteria_Alphaproteobacteria_Rhizobiales_Beijerinckiaceae	3.206	-	25.638	-	3.880	-	-	-	-	11.526	-	-	-	-	-	-	-	-	3.123	-
Proteobacteria_Gammaproteobacteria_Burkholderiales_Burkholderiaceae	-	-	-	-	-	-	-	-	-	-	45.942	-	-	-	-	-	-	-	-	-
Proteobacteria_Alphaproteobacteria_Caulobacterales_Caulobacteraceae	-	-	-	-	-	-	-	-	-	-	-	-	-	-	-	-	-	-	4.555	-
Bacteroidota_Bacteroidia_Chitinophagales_Chitinophagaceae	-	-	-	7.916	-	-	-	17.759	-	-	-	-	-	-	-	-	-	-	-	-
Verrucomicrobiota_Verrucomicrobiae_Chthoniobacterales_Chthoniobacteraceae	-	-	-	-	-	-	-	-	-	-	-	-	-	-	-	-	-	10.509	-	-
Proteobacteria_Gammaproteobacteria_Burkholderiales_Comamonadaceae	-	-	10.281	12.943	7.893	4.855	12.288	-	12.364	-	-	9.650	-	10.800	-	12.280	9.918	17.228	5.768	-
Bacteroidota_Bacteroidia_Flavobacteriales_Crocinitomicaceae	-	-	-	-	-	-	-	-	-	-	-	-	-	-	-	-	7.417	-	-	-
Cyanobacteria_Cyanobacteriia_Chloroplast_NA67	-	-	-	-	-	-	-	-	-	-	-	-	-	11.417	-	-	-	-	-	20.682
Cyanobacteria_Cyanobacteriia_nan_NA69	-	-	-	-	46.094	-	-	-	-	-	-	-	-	-	-	-	-	-	-	-
Deinococcota_Deinococci_Deinococcales_Deinococcaceae	3.369	-	-	-	-	-	-	-	-	-	-	-	-	-	-	-	-	-	70.861	-
Proteobacteria_Gammaproteobacteria_Diplorickettsiales_Diplorickettsiaceae	-	-	-	-	-	-	-	-	-	-	-	-	19.514	-	-	-	-	-	-	-
Proteobacteria_Gammaproteobacteria_Burkholderiales_Gallionellaceae	-	-	-	-	-	-	-	-	-	10.321	-	-	-	-	-	-	-	-	-	-
Bacteroidota_Bacteroidia_Sphingobacteriales_KD3-93	-	-	-	-	-	-	-	-	-	-	-	-	-	-	-	7.035	-	-	-	-
Actinobacteriota_Actinobacteria_Micrococcales_Micrococcaceae	50.769	18.685	19.510	-	-	8.278	-	9.026	-	9.918	7.248	-	-	-	9.065	-	-	-	-	-
Bacteroidota_Bacteroidia_Cytophagales_Microscillaceae	-	-	-	13.747	-	-	-	-	6.450	-	-	-	-	-	4.391	-	-	-	-	-
Actinobacteriota_Actinobacteria_Propionibacteriales_Nocardioidaceae	-	-	-	-	-	-	-	-	-	11.395	-	-	-	-	-	-	-	-	-	-
Verrucomicrobiota_Verrucomicrobiae_Opitutales_Opitutaceae	-	-	-	-	-	-	-	-	5.313	-	-	-	-	-	-	-	-	-	-	-
Proteobacteria_Gammaproteobacteria_Burkholderiales_Oxalobacteraceae	-	-	17.247	-	-	10.424	-	-	11.501	8.617	-	18.338	-	-	-	12.963	3.997	8.184	6.132	-
Proteobacteria_Gammaproteobacteria_Burkholderiales_NA143	-	-	-	-	-	-	-	-	-	-	-	-	-	28.711	-	-	-	-	-	-
Proteobacteria_Gammaproteobacteria_Pseudomonadales_Pseudomonadaceae	-	-	-	-	-	-	-	9.310	-	-	-	-	-	-	-	-	-	-	-	-
Actinobacteriota_Actinobacteria_Pseudonocardiales_Pseudonocardiaceae	-	-	-	-	-	-	-	-	-	-	2.497	-	-	-	-	-	-	-	-	-
Proteobacteria_Alphaproteobacteria_Rhizobiales_Rhizobiaceae	-	4.432	-	-	9.429	-	-	6.228	-	-	-	-	12.662	11.595	-	-	-	7.708	-	4.408
Proteobacteria_Gammaproteobacteria_Xanthomonadales_Rhodanobacteraceae	-	-	-	-	-	-	-	-	-	-	-	-	-	-	-	-	-	-	-	3.697
Proteobacteria_Alphaproteobacteria_Rhodobacterales_Rhodobacteraceae	-	19.483	-	-	-	-	3.392	-	-	-	-	-	-	-	-	-	-	-	-	-
Planctomycetota_Planctomycetes_Planctomycetales_Rubinisphaeraceae	-	-	-	-	-	-	-	-	-	-	-	-	11.328	5.873	-	-	-	-	-	-
Verrucomicrobiota_Verrucomicrobiae_Verrucomicrobiales_Rubritaleaceae	-	-	-	-	-	-	-	-	-	-	-	-	-	-	-	-	-	-	-	38.101
Proteobacteria_Gammaproteobacteria_Salinisphaerales_Solimonadaceae	-	-	-	-	-	-	-	-	-	-	-	-	-	-	6.527	10.122	-	-	-	-
Bacteroidota_Bacteroidia_Sphingobacteriales_Sphingobacteriaceae	-	-	4.725	-	-	-	-	-	-	-	-	16.413	-	-	-	-	-	-	-	-
Proteobacteria_Alphaproteobacteria_Sphingomonadales_Sphingomonadaceae	4.510	12.120	-	7.183	5.245	4.125	3.983	-	-	-	4.111	7.151	-	-	-	-	4.981	5.901	-	3.578
Bacteroidota_Bacteroidia_Cytophagales_Spirosomaceae	-	6.505	-	-	-	-	-	-	-	-	2.342	9.760	5.225	-	-	-	-	-	-	-
Actinobacteriota_Actinobacteria_Streptomycetales_Streptomycetaceae	-	-	-	-	-	-	-	-	27.370	-	-	-	-	-	-	-	-	-	-	-
Cyanobacteria_Vampirivibrionia_Vampirovibrionales_Vampirovibrionaceae	-	-	-	-	-	-	-	-	-	-	-	-	-	-	37.545	-	-	-	-	-
Verrucomicrobiota_Verrucomicrobiae_Verrucomicrobiales_Verrucomicrobiaceae	-	-	-	-	-	-	6.990	-	-	-	-	-	-	-	-	-	43.843	-	-	-
Verrucomicrobiota_Verrucomicrobiae_Verrucomicrobiales_NA170	-	-	-	-	-	37.286	-	-	-	-	-	-	-	-	-	-	-	-	-	-
Proteobacteria_Gammaproteobacteria_Xanthomonadales_Xanthomonadaceae	-	-	-	-	-	-	50.018	-	-	-	-	-	-	-	-	-	-	-	-	-
Bacteroidota_Bacteroidia_Sphingobacteriales_env.OPS_17	10.488	-	-	7.926	-	-	-	-	-	-	-	-	4.842	-	4.433	8.256	-	-	-	-

Table 13: *Family level.* Probability distribution of families in each LDA topic. Only five most probable families in each topic are shown. Probabilities were converted to percentages.
