# Supplementary material for: Latent Dirichlet Allocation modeling of environmental microbiomes
Source: PLoS Comput Biol. 2023 Jun 8;19(6):e1011075. doi: 10.1371/journal.pcbi.1011075 (PMC10249879; doi:10.1371/journal.pcbi.1011075)
Supplement: S15 Table — Statistically significant relationships between topics and plant traits based on Spearman’s rank correlation coefficient with Holm–Bonferroni correction. (PDF) [file pcbi.1011075.s030.pdf]

topic	response	correlation	p-value
Topic 11	Root biomass	-0.449708	2.885511e-07
Topic 11	Stem diameter	-0.427794	1.217349e-06
Topic 8	Stem height	-0.413754	2.907603e-06
Topic 10	Stem diameter	-0.405591	4.738668e-06
Topic 4	Stem height	-0.382249	1.787429e-05
Topic 19	Root biomass	0.376841	2.397130e-05
Topic 8	Root biomass	0.359336	5.984535e-05
Topic 12	Stem diameter	-0.354869	7.495701e-05
Topic 5	Stem height	0.351624	8.808690e-05
Topic 14	Stem height	0.350421	9.348150e-05
Topic 11	% Leaf water content	-0.348288	1.038044e-04
Topic 12	Stem height	0.341133	1.467047e-04
Topic 17	% Leaf water content	0.338669	1.649512e-04
Topic 16	Root biomass	0.337089	1.777392e-04
Topic 10	% Leaf water content	-0.333605	2.092444e-04
Topic 14	Leaf mass per area	-0.330991	2.362055e-04
Topic 10	Root biomass	-0.330088	2.462383e-04

Table 15: *Family level.* Statistically significant relationships between topics and plant traits based on Spearman's rank correlation coefficient with Holm–Bonferroni correction.
