# Supplementary material for: Latent Dirichlet Allocation modeling of environmental microbiomes
Source: PLoS Comput Biol. 2023 Jun 8;19(6):e1011075. doi: 10.1371/journal.pcbi.1011075 (PMC10249879; doi:10.1371/journal.pcbi.1011075)
Supplement: S16 Table — Probability distribution of ASVs in each LDA topic. (PDF) [file pcbi.1011075.s031.pdf]

	Topic 1	Topic 2	Topic 3	Topic 4	Topic 5	Topic 6	Topic 7	Topic 8	Topic 9	Topic 10	Topic 11	Topic 12	Topic 13	Topic 14	Topic 15	Topic 16	Topic 17	Topic 18	Topic 19	Topic 20	Topic 21	Topic 22	Topic 23	Topic 24	Topic 25
Planctomycetota__Planctomycetes__Planctomycetales__Rubinisphaeraceae__SH-PL14__nan_	-	-	-	5.370	-	-	-	-	-	-	-	-	-	-	-	-	-	-	-	-	-	-	-	-	-
Verrucomicrobiota__Verrucomicrobiae__Verrucomicrobiales__Verrucomicrobiaceae__Verrucomicrobium__spinosum_	-	-	-	3.359	-	-	-	-	-	-	-	-	-	-	-	-	-	-	-	-	4.602	-	-	-	-
Verrucomicrobiota__Verrucomicrobiae__Chthoniobacterales__Chthoniobacteraceae__Chthoniobacter__nan_	-	-	-	-	-	-	-	-	-	-	-	-	-	-	-	-	-	-	-	2.937	-	-	-	-	-
Verrucomicrobiota__Verrucomicrobiae__Chthoniobacterales__Chthoniobacteraceae__Chthoniobacter__nan_	-	-	-	-	-	2.732	-	-	-	-	-	-	-	-	-	-	-	-	-	-	-	-	3.514	-	-
Verrucomicrobiota__Verrucomicrobiae__Chthoniobacterales__Chthoniobacteraceae__Chthoniobacter__nan_	-	-	-	-	-	-	-	-	-	-	-	-	-	-	-	-	2.580	-	-	-	-	-	-	-	-
Verrucomicrobiota__Verrucomicrobiae__Verrucomicrobiales__Verrucomicrobiaceae__nan__nan_	-	2.998	-	6.929	-	-	6.368	-	-	-	-	-	-	4.046	-	13.947	-	-	5.543	-	-	6.766	14.866	-	-
Verrucomicrobiota__Verrucomicrobiae__Verrucomicrobiales__Rubritaleaceae__nan__nan_	-	17.051	-	-	-	-	-	-	-	-	-	-	-	-	-	-	-	-	-	-	8.874	-	-	-	-
Verrucomicrobiota__Verrucomicrobiae__Verrucomicrobiales__nan__nan__nan_	-	-	-	-	-	-	-	34.553	-	-	-	-	-	-	-	-	-	4.726	-	-	-	-	-	-	-
Cyanobacteria__Vampirivibrionia__Vampirovibrionales__Vampirovibrionaceae__Vampirovibrio__nan_	7.156	-	-	-	-	-	-	-	-	-	-	-	-	-	-	-	-	-	5.336	-	-	-	-	-	-
Cyanobacteria__Cyanobacteriia__Chloroplast__nan__nan__	-	-	-	-	-	-	-	-	-	-	-	-	-	-	-	-	-	-	-	-	21.224	-	-	-	-
Verrucomicrobiota__Verrucomicrobiae__Opitutales__Opitutaceae__nan__nan_	-	-	-	-	-	-	-	-	-	-	-	16.174	-	-	-	-	-	-	-	-	-	-	-	-	-
Cyanobacteria__Cyanobacteriia__Chloroplast__nan__nan__nan_	-	-	-	-	-	3.521	-	-	-	-	-	-	-	-	-	-	-	-	-	-	-	-	-	-	-
Deinococcota__Deinococci__Deinococcales__Deinococcaceae__Deinococcus__nan_	-	-	-	-	-	-	-	-	-	16.770	-	-	-	-	-	-	-	-	-	-	-	-	-	-	-
Deinococcota__Deinococci__Deinococcales__Deinococcaceae__Deinococcus__nan_	-	-	-	-	-	-	2.553	-	-	46.888	-	-	-	-	-	-	-	5.695	-	-	-	-	-	-	-
Cyanobacteria__Cyanobacteriia__nan__nan__nan__nan__	-	5.987	-	-	-	-	-	5.252	-	3.494	-	-	-	-	5.147	-	-	6.591	-	-	-	-	-	5.743	48.326
Proteobacteria__Gammaproteobacteria__Diplorickettsiales__Diplorickettsiaceae__Aquicella__nan_	-	-	-	-	-	-	-	-	-	-	-	-	-	-	-	-	-	-	7.205	-	-	-	-	-	-
Actinobacteriota__Actinobacteria__Streptomycetales__Streptomycetaceae__Streptomyces__nan_	-	-	16.197	-	-	-	-	-	-	-	-	18.158	-	-	5.459	-	-	-	-	-	-	-	-	-	-
Actinobacteriota__Actinobacteria__Pseudonocardiales__Pseudonocardiaceae__Pseudonocardia__nan__	-	-	-	-	-	-	-	-	-	-	1.272	-	-	-	-	-	-	-	-	-	-	-	-	-	-
Actinobacteriota__Actinobacteria__Micrococcales__Micrococcaceae__Pseudarthrobacter__nan_	4.691	-	5.593	-	5.493	-	5.376	11.169	12.532	18.650	5.646	6.105	11.714	-	3.482	-	18.116	10.473	-	11.892	-	-	3.771	7.934	4.335
Bacteroidota__Bacteroidia__Cytophagales__Microscillaceae__nan__nan_	2.697	-	4.382	-	-	-	-	-	-	-	-	3.272	-	-	-	-	-	-	-	-	-	-	-	3.085	-
Bacteroidota__Bacteroidia__Sphingobacteriales__env.OPS_17__nan__nan_	-	-	-	-	-	-	-	-	-	-	-	-	-	-	-	-	-	-	-	-	-	-	-	5.809	-
Bacteroidota__Bacteroidia__Chitinophagales__Chitinophagaceae__Niastella__vici_	-	-	3.678	-	-	-	-	-	-	-	-	-	-	-	-	-	-	-	-	-	-	-	-	-	-
Bacteroidota__Bacteroidia__Sphingobacteriales__env.OPS_17__nan__nan_	-	-	-	-	-	3.536	-	-	-	-	-	-	3.934	-	-	-	-	-	-	4.123	-	-	-	-	-
Proteobacteria__Alphaproteobacteria__Sphingomonadales__Sphingomonadaceae__Sphingobium__nan_	-	-	-	-	-	-	-	-	-	-	-	-	-	-	-	-	-	-	-	-	-	-	-	2.588	-
Proteobacteria__Alphaproteobacteria__Sphingomonadales__Sphingomonadaceae__Sphingopyxis__nan_	-	-	-	-	-	2.514	-	-	-	-	-	-	-	-	-	-	-	-	-	-	-	4.211	-	-	-
Proteobacteria__Alphaproteobacteria__Rhodobacterales__Rhodobacteraceae__Rhodobacter__nan_	-	-	-	-	-	-	7.454	-	-	2.394	-	-	-	3.611	-	-	-	-	-	-	-	5.428	-	-	-
Proteobacteria__Alphaproteobacteria__Rhizobiales__Rhizobiaceae__nan__nan_	-	-	-	3.477	-	-	-	-	-	-	1.496	-	-	-	4.143	-	-	-	-	-	-	-	-	-	6.121
Proteobacteria__Gammaproteobacteria__Pseudomonadales__Pseudomonadaceae__Pseudomonas__nan_	-	-	-	-	-	-	-	-	-	-	-	-	-	-	-	-	4.646	-	-	-	-	-	-	-	-
Proteobacteria__Gammaproteobacteria__Pseudomonadales__Pseudomonadaceae__Pseudomonas__nan_	-	-	-	-	-	-	-	-	-	-	-	-	-	-	-	-	2.951	-	-	-	-	-	-	-	-
Proteobacteria__Alphaproteobacteria__Rhizobiales__Beijerinckiaceae__Methylobacterium-Methylorubrum__nan_	-	-	-	-	8.781	-	-	-	7.711	-	-	-	5.868	-	-	-	-	-	-	3.547	-	-	-	-	2.878
Proteobacteria__Alphaproteobacteria__Rhizobiales__Beijerinckiaceae__Methylobacterium-Methylorubrum__nan_	-	-	-	-	-	-	-	-	2.224	-	-	-	-	-	-	-	-	-	-	-	-	-	-	-	-
Proteobacteria__Alphaproteobacteria__Azospirillales__Azospirillaceae__Azospirillum__nan_	-	-	-	-	5.280	-	-	-	-	-	-	-	-	-	-	-	-	-	-	-	-	-	-	-	-
Bacteroidota__Bacteroidia__Flavobacteriales__Crocinitomicaceae__Fluviicola__nan_	-	-	-	-	-	-	-	-	-	-	-	-	-	2.487	-	5.582	-	-	-	-	-	-	-	-	-
Proteobacteria__Gammaproteobacteria__Burkholderiales__Oxalobacteraceae__Massilia__aerilata/phosphatilytica_	-	-	-	-	-	-	-	-	-	-	1.660	-	-	-	-	-	-	3.826	-	-	5.532	-	-	-	-
Proteobacteria__Gammaproteobacteria__Burkholderiales__Comamonadaceae__nan__nan_	-	-	7.616	-	-	-	-	-	-	-	-	-	-	-	7.325	-	-	-	-	-	-	-	-	-	-
Proteobacteria__Gammaproteobacteria__Burkholderiales__Comamonadaceae__nan__nan_	-	11.072	-	-	-	3.552	2.769	3.345	-	-	-	-	-	-	-	10.610	-	-	4.045	-	3.523	8.424	6.679	-	2.756
Proteobacteria__Gammaproteobacteria__Burkholderiales__Oxalobacteraceae__Massilia__nan_	-	-	-	-	6.925	-	-	8.900	-	-	-	-	2.838	-	-	9.507	3.288	-	-	3.362	-	-	-	-	-
Proteobacteria__Gammaproteobacteria__Burkholderiales__Oxalobacteraceae__Herbaspirillum__autotrophicum/hiltneri/lusitanum_	-	-	-	-	-	-	-	-	-	-	-	19.186	-	-	-	-	-	-	-	-	-	-	-	-	-
Proteobacteria__Gammaproteobacteria__Burkholderiales__Burkholderiaceae__Ralstonia__nan_	-	-	-	-	-	-	-	-	-	-	49.054	-	-	-	-	-	-	-	-	-	-	-	-	-	-
Proteobacteria__Gammaproteobacteria__Burkholderiales__Oxalobacteraceae__Noviherbaspirillum__suwonense_	-	-	-	-	-	-	-	-	2.796	-	-	-	-	-	-	-	-	-	-	-	-	-	-	-	-
Proteobacteria__Gammaproteobacteria__Burkholderiales__nan__nan__nan_	-	-	-	3.802	-	-	-	-	-	-	-	-	-	5.291	-	4.147	-	-	14.862	-	-	-	-	-	-
Proteobacteria__Gammaproteobacteria__Burkholderiales__Oxalobacteraceae__Pseudoduganella__eburnea_	4.716	-	-	-	-	-	-	-	2.886	-	-	-	-	-	-	-	-	-	-	-	-	-	-	-	-
Proteobacteria__Gammaproteobacteria__Xanthomonadales__Xanthomonadaceae__Luteimonas__nan_	-	-	-	-	-	-	-	-	-	-	-	-	-	-	-	-	-	-	-	-	-	28.677	-	-	-
Proteobacteria__Gammaproteobacteria__Xanthomonadales__Xanthomonadaceae__Luteimonas__nan_	-	12.859	-	-	-	-	-	-	-	-	-	-	-	-	-	-	-	-	-	-	-	-	-	-	-
Bacteroidota__Bacteroidia__Cytophagales__Spirosomaceae__Dyadobacter__endophyticus/fermentans_	3.690	-	-	-	7.266	-	-	-	-	-	-	-	-	-	-	-	-	-	-	-	-	-	-	-	-
Bacteroidota__Bacteroidia__Cytophagales__Spirosomaceae__Dyadobacter__nan_	-	-	-	-	-	-	-	-	-	-	-	-	-	-	-	-	-	-	-	-	-	-	4.013	-	-
Cyanobacteria__Cyanobacteriia__nan__nan__nan__nan__	-	-	-	-	-	-	-	-	-	-	-	-	4.866	-	-	-	-	-	-	-	-	-	-	-	-
Cyanobacteria__Cyanobacteriia__Chloroplast__nan__nan__nan__	-	-	-	-	-	-	-	-	-	-	-	-	-	3.435	-	-	-	-	-	-	-	-	-	-	-

Table 16: *ASV level*. Probability distribution of ASVs in each LDA topic. Only five most probable ASVs in each topic are shown. Probabilities were converted to percentages.
