# Supplementary material for: Latent Dirichlet Allocation modeling of environmental microbiomes
Source: PLoS Comput Biol. 2023 Jun 8;19(6):e1011075. doi: 10.1371/journal.pcbi.1011075 (PMC10249879; doi:10.1371/journal.pcbi.1011075)
Supplement: S17 Table — Relative amplifications of ASVs in each LDA topic. (PDF) [file pcbi.1011075.s032.pdf]

	Topic 1	Topic 2	Topic 3	Topic 4	Topic 5	Topic 6	Topic 7	Topic 8	Topic 9	Topic 10	Topic 11	Topic 12	Topic 13	Topic 14	Topic 15	Topic 16	Topic 17	Topic 18	Topic 19	Topic 20	Topic 21	Topic 22	Topic 23	Topic 24	Topic 25
Cyanobacteria__Cyanobacteriia__Cyanobacteriales__Coleofasciculaceae__Symplocastrum_CPER-KK1__nan_	-	-	-	-	-	-	-	-	-	-	-	-	-	-	-	-	-	3.556	-	-	-	-	-	-	-
Proteobacteria__Gammaproteobacteria__Burkholderiales__Burkholderiaceae__Ralstonia__nan_	-	-	-	-	-	-	-	-	-	-	1.021	-	-	-	-	-	-	-	-	-	-	-	-	-	-
Planctomycetota__Planctomycetes__Planctomycetales__Schlesneriaceae__Planctopirus__nan_	-	-	-	-	-	-	-	-	-	-	-	-	-	-	-	1.594	-	-	-	-	-	-	-	-	-
Patescibacteria__Parcubacteria__Candidatus_Kaiserbacteria__nan__nan__nan_	-	-	-	-	-	-	-	-	-	-	-	-	-	-	-	-	-	-	-	-	-	2.911	-	-	-
Chloroflexi__Chloroflexia__Thermomicrobiales__JG30-KF-CM45__nan__nan_	-	-	-	-	-	-	-	-	-	-	-	-	-	-	-	1.586	-	-	-	-	-	-	-	-	-
Chloroflexi__Anaerolineae__SBR1031__A4b__nan__nan_	-	-	-	0.886	-	-	-	-	-	-	-	-	-	-	-	-	-	-	-	-	-	-	-	-	-
Chloroflexi__Ktedonobacteria__C0119__nan__nan__nan_	1.053	-	-	-	-	-	-	-	-	-	-	-	-	-	-	-	-	-	-	-	-	-	-	-	-
Planctomycetota__Planctomycetes__Isosphaerales__Isosphaeraceae__nan__nan__	-	-	-	-	-	-	-	-	-	-	-	-	-	-	-	-	1.029	-	-	-	-	-	-	-	-
Planctomycetota__Phycisphaerae__Tepidisphaerales__WD2101_soil_group__nan__nan_	-	-	-	0.886	-	-	-	-	-	-	-	-	-	-	-	-	-	-	-	-	-	-	-	-	-
Planctomycetota__Planctomycetes__Pirellulales__Pirellulaceae__Pirellula__nan_	-	-	-	-	-	-	-	-	-	-	-	-	0.628	-	-	-	-	-	-	-	-	-	-	-	-
Acidobacteriota__Holophagae__Subgroup_7__nan__nan__nan_	-	-	-	-	-	-	-	-	0.429	-	-	-	-	-	-	-	-	-	-	-	-	-	-	-	-
Patescibacteria__Saccharimonadia__Saccharimonadales__LWQ8__nan__nan__	-	-	-	-	-	-	-	-	0.485	-	-	-	-	-	-	-	-	-	-	-	-	-	-	-	-
Chloroflexi__Anaerolineae__RBG-13-54-9__nan__nan__nan_	-	-	-	-	-	-	0.440	-	-	-	-	-	-	-	-	-	-	-	-	-	-	-	-	-	-
Chloroflexi__Chloroflexia__Chloroflexales__Herpetosiphonaceae__Herpetosiphon__nan_	-	-	-	-	2.909	-	-	-	-	-	-	-	-	-	-	-	-	-	-	-	-	-	-	-	-
Acidobacteriota__nan__nan__nan__nan__nan_	-	-	-	-	-	-	-	-	-	-	1.021	-	-	-	-	-	-	-	-	-	-	-	-	-	-
Cyanobacteria__Sericytochromatia__nan__nan__nan__nan_	-	-	-	-	-	-	0.392	-	-	-	-	-	-	-	-	-	-	-	-	-	-	-	-	-	-
Verrucomicrobiota__Verrucomicrobiae__nan__nan__nan__nan_	-	-	-	-	-	-	0.411	-	-	-	-	-	-	-	-	-	-	-	-	-	-	-	-	-	-
Verrucomicrobiota__Verrucomicrobiae__Chthoniobacterales__Terrimicrobiaceae__FukuN18_freshwater_group__nan_	-	-	-	-	-	-	-	-	-	-	-	-	-	0.375	-	-	-	-	-	-	-	-	-	-	-
Verrucomicrobiota__Verrucomicrobiae__Verrucomicrobiales__nan__nan__nan_	-	-	-	-	-	-	-	-	-	-	-	-	-	-	-	-	-	-	-	-	-	2.911	-	-	-
Verrucomicrobiota__Verrucomicrobiae__nan__nan__nan__nan_	-	-	-	-	-	-	-	-	-	-	-	-	-	-	1.757	-	-	-	-	-	-	-	-	-	-
Planctomycetota__Planctomycetes__Gemmatales__Gemmataceae__Gemmata__nan_	-	-	-	-	-	-	-	-	0.429	-	-	-	-	-	-	-	-	-	-	-	-	-	-	-	-
Acidobacteriota__Vicinamibacteria__Subgroup_17__nan__nan__nan_	-	-	-	-	-	-	-	-	-	-	-	-	0.627	-	-	-	-	-	-	-	-	-	-	-	-
Verrucomicrobiota__Verrucomicrobiae__Verrucomicrobiales__Verrucomicrobiaceae__nan__nan_	-	-	-	-	-	-	0.392	-	-	-	-	-	-	-	-	-	-	-	-	-	-	-	-	-	-
Verrucomicrobiota__Verrucomicrobiae__Verrucomicrobiales__Verrucomicrobiaceae__Prosthecobacter__nan_	-	-	-	-	-	-	-	-	-	-	-	-	0.627	-	-	-	-	-	-	-	-	-	-	-	-
Acidobacteriota__Blastocatellia__Blastocatellales__Blastocatellaceae__nan__nan__	-	-	-	-	-	-	-	-	-	-	-	-	-	-	-	-	1.038	-	-	-	-	-	-	-	-
Acidobacteriota__Blastocatellia__Blastocatellales__Blastocatellaceae__JGI_0001001-H03__nan_	0.873	-	-	-	-	-	-	-	-	-	-	-	-	-	-	-	-	-	-	-	-	-	-	-	-
Acidobacteriota__Subgroup_25__nan__nan__nan__nan_	-	-	-	-	-	-	-	-	0.429	-	-	-	-	-	-	-	-	-	-	-	-	-	-	-	-
Verrucomicrobiota__Verrucomicrobiae__Opitutales__Opitutaceae__Opitutus__nan_	-	-	-	-	-	-	-	-	-	-	-	4.511	-	-	-	-	-	-	-	-	-	-	-	-	-
Verrucomicrobiota__Verrucomicrobiae__Opitutales__Opitutaceae__Opitutus__nan_	-	-	1.955	-	-	-	-	-	-	-	-	-	-	-	-	-	-	-	-	-	-	-	-	-	-
Verrucomicrobiota__Verrucomicrobiae__Opitutales__Opitutaceae__Opitutus__nan_	-	-	-	-	-	-	-	-	-	-	-	-	-	-	-	-	-	-	-	0.782	-	-	-	-	-
Chloroflexi__TK10__nan__nan__nan__nan_	-	-	-	-	-	-	-	-	0.485	-	-	-	-	-	-	-	-	-	-	-	-	-	-	-	-
Deinococcota__Deinococci__Deinococcales__Deinococcaceae__Deinococcus__nan_	-	-	-	-	-	-	-	-	-	6.446	-	-	-	-	-	-	-	-	-	-	-	-	-	-	-
Deinococcota__Deinococci__Deinococcales__Deinococcaceae__Deinococcus__nan_	-	-	-	-	-	-	-	-	-	5.818	-	-	-	-	-	-	-	-	-	-	-	-	-	-	-
Chloroflexi__Chloroflexia__Chloroflexales__Roseiflexaceae__nan__nan_	-	-	-	0.886	-	-	-	-	-	-	-	-	-	-	-	-	-	-	-	-	-	-	-	-	-
Thermoplasmatota__nan__nan__nan__nan__nan_	-	-	-	-	-	-	-	-	-	-	-	-	-	-	-	-	-	-	-	0.760	-	-	-	-	-
Deinococcota__Deinococci__Deinococcales__Deinococcaceae__Deinococcus__nan_	-	-	-	-	-	-	-	4.624	-	-	-	-	-	-	-	-	-	-	-	-	-	-	-	-	-
Actinobacteriota__Actinobacteria__Corynebacteriales__Nocardiaceae__Gordonia__nan_	-	-	1.956	-	-	-	-	-	-	-	-	-	-	-	-	-	-	-	-	-	-	-	-	-	-
Bacteroidota__Bacteroidia__Cytophagales__Spirosomaceae__Spirosoma__nan_	-	-	-	-	-	-	-	-	-	3.699	-	-	-	-	-	-	-	-	-	-	-	-	-	-	-
Actinobacteriota__Actinobacteria__Propionibacteriales__Nocardioidaceae__Nocardioides__nan_	-	-	-	-	-	-	-	4.694	-	-	-	-	-	-	-	-	-	-	-	-	-	-	-	-	-
Actinobacteriota__Actinobacteria__Propionibacteriales__Nocardioidaceae__Nocardioides__terrigena_	-	-	-	-	2.927	-	-	-	-	-	-	-	-	-	-	-	-	-	-	-	-	-	-	-	-
Gemmatimonadota__Longimicrobia__Longimicrobiales__Longimicrobiaceae__YC-ZSS-LKJ147__nan__	0.873	-	-	-	-	-	-	-	-	-	-	-	-	-	-	-	-	-	-	-	-	-	-	-	-
Actinobacteriota__Thermoleophilia__Solirubrobacterales__67-14__nan__nan_	-	-	-	-	-	-	-	-	-	-	-	-	-	-	-	-	-	-	-	-	1.256	-	-	-	-
Actinobacteriota__Thermoleophilia__Gaiellales__Gaiellaceae__Gaiella__nan_	-	-	-	0.886	-	-	-	-	-	-	-	-	-	-	-	-	-	-	-	-	-	-	-	-	-
Actinobacteriota__Thermoleophilia__Gaiellales__nan__nan__nan_	-	-	-	-	-	-	-	-	-	-	-	-	-	-	-	1.589	-	-	-	-	-	-	-	-	-
Actinobacteriota__Acidimicrobiia__nan__nan__nan__nan__	-	-	-	-	-	-	-	4.679	-	-	-	-	-	-	-	-	-	-	-	-	-	-	-	-	-
Actinobacteriota__Actinobacteria__Micromonosporales__Micromonosporaceae__nan__nan_	-	-	-	-	-	-	-	-	-	-	-	-	-	-	-	-	-	-	-	0.760	-	-	-	-	-
Bacteroidota__Bacteroidia__Flavobacteriales__Flavobacteriaceae__Flavobacterium__nan_	-	-	-	-	-	0.691	-	-	-	-	-	-	-	-	-	-	-	-	-	-	-	-	-	-	-
Bacteroidota__Bacteroidia__Flavobacteriales__Flavobacteriaceae__Flavobacterium__nan_	-	-	-	-	-	-	-	4.786	-	-	-	-	-	-	-	-	-	-	-	-	-	-	-	-	-
Bacteroidota__Bacteroidia__Flavobacteriales__Weeksellaceae__Chryseobacterium__nan_	-	2.165	-	-	-	-	-	-	-	-	-	-	-	-	-	-	-	-	-	-	-	-	-	-	-
Bacteroidota__Bacteroidia__Cytophagales__Hymenobacteraceae__Hymenobacter__gummosus/paludis_	-	-	-	-	-	-	-	-	-	5.953	-	-	-	-	-	-	-	-	-	-	-	-	-	-	-
Bacteroidota__Bacteroidia__Cytophagales__Hymenobacteraceae__Hymenobacter__nan_	0.873	-	-	-	-	-	-	-	-	-	-	-	-	-	-	-	-	-	-	-	-	-	-	-	-
Actinobacteriota__Actinobacteria__Kineosporiales__Kineosporiaceae__nan__nan_	-	-	-	-	-	-	-	-	-	-	-	-	-	-	1.738	-	-	-	-	-	-	-	-	-	-
Actinobacteriota__Actinobacteria__Kineosporiales__Kineosporiaceae__Kineosporia__aurantiaca/mikuniensis_	-	-	-	-	-	-	-	-	-	-	-	-	-	-	-	-	-	-	-	0.760	-	-	-	-	-
Bacteroidota__Bacteroidia__Sphingobacteriales__KD3-93__nan__nan_	-	-	-	-	-	-	-	-	-	-	-	-	-	-	-	-	-	-	-	-	-	-	-	1.955	-
Bacteroidota__Bacteroidia__Sphingobacteriales__KD3-93__nan__nan_	-	-	-	-	-	-	-	-	-	-	-	-	-	-	-	-	-	2.656	-	-	-	-	-	-	-
Bacteroidota__Bacteroidia__Sphingobacteriales__Sphingobacteriaceae__nan__nan_	-	-	-	-	-	-	-	-	-	-	-	-	-	-	-	-	-	-	-	-	-	-	-	-	3.445
Desulfobacterota__nan__nan__nan__nan__nan_	-	-	-	-	-	-	-	-	-	-	-	-	-	-	-	-	-	-	1.227	-	-	-	-	-	-
Bacteroidota__Bacteroidia__Chitinophagales__Chitinophagaceae__Parafilimonas__nan_	-	-	-	-	-	-	-	-	-	-	-	-	-	-	-	-	1.081	-	-	-	-	-	-	-	-
Bacteroidota__Bacteroidia__Chitinophagales__Chitinophagaceae__nan__nan_	-	-	-	-	-	-	-	-	-	-	-	-	-	0.385	-	-	-	-	-	-	-	-	-	-	-
Bacteroidota__Bacteroidia__Sphingobacteriales__Sphingobacteriaceae__nan__nan_	-	-	-	-	-	-	-	-	-	-	-	4.490	-	-	-	-	-	-	-	-	-	-	-	-	-
Bacteroidota__Bacteroidia__Sphingobacteriales__Sphingobacteriaceae__Arcticibacter__nan_	-	2.085	-	-	-	-	-	-	-	-	-	-	-	-	-	-	-	-	-	-	-	-	-	-	-
Bacteroidota__Bacteroidia__Chitinophagales__Chitinophagaceae__Flavisolibacter__nan_	-	-	-	-	-	-	-	4.613	-	-	-	-	-	-	-	-	-	-	-	-	-	-	-	-	-
Bacteroidota__Bacteroidia__Cytophagales__nan__nan__nan_	-	-	-	-	-	-	-	-	-	-	-	-	-	-	-	-	-	-	-	0.812	-	-	-	-	-
Verrucomicrobiota__Chlamydiae__Chlamydiales__nan__nan__nan_	-	-	-	-	-	-	-	-	-	-	-	-	-	-	-	-	-	-	-	-	1.256	-	-	-	-
Verrucomicrobiota__Chlamydiae__Chlamydiales__Parachlamydiaceae__nan__nan_	-	-	-	1.065	-	-	-	-	-	-	-	-	-	-	-	-	-	-	-	-	-	-	-	-	-
Verrucomicrobiota__Chlamydiae__Chlamydiales__Parachlamydiaceae__Candidatus_Protochlamydia__nan_	-	-	-	-	-	0.712	-	-	-	-	-	-	-	-	-	-	-	-	-	-	-	-	-	-	-
Actinobacteriota__Acidimicrobiia__Microtrichales__Microtrichaceae__IMCC26207__nan__	-	-	-	-	-	-	-	-	-	-	-	-	-	-	-	-	-	-	-	-	1.256	-	-	-	-
Armatimonadota__Fimbriimonadia__Fimbriimonadales__Fimbriimonadaceae__nan__nan__	-	-	-	-	-	-	-	-	-	-	-	-	-	-	1.868	-	-	-	-	-	-	-	-	-	-
Myxococcota__Polyangia__Blfdi19__nan__nan__nan_	-	-	-	-	-	-	-	-	-	-	-	-	-	-	-	-	-	-	-	-	-	-	-	1.937	-
Actinobacteriota__Acidimicrobiia__Microtrichales__nan__nan__nan__	-	-	-	-	-	-	-	-	-	-	-	-	-	-	-	-	-	-	1.168	-	-	-	-	-	-
Bacteroidota__Bacteroidia__Chitinophagales__Chitinophagaceae__nan__nan_	-	-	-	-	-	-	-	-	-	-	-	-	-	-	-	-	-	2.611	-	-	-	-	-	-	-
Proteobacteria__Gammaproteobacteria__Salinisphaerales__Solimonadaceae__Polycyclovorans__nan_	-	-	-	-	-	-	-	-	-	-	-	-	-	-	-	-	-	-	-	-	-	1.990	-	-	-
Acidobacteriota__Acidobacteriae__Solibacterales__Solibacteraceae__Candidatus_Solibacter__nan__	-	-	-	-	-	-	-	-	-	-	0.952	-	-	-	-	-	-	-	-	-	-	-	-	-	-
Proteobacteria__Alphaproteobacteria__Acetobacterales__Acetobacteraceae__Roseomonas__nan_	-	-	-	-	-	-	-	-	-	-	-	-	-	-	-	1.588	-	-	-	-	-	-	-	-	-
Proteobacteria__Alphaproteobacteria__Caulobacterales__Caulobacteraceae__Brevundimonas__bacteroides/staleyi/variabilis_	-	-	-	-	-	-	-	-	-	3.678	-	-	-	-	-	-	-	-	-	-	-	-	-	-	-
Proteobacteria__Alphaproteobacteria__Sphingomonadales__Sphingomonadaceae__nan__nan_	-	-	-	-	-	-	-	-	-	-	-	-	-	-	-	-	-	-	-	-	-	-	-	-	3.278
Proteobacteria__Alphaproteobacteria__Sphingomonadales__Sphingomonadaceae__Sphingomonas__nan_	-	-	-	-	-	-	-	-	-	-	-	-	-	-	-	-	-	-	-	-	-	2.175	-	-	-
Proteobacteria__Alphaproteobacteria__Sphingomonadales__Sphingomonadaceae__Croceicoccus__nan_	-	-	-	-	-	-	-	-	-	-	-	-	-	-	-	-	-	2.696	-	-	-	-	-	-	-
Proteobacteria__Alphaproteobacteria__Sphingomonadales__Sphingomonadaceae__Novosphingobium__nan_	-	-	-	-	-	-	-	-	-	-	-	-	-	-	-	-	-	-	-	-	-	1.994	-	-	-
Proteobacteria__Alphaproteobacteria__Rhizobiales__Rhizobiaceae__Aminobacter__nan_	-	-	-	-	-	-	-	-	-	-	-	-	-	-	-	-	-	-	-	-	-	-	1.376	-	-
Proteobacteria__Alphaproteobacteria__Rhizobiales__Devosiaceae__Devosia__nan_	-	-	-	-	-	-	-	-	-	-	0.978	-	-	-	-	-	-	-	-	-	-	-	-	-	-
Myxococcota__Polyangia__Haliangiales__Haliangiaceae__Haliangium__nan_	-	-	-	-	-	-	-	-	-	-	-	-	-	-	-	-	-	2.756	-	-	-	-	-	-	-
Proteobacteria__Alphaproteobacteria__Rhizobiales__nan__nan__nan_	-	-	-	-	-	-	-	-	-	-	-	-	-	-	-	-	-	-	-	-	-	-	-	-	3.355
GAL15__nan__nan__nan__nan__nan_	-	-	-	-	-	-	-	-	-	-	-	-	-	0.452	-	-	-	-	-	-	-	-	-	-	-
Proteobacteria__Alphaproteobacteria__Rickettsiales__Rickettsiaceae__nan__nan_	-	-	-	-	-	-	-	-	-	-	-	-	-	-	-	-	1.127	-	-	-	-	-	-	-	-
Actinobacteriota__Thermoleophilia__Solirubrobacterales__67-14__nan__nan_	-	-	-	-	-	-	-	-	-	-	-	-	-	-	-	-	1.029	-	-	-	-	-	-	-	-
Proteobacteria__Gammaproteobacteria__nan__nan__nan__nan_	-	-	-	-	-	-	-	-	-	-	-	-	-	-	-	-	-	-	-	-	1.256	-	-	-	-
Bdellovibrionota__Bdellovibrionia__Bacteriovoracales__Bacteriovoracaceae__Peredibacter__nan_	-	-	-	-	-	-	-	-	-	-	-	-	-	-	-	-	-	-	-	-	-	-	1.403	-	-
Proteobacteria__Alphaproteobacteria__Tistrellales__Geminicoccaceae__Candidatus_Alysiosphaera__nan_	-	-	-	-	-	-	-	-	0.485	-	-	-	-	-	-	-	-	-	-	-	-	-	-	-	-
Proteobacteria__Alphaproteobacteria__Rhizobiales__Beijerinckiaceae__Psychroglaciecola__nan_	-	-	1.958	-	-	-	-	-	-	-	-	-	-	-	-	-	-	-	-	-	-	-	-	-	-
Gemmatimonadota__S0134_terrestrial_group__nan__nan__nan__nan__	-	-	-	-	-	-	-	-	-	-	-	-	0.627	-	-	-	-	-	-	-	-	-	-	-	-
Proteobacteria__Alphaproteobacteria__Azospirillales__Azospirillaceae__Skermanella__aerolata_	-	-	-	-	-	0.644	-	-	-	-	-	-	-	-	-	-	-	-	-	-	-	-	-	-	-
Proteobacteria__Alphaproteobacteria__Azospirillales__Azospirillaceae__nan__nan_	-	-	-	-	-	-	-	-	-	-	-	-	-	-	-	-	-	-	-	-	-	-	-	1.999	-
Firmicutes__Bacilli__nan__nan__nan__nan	-	-	1.892	-	-	-	-	-	-	-	-	-	-	-	-	-	-	-	-	-	-	-	-	-	-
Proteobacteria__Alphaproteobacteria__Holosporales__Holosporaceae__nan__nan_	-	-	-	1.065	-	-	-	-	-	-	-	-	-	-	-	-	-	-	-	-	-	-	-	-	-
Firmicutes__Bacilli__Paenibacillales__Paenibacillaceae__Paenibacillus__nan	-	-	-	-	-	-	-	-	-	-	-	-	-	0.385	-	-	-	-	-	-	-	-	-	-	-
Gemmatimonadota__S0134_terrestrial_group__nan__nan__nan__nan_	-	-	-	-	-	-	-	-	-	-	-	-	0.630	-	-	-	-	-	-	-	-	-	-	-	-
Proteobacteria__Gammaproteobacteria__nan__nan__nan__nan_	0.873	-	-	-	-	-	-	-	-	-	-	-	-	-	-	-	-	-	-	-	-	-	-	-	-
Firmicutes__Bacilli__Bacillales__Planococcaceae__nan__nan_	-	-	-	-	-	-	-	-	-	-	-	-	-	-	-	-	1.049	-	-	-	-	-	-	-	-
Myxococcota__Polyangia__Polyangiales__Polyangiaceae__nan__nan__	-	-	-	-	-	-	-	-	-	-	-	-	-	0.370	-	-	-	-	-	-	-	-	-	-	-
Proteobacteria__Alphaproteobacteria__Rickettsiales__Rickettsiaceae__nan__nan_	-	-	-	-	-	-	-	-	-	-	-	-	-	-	-	-	-	-	-	-	1.310	-	-	-	-
Myxococcota__Polyangia__Polyangiales__nan__nan__nan_	-	-	-	-	-	-	-	-	-	-	-	-	-	-	-	-	-	-	1.366	-	-	-	-	-	-
Myxococcota__Polyangia__Blfdi19__nan__nan__nan_	-	-	-	-	-	-	0.415	-	-	-	-	-	-	-	-	-	-	-	-	-	-	-	-	-	-
Actinobacteriota__Thermoleophilia__Solirubrobacterales__Solirubrobacteraceae__Conexibacter__nan_	-	-	-	-	-	-	-	-	-	-	-	-	-	-	-	-	-	-	-	-	-	-	-	1.977	-
Actinobacteriota__Thermoleophilia__Solirubrobacterales__Solirubrobacteraceae__Conexibacter__nan_	-	-	-	-	-	-	-	-	-	-	-	-	-	-	-	-	-	-	-	-	-	-	-	1.941	-
Proteobacteria__Alphaproteobacteria__Reyranellales__Reyranellaceae__Reyranella__nan_	-	-	1.864	-	-	-	-	-	-	-	-	-	-	-	-	-	-	-	-	-	-	-	-	-	-
Proteobacteria__Gammaproteobacteria__nan__nan__nan__nan	-	-	-	-	-	-	-	-	-	-	-	6.550	-	-	-	-	-	-	-	-	-	-	-	-	-
Bdellovibrionota__Oligoflexia__0319-6G20__nan__nan__nan_	-	-	-	-	-	-	-	-	-	-	-	4.586	-	-	-	-	-	-	-	-	-	-	-	-	-
Proteobacteria__Gammaproteobacteria__Burkholderiales__Oxalobacteraceae__Massilia__nan_	-	-	-	-	2.900	-	-	-	-	-	-	-	-	-	-	-	-	-	-	-	-	-	-	-	-
Proteobacteria__Gammaproteobacteria__nan__nan__nan__nan_	-	-	-	-	-	0.618	-	-	-	-	-	-	-	-	-	-	-	-	-	-	-	-	-	-	-
Proteobacteria__Gammaproteobacteria__Burkholderiales__TRA3-20__nan__nan_	-	-	-	-	-	-	-	-	-	-	-	-	-	-	-	1.594	-	-	-	-	-	-	-	-	-
Proteobacteria__Gammaproteobacteria__Burkholderiales__Oxalobacteraceae__Noviherbaspirillum__nan_	-	-	-	-	-	-	-	-	-	-	-	-	-	-	-	-	-	-	-	-	-	-	1.403	-	-
Acidobacteriota__Acidobacteriae__Bryobacterales__Bryobacteraceae__Bryobacter__nan_	-	-	-	-	-	-	-	-	-	-	-	-	-	-	-	-	-	-	1.366	-	-	-	-	-	-
Planctomycetota__vadinHA49__nan__nan__nan__nan_	-	-	-	-	-	-	-	-	-	-	-	-	-	-	-	-	-	-	-	-	-	-	-	-	3.273
Planctomycetota__vadinHA49__nan__nan__nan__nan_	-	2.435	-	-	-	-	-	-	-	-	-	-	-	-	-	-	-	-	-	-	-	-	-	-	-
Proteobacteria__Gammaproteobacteria__Legionellales__Legionellaceae__Legionella__nan_	-	-	-	-	-	-	-	-	-	-	-	4.525	-	-	-	-	-	-	-	-	-	-	-	-	-
Proteobacteria__Gammaproteobacteria__Legionellales__Legionellaceae__Legionella__nan_	-	2.072	-	-	-	-	-	-	-	-	-	-	-	-	-	-	-	-	-	-	-	-	-	-	-
Proteobacteria__Gammaproteobacteria__Xanthomonadales__Xanthomonadaceae__Luteimonas__rhizosphaerae_	-	-	-	-	2.898	-	-	-	-	-	-	-	-	-	-	-	-	-	-	-	-	-	-	-	-
Proteobacteria__Gammaproteobacteria__Xanthomonadales__Xanthomonadaceae__Lysobacter__enzymogenes_	-	-	-	-	2.995	-	-	-	-	-	-	-	-	-	-	-	-	-	-	-	-	-	-	-	-
Proteobacteria__Gammaproteobacteria__Gammaproteobacteria_Incertae_Sedis__Unknown_Family__Acidibacter__nan_	-	-	-	-	-	-	-	-	-	-	-	-	-	-	-	-	-	-	1.372	-	-	-	-	-	-
Dependentiae__Babeliae__Babeliales__nan__nan__nan_	-	-	-	-	-	-	-	-	-	-	-	-	-	-	-	-	-	-	-	-	-	-	-	-	3.450
Chloroflexi__Anaerolineae__SBR1031__A4b__nan__nan_	-	-	-	-	-	-	-	-	-	-	1.021	-	-	-	-	-	-	-	-	-	-	-	-	-	-
Chloroflexi__Anaerolineae__SBR1031__A4b__nan__nan_	-	-	-	-	-	0.618	-	-	-	-	-	-	-	-	-	-	-	-	-	-	-	-	-	-	-
Verrucomicrobiota__Chlamydiae__Chlamydiales__cvE6__nan__nan_	-	-	-	-	-	-	-	-	-	-	-	-	-	-	-	-	-	-	-	-	-	-	1.368	-	-
Verrucomicrobiota__Verrucomicrobiae__Pedosphaerales__Pedosphaeraceae__Ellin517__nan_	-	-	-	-	-	-	-	-	-	-	-	-	-	-	1.868	-	-	-	-	-	-	-	-	-	-
Cyanobacteria__Sericytochromatia__nan__nan__nan__nan_	-	-	-	-	-	-	-	-	-	-	-	-	-	-	-	-	-	-	-	-	-	-	1.382	-	-
Acidobacteriota__Acidobacteriae__Acidobacteriales__Acidobacteriaceae_(Subgroup_1)__nan__nan_	-	-	-	-	-	-	-	-	-	-	-	-	-	-	-	-	-	-	-	-	1.256	-	-	-	-
Acidobacteriota__Acidobacteriae__Acidobacteriales__Acidobacteriaceae_(Subgroup_1)__nan__nan_	-	-	-	-	-	-	-	-	0.486	-	-	-	-	-	-	-	-	-	-	-	-	-	-	-	-
Verrucomicrobiota__Chlamydiae__Chlamydiales__Simkaniaceae__nan__nan_	-	-	-	-	-	-	-	-	-	-	-	-	-	-	1.756	-	-	-	-	-	-	-	-	-	-
Myxococcota__Polyangia__mle1-27__nan__nan__nan_	-	2.145	-	-	-	-	-	-	-	-	-	-	-	-	-	-	-	-	-	-	-	-	-	-	-

Table 17: *ASV level*. Relative amplifications of ASVs in each LDA topic. Only five most amplified ASVs in each topic are shown. Amplifications were converted to percentages.
