# Supplementary material for: Latent Dirichlet Allocation modeling of environmental microbiomes
Source: PLoS Comput Biol. 2023 Jun 8;19(6):e1011075. doi: 10.1371/journal.pcbi.1011075 (PMC10249879; doi:10.1371/journal.pcbi.1011075)
Supplement: S18 Table — Statistically significant relationships between topics and plant traits based on Spearman’s rank correlation coefficient with Holm–Bonferroni correction. (PDF) [file pcbi.1011075.s033.pdf]

topic	response	correlation	p-value
Topic 9	Stem height	-0.622323	4.186909e-14
Topic 7	Stem height	-0.610016	1.784144e-13
Topic 14	Stem height	-0.574516	8.380912e-12
Topic 13	Stem height	-0.568971	1.467622e-11
Topic 9	Root biomass	0.565924	1.988042e-11
Topic 13	Root biomass	0.535145	3.610858e-10
Topic 7	Root biomass	0.504180	5.043032e-09
Topic 14	Root biomass	0.496297	9.471054e-09
Topic 17	Stem height	-0.479713	3.389113e-08
Topic 1	Stem height	-0.464991	9.944427e-08
Topic 9	Stem diameter	0.430547	1.021565e-06
Topic 23	Root biomass	0.429148	1.116978e-06
Topic 17	Root biomass	0.423508	1.594640e-06
Topic 24	Stem height	-0.411778	3.276476e-06
Topic 13	Stem diameter	0.407525	4.225824e-06
Topic 15	Stem height	-0.407223	4.302262e-06
Topic 4	Stem height	-0.402113	5.812507e-06
Topic 18	Root biomass	0.396696	7.953422e-06
Topic 23	Stem diameter	0.388007	1.300288e-05
Topic 5	Stem height	-0.382680	1.745729e-05
Topic 7	Stem diameter	0.380365	1.981017e-05
Topic 3	Stem height	-0.376847	2.396304e-05
Topic 11	Water use efficiency	0.376392	2.455636e-05
Topic 14	Stem diameter	0.370070	3.436356e-05
Topic 18	Stem height	-0.359548	5.920648e-05
Topic 15	Root biomass	0.345777	1.173133e-04
Topic 23	Stem height	-0.333961	2.058010e-04

Table 18: *ASV level.* Statistically significant relationships between topics and plant traits based on Spearman's rank correlation coefficient with Holm–Bonferroni correction.
