# Supplementary material for: Latent Dirichlet Allocation modeling of environmental microbiomes
Source: PLoS Comput Biol. 2023 Jun 8;19(6):e1011075. doi: 10.1371/journal.pcbi.1011075 (PMC10249879; doi:10.1371/journal.pcbi.1011075)
Supplement: S19 Table — Statistically significant differences between 212 ASVs and plant traits based on Spearman’s rank correlation coefficient with Holm–Bonferroni correction. Part 1. (PDF) [file pcbi.1011075.s034.pdf]

ASV	plant trait	correlation	p-value
Bacteroidota__Bacteroidia__Sphingobacteriales__env.OPS_17__nan__nan_	Height_cm	0.767264	2.559071e-24
Proteobacteria__Gammaproteobacteria__Burkholderiales__Comamonadaceae__Hydrogenophaga__nan_	Height_cm	0.735327	1.697101e-21
Proteobacteria__Gammaproteobacteria__Cellvibrionales__Cellvibrionaceae__Cellvibrio__nan_	Height_cm	0.729798	4.754105e-21
Planctomycetota__Planctomycetes__Gemmatales__Gemmataceae__Gemmata__nan_	Height_cm	0.714902	6.751725e-20
Bacteroidota__Bacteroidia__Sphingobacteriales__KD3-93__nan__nan_	Height_cm	0.708021	2.173802e-19
Proteobacteria__Gammaproteobacteria__Burkholderiales__Burkholderiaceae__Cupriavidus__gilardii_	RootDry_g	-0.703538	4.573490e-19
Proteobacteria__Alphaproteobacteria__nan__nan__nan__nan_	RootDry_g	-0.683025	1.160573e-17
Proteobacteria__Gammaproteobacteria__Burkholderiales__Comamonadaceae__nan__nan_	Height_cm	0.682092	1.336135e-17
Chloroflexi__nan__nan__nan__nan__nan__	Height_cm	0.676364	3.136137e-17
Acidobacteriota__Blastocatellia__24-Nov__nan__nan__nan_	Height_cm	0.670803	7.051679e-17
Planctomycetota__Planctomycetes__Planctomycetales__Rubinisphaeraceae__SH-PL14__nan_	Height_cm	0.670217	7.672040e-17
Verrucomicrobiota__Verrucomicrobiae__Pedosphaerales__Pedosphaeraceae__nan__nan_	Height_cm	0.669406	8.619746e-17
Verrucomicrobiota__Chlamydiae__Chlamydiales__Simkaniaceae__nan__nan_	RootDry_g	-0.661323	2.697639e-16
Proteobacteria__Gammaproteobacteria__Burkholderiales__Burkholderiaceae__Cupriavidus__gilardii_	Height_cm	0.654478	6.898714e-16
Proteobacteria__Alphaproteobacteria__Sphingomonadales__Sphingomonadaceae__Novosphingobium__nan_	RootDry_g	-0.653530	7.841752e-16
Verrucomicrobiota__Chlamydiae__Chlamydiales__Simkaniaceae__nan__nan__T	Height_cm	0.646486	2.003685e-15
Proteobacteria__Alphaproteobacteria__Sphingomonadales__Sphingomonadaceae__Novosphingobium__nan_	Stem_Diam	-0.644377	2.640885e-15
Bacteroidota__nan__nan__nan__nan__nan_	Height_cm	0.640935	4.125395e-15
Proteobacteria__Gammaproteobacteria__nan__nan__nan__nan_	Height_cm	0.633264	1.092708e-14
Proteobacteria__Gammaproteobacteria__Legionellales__Legionellaceae__Legionella__nan_	RootDry_g	-0.632468	1.207037e-14
Bacteroidota__Bacteroidia__Sphingobacteriales__env.OPS_17__nan__nan_	RootDry_g	-0.625951	2.697652e-14
Verrucomicrobiota__Chlamydiae__Chlamydiales__Simkaniaceae__nan__nan_	Height_cm	0.618327	6.750193e-14
Proteobacteria__Gammaproteobacteria__Cellvibrionales__Cellvibrionaceae__Cellvibrio__nan_	RootDry_g	-0.607295	2.437499e-13
Bdellovibrionota__Bdellovibrionia__Bdellovibrionales__Bdellovibrionaceae__Bdellovibrio__nan_	LMA	-0.599700	5.734814e-13
Proteobacteria__Alphaproteobacteria__Rhizobiales__Beijerinckiaceae__Methylobacterium-Methylorubrum__nan_	DroughtTime	-0.594672	9.980465e-13
Planctomycetota__Planctomycetes__Planctomycetales__Schlesneriaceae__Planctopirus__nan_	Height_cm	0.594228	1.047694e-12
Proteobacteria__Gammaproteobacteria__Legionellales__Legionellaceae__Legionella__nan_	Stem_Diam	-0.585918	2.560466e-12
Proteobacteria__Alphaproteobacteria__nan__nan__nan__nan_	Stem_Diam	-0.585852	2.578585e-12
Proteobacteria__Gammaproteobacteria__Burkholderiales__Burkholderiaceae__Cupriavidus__gilardii_	Stem_Diam	-0.579179	5.188872e-12
Chloroflexi__nan__nan__nan__nan__nan__	RootDry_g	-0.577055	6.461285e-12
Proteobacteria__Gammaproteobacteria__Burkholderiales__Nitrosomonadaceae__Ellin6067__nan_	Height_cm	-0.569543	1.385902e-11
Bdellovibrionota__Bdellovibrionia__Bdellovibrionales__Bdellovibrionaceae__Bdellovibrio__nan_	Height_cm	0.567255	1.741804e-11
Bdellovibrionota__Bdellovibrionia__Bacteriovoracales__Bacteriovoracaceae__Peredibacter__nan_	LMA	-0.566294	1.916405e-11
Proteobacteria__Gammaproteobacteria__Burkholderiales__Gallionellaceae__Gallionella__nan_	RootDry_g	-0.564733	2.236498e-11
Gemmatimonadota__Gemmatimonadetes__Gemmatimonadales__Gemmatimonadaceae__nan__nan_	Height_cm	0.561339	3.120600e-11
Proteobacteria__Alphaproteobacteria__Rickettsiales__Mitochondria__nan__na	Height_cm	0.559942	3.574981e-11
Proteobacteria__Alphaproteobacteria__nan__nan__nan__nan_	Height_cm	0.559698	3.660776e-11
Proteobacteria__Alphaproteobacteria__Caulobacterales__Caulobacteraceae__Phenylobacterium__nan_	Height_cm	0.558820	3.986040e-11
Bacteroidota__Bacteroidia__Sphingobacteriales__env.OPS_17__nan__nan_	RootDry_g	-0.550096	9.160448e-11
Proteobacteria__Alphaproteobacteria__Rhizobiales__Rhizobiales_Incertae_Sedis__Nordella__nan_	Height_cm	0.546398	1.294126e-10
Bacteroidota__Bacteroidia__Sphingobacteriales__env.OPS_17__nan__nan_	RootDry_g	0.544284	1.573885e-10
Verrucomicrobiota__Chlamydiae__Chlamydiales__Simkaniaceae__nan__nan_	Stem_Diam	-0.540537	2.218928e-10
Proteobacteria__Gammaproteobacteria__nan__nan__nan__nan_	LMA	-0.537464	2.931685e-10
Actinobacteriota__Actinobacteria__Pseudonocardiales__Pseudonocardiaceae__Pseudonocardia__nan__	Stem_Diam	-0.537462	2.932191e-10
Verrucomicrobiota__Chlamydiae__Chlamydiales__Simkaniaceae__nan__nan__T	RootDry_g	-0.537320	2.969809e-10
Proteobacteria__Alphaproteobacteria__Sphingomonadales__Sphingomonadaceae__Blastomonas__natatoria/ursincola_	Height_cm	-0.537093	3.031187e-10
Proteobacteria__Alphaproteobacteria__Rhizobiales__Beijerinckiaceae__Bosea__nan_	Height_cm	0.536915	3.080194e-10
Proteobacteria__Gammaproteobacteria__Burkholderiales__Comamonadaceae__Ideonella__nan_	Height_cm	0.535296	3.562258e-10
Proteobacteria__Gammaproteobacteria__Burkholderiales__Oxalobacteraceae__Massilia__nan_	RootDry_g	0.534630	3.780949e-10
Proteobacteria__Alphaproteobacteria__Rhizobiales__Rhizobiaceae__nan__nan_	Height_cm	0.532464	4.585288e-10
Proteobacteria__Gammaproteobacteria__Burkholderiales__Comamonadaceae__nan__nan_	LMA	-0.529620	5.894344e-10
Proteobacteria__Alphaproteobacteria__Rhizobiales__Rhizobiaceae__nan__nan_	RootDry_g	-0.529018	6.214401e-10
Proteobacteria__Alphaproteobacteria__Reyranellales__Reyranellaceae__Reyranella__nan_	RootDry_g	-0.526453	7.775426e-10
Bacteroidota__Bacteroidia__Sphingobacteriales__env.OPS_17__nan__nan_	Height_cm	-0.526083	8.029813e-10
Bacteroidota__Bacteroidia__Sphingobacteriales__env.OPS_17__nan__nan_	Height_cm	-0.525278	8.611021e-10
Proteobacteria__Alphaproteobacteria__Rhodobacterales__Rhodobacteraceae__Rhodobacter__nan_	Stem_Diam	0.524518	9.196006e-10
Actinobacteriota__Actinobacteria__Propionibacteriales__Nocardioidaceae__Aeromicrobium__ponti_	RootDry_g	-0.521298	1.213128e-09
Acidobacteriota__Acidobacteriae__Acidobacteriae_or__Acidobacteriae_fa__Paludibaculum__nan_	Height_cm	0.521251	1.218034e-09
Bdellovibrionota__Oligoflexia__0319-6G20__nan__nan__nan_	RootDry_g	-0.520984	1.246102e-09
Bdellovibrionota__Bdellovibrionia__Bdellovibrionales__Bdellovibrionaceae__Bdellovibrio__nan_	Height_cm	0.519018	1.473397e-09
Bacteroidota__Bacteroidia__Sphingobacteriales__AKYH767__nan__nan_	RootDry_g	0.517802	1.633321e-09
Proteobacteria__Gammaproteobacteria__Pseudomonadales__Pseudomonadaceae__Pseudomonas__nan_	RootDry_g	0.516808	1.776317e-09
Proteobacteria__Gammaproteobacteria__Burkholderiales__Rhodocyclaceae__Sulfuritalea__nan_	Height_cm	0.511695	2.724345e-09
Proteobacteria__Alphaproteobacteria__Rhizobiales__Beijerinckiaceae__Bosea__nan_	Height_cm	-0.510524	3.001498e-09
Proteobacteria__Gammaproteobacteria__Salinisphaerales__Solimonadaceae__Alkanibacter__nan_	Stem_Diam	0.510260	3.067714e-09
Planctomycetota__Planctomycetes__Planctomycetales__Rubinisphaeraceae__SH-PL14__nan_	RootDry_g	-0.510199	3.083151e-09
Cyanobacteria__Cyanobacteriia__Cyanobacteriales__Nostocaceae__Calothrix_PCC-6303__nan_	Height_cm	0.508465	3.556292e-09
Proteobacteria__Alphaproteobacteria__Sphingomonadales__Sphingomonadaceae__Sphingobium__nan_	Stem_Diam	-0.507766	3.766164e-09
Actinobacteriota__Actinobacteria__Propionibacteriales__Nocardioidaceae__Aeromicrobium__ponti_	Height_cm	0.506178	4.287576e-09
Gemmatimonadota__Gemmatimonadetes__Gemmatimonadales__Gemmatimonadaceae__nan__nan_	DroughtTime	0.505957	4.365467e-09
Proteobacteria__Alphaproteobacteria__Caulobacterales__Caulobacteraceae__Phenylobacterium__nan_	RootDry_g	-0.505495	4.532641e-09
Proteobacteria__Gammaproteobacteria__Burkholderiales__Comamonadaceae__Hydrogenophaga__nan_	RootDry_g	-0.504789	4.800407e-09
Bacteroidota__Bacteroidia__Sphingobacteriales__env.OPS_17__nan__nan_	Stem_Diam	0.500683	6.682753e-09
Bacteroidota__Bacteroidia__Chitinophagales__Chitinophagaceae__Lacibacter__nan_	Stem_Diam	0.500339	6.869300e-09
Verrucomicrobiota__Verrucomicrobiae__Verrucomicrobiales__Verrucomicrobiaceae__nan__nan_	DroughtTime	0.500308	6.886533e-09
Proteobacteria__Gammaproteobacteria__Oceanospirillales__Pseudohongiellaceae__Pseudohongiella__nan_	LMA	-0.498879	7.718235e-09
Proteobacteria__Alphaproteobacteria__Sphingomonadales__Sphingomonadaceae__Blastomonas__natatoria/ursincola_	Stem_Diam	0.497502	8.610230e-09
Cyanobacteria__Cyanobacteriia__Chloroplast__nan__nan__nan_	Height_cm	0.497343	8.719458e-09
Proteobacteria__Gammaproteobacteria__Legionellales__Legionellaceae__Legionella__nan_	Height_cm	0.497120	8.874207e-09
Bacteroidota__Bacteroidia__Cytophagales__nan__nan__nan_	Height_cm	0.495607	1.000028e-08
Proteobacteria__Alphaproteobacteria__Rhizobiales__Rhizobiaceae__nan__nan_	Height_cm	0.495467	1.011124e-08
Proteobacteria__Alphaproteobacteria__Rhizobiales__Xanthobacteraceae__Pseudolabrys__nan_	RootDry_g	-0.495352	1.020354e-08
Bacteroidota__Bacteroidia__Sphingobacteriales__env.OPS_17__nan__nan_	Height_cm	-0.494854	1.061075e-08
Verrucomicrobiota__Chlamydiae__Chlamydiales__nan__nan__nan_	Height_cm	-0.493725	1.159342e-08
Crenarchaeota__Nitrososphaeria__Nitrososphaerales__Nitrososphaeraceae__nan__nan_	Stem_Diam	-0.492876	1.238903e-08
Bacteroidota__Bacteroidia__Sphingobacteriales__KD3-93__nan__nan_	RootDry_g	0.492650	1.260940e-08
Planctomycetota__Planctomycetes__Planctomycetales__Rubinisphaeraceae__SH-PL14__nan_	RootDry_g	-0.492079	1.318313e-08
Proteobacteria__Gammaproteobacteria__Cellvibrionales__Cellvibrionaceae__Cellvibrio__nan_	LMA	-0.491157	1.416323e-08
Proteobacteria__Gammaproteobacteria__Burkholderiales__Comamonadaceae__nan__nan_	RootDry_g	-0.489857	1.566331e-08
Proteobacteria__Alphaproteobacteria__Sphingomonadales__Sphingomonadaceae__Novosphingobium__nan_	Height_cm	0.489663	1.590103e-08
Bacteroidota__Bacteroidia__Sphingobacteriales__KD3-93__nan__nan_	Stem_Diam	0.488994	1.674277e-08
Proteobacteria__Alphaproteobacteria__Rickettsiales__Rickettsiaceae__nan__nan_	Height_cm	0.487375	1.896302e-08
Proteobacteria__Gammaproteobacteria__Burkholderiales__Gallionellaceae__Gallionella__nan_	Stem_Diam	-0.487014	1.949506e-08
Proteobacteria__Gammaproteobacteria__Burkholderiales__Comamonadaceae__Curvibacter__nan_	Height_cm	-0.486079	2.094174e-08
Proteobacteria__nan__nan__nan__nan__nan_	Stem_Diam	-0.485051	2.264892e-08
Proteobacteria__Alphaproteobacteria__Rhizobiales__Hyphomicrobiaceae__Hyphomicrobium__zavarzinii_	Height_cm	0.483542	2.539931e-08
Proteobacteria__Alphaproteobacteria__Rhizobiales__Rhizobiaceae__nan__nan_	Height_cm	0.482905	2.665533e-08
Bacteroidota__Bacteroidia__Flavobacteriales__Flavobacteriaceae__Flavobacterium__cheonhonense_	Height_cm	0.482348	2.780020e-08
Bacteroidota__Bacteroidia__Cytophagales__Microscillaceae__Chryseolinea__nan_	Height_cm	-0.481763	2.905444e-08
Bacteroidota__Bacteroidia__Sphingobacteriales__env.OPS_17__nan__nan_	Stem_Diam	-0.481733	2.912008e-08
Acidobacteriota__Vicinamibacteria__Vicinamibacterales__Vicinamibacteraceae__nan__nan_	LMA	-0.480858	3.110145e-08
Proteobacteria__Gammaproteobacteria__Burkholderiales__Rhodocyclaceae__Sulfuritalea__nan_	RootDry_g	-0.480417	3.214724e-08
Bacteroidota__Bacteroidia__Sphingobacteriales__AKYH767__nan__nan_	Height_cm	-0.480323	3.237599e-08
Bacteroidota__Bacteroidia__Chitinophagales__Chitinophagaceae__Flavisolibacter__nan_	Stem_Diam	-0.479480	3.448537e-08
Proteobacteria__Gammaproteobacteria__Burkholderiales__Methylophilaceae__Methylophilus__nan_	LMA	-0.478131	3.813990e-08
Actinobacteriota__Actinobacteria__Pseudonocardiales__Pseudonocardiaceae__Pseudonocardia__nan__	RootDry_g	-0.478015	3.847082e-08
Actinobacteriota__Actinobacteria__Propionibacteriales__Nocardioidaceae__Nocardioides__nan_	DroughtTime	-0.477152	4.102014e-08
Patescibacteria__Saccharimonadia__Saccharimonadales__Saccharimonadaceae__TM7a__nan__T	RootDry_g	-0.476860	4.191883e-08
Planctomycetota__Planctomycetes__Planctomycetales__Schlesneriaceae__Planctopirus__nan_	LMA	-0.476854	4.193729e-08
Bacteroidota__Bacteroidia__Sphingobacteriales__env.OPS_17__nan__nan_	RootDry_g	0.476765	4.221389e-08

Table 19: *ASV level.* Statistically significant differences between 212 ASVs (108 are unique) and plant traits based on Spearman's rank correlation coefficient with Holm–Bonferroni correction. The actual names of ASVs are not printed due to space, each ASV represented as $phylum\_class\_order\_family\_genus\_species$. Part 1.
