# Supplementary material for: Latent Dirichlet Allocation modeling of environmental microbiomes
Source: PLoS Comput Biol. 2023 Jun 8;19(6):e1011075. doi: 10.1371/journal.pcbi.1011075 (PMC10249879; doi:10.1371/journal.pcbi.1011075)
Supplement: S20 Table — Statistically significant differences between 212 ASVs and plant traits based on Spearman’s rank correlation coefficient with Holm–Bonferroni correction. Part 2. (PDF) [file pcbi.1011075.s035.pdf]

ASV	plant trait	correlation	p-value
Proteobacteria__Alphaproteobacteria__nan__nan__nan__nan_	Height_cm	0.476303	4.368517e-08
Actinobacteriota__Actinobacteria__Pseudonocardiales__Pseudonocardiaceae__Pseudonocardia__ailaonensis/xishanensis__	Height_cm	0.475195	4.741036e-08
Dependentiae__Babeliae__Babeliales__nan__nan__nan_	Stem_Diam	0.473976	5.186334e-08
Proteobacteria__Alphaproteobacteria__Rhizobiales__Beijerinckiaceae__Bosea__nan_	RootDry_g	-0.473740	5.277059e-08
Bacteroidota__Bacteroidia__Sphingobacteriales__KD3-93__nan__nan_	RootDry_g	0.473604	5.330224e-08
Proteobacteria__Gammaproteobacteria__Legionellales__Legionellaceae__Legionella__nan_	Height_cm	0.473408	5.407361e-08
Proteobacteria__Gammaproteobacteria__Burkholderiales__Nitrosomonadaceae__Nitrosomonas__nan_	Height_cm	0.473183	5.497347e-08
Actinobacteriota__Actinobacteria__Micrococcales__Micrococcaceae__Pseudarthrobacter__nan_	DroughtTime	-0.473121	5.522231e-08
Bdellovibrionota__Oligoflexia__0319-6G20__nan__nan__nan_	Stem_Diam	-0.472592	5.740385e-08
Acidobacteriota__Acidobacteriae__Acidobacteriales__Acidobacteriaceae_(Subgroup_1)__Edaphobacter__aggregans/flagellatus/modestus_	Height_cm	0.471848	6.061339e-08
Gemmatimonadota__Longimicrobia__Longimicrobiales__Longimicrobiaceae__nan__nan__	Height_cm	0.471675	6.138111e-08
Proteobacteria__Alphaproteobacteria__Rhizobiales__Hyphomicrobiaceae__Hyphomicrobium__nan_	Stem_Diam	-0.471657	6.146139e-08
Proteobacteria__Gammaproteobacteria__Xanthomonadales__Xanthomonadaceae__Arenimonas__nan_	Height_cm	-0.470431	6.720013e-08
Proteobacteria__Alphaproteobacteria__Rhizobiales__Rhizobiales_Incertae_Sedis__nan__nan_	Height_cm	0.470140	6.863762e-08
Bacteroidota__Bacteroidia__Chitinophagales__Chitinophagaceae__Flavisolibacter__nan_	LWC	-0.469950	6.958880e-08
Proteobacteria__Gammaproteobacteria__Burkholderiales__Gallionellaceae__Gallionella__nan_	RootDry_g	-0.467153	8.517179e-08
Bacteroidota__Bacteroidia__Chitinophagales__Chitinophagaceae__nan__nan_	Height_cm	-0.466996	8.614041e-08
Proteobacteria__Alphaproteobacteria__Rhizobiales__Xanthobacteraceae__Bradyrhizobium__nan_	Height_cm	0.466537	8.902626e-08
Myxococcota__Polyangia__Blfdi19__nan__nan__nan_	Stem_Diam	0.466504	8.923404e-08
Patescibacteria__Saccharimonadia__Saccharimonadales__Saccharimonadaceae__TM7a__nan__T	Stem_Diam	-0.465985	9.262282e-08
Proteobacteria__Gammaproteobacteria__Burkholderiales__Oxalobacteraceae__Massilia__nan_	Height_cm	-0.465981	9.264775e-08
Proteobacteria__Alphaproteobacteria__Rhizobiales__Xanthobacteraceae__Pseudolabrys__nan_	Height_cm	0.465680	9.466477e-08
Proteobacteria__Gammaproteobacteria__Burkholderiales__Comamonadaceae__Methylibium__petroleiphilum_	Stem_Diam	0.465591	9.526954e-08
Proteobacteria__Gammaproteobacteria__Salinisphaerales__Solimonadaceae__Hydrocarboniphaga__nan_	RootDry_g	0.464879	1.002412e-07
Chloroflexi__nan__nan__nan__nan__nan__	Stem_Diam	-0.463105	1.137369e-07
Verrucomicrobiota__Verrucomicrobiae__Pedosphaerales__Pedosphaeraceae__nan__nan_	Stem_Diam	0.462627	1.176594e-07
Proteobacteria__Alphaproteobacteria__Reyranellales__Reyranellaceae__Reyranella__nan_	Stem_Diam	-0.460720	1.346369e-07
Bacteroidota__Bacteroidia__Flavobacteriales__Flavobacteriaceae__Flavobacterium__cheonhonense_	RootDry_g	-0.460611	1.356732e-07
Bdellovibrionota__Bdellovibrionia__Bdellovibrionales__Bdellovibrionaceae__Bdellovibrio__nan_	Stem_Diam	-0.459887	1.427611e-07
Proteobacteria__Alphaproteobacteria__Rhizobiales__Beijerinckiaceae__nan__nan_	Height_cm	0.459208	1.497296e-07
Bacteroidota__Bacteroidia__Sphingobacteriales__KD3-93__nan__nan_	Height_cm	-0.459134	1.505075e-07
Acidobacteriota__Holophagae__Subgroup_7__nan__nan__nan_	Height_cm	-0.458932	1.526465e-07
Proteobacteria__Alphaproteobacteria__Sphingomonadales__Sphingomonadaceae__Porphyrobacter__tepidarius_	RootDry_g	-0.458826	1.537900e-07
Bacteroidota__Bacteroidia__Sphingobacteriales__Sphingobacteriaceae__nan__nan_	DroughtTime	-0.458208	1.605826e-07
Proteobacteria__Alphaproteobacteria__Sphingomonadales__Sphingomonadaceae__Porphyrobacter__tepidarius_	Stem_Diam	-0.456085	1.861775e-07
Proteobacteria__Alphaproteobacteria__Sphingomonadales__Sphingomonadaceae__Sphingomonas__nan_	Height_cm	0.455611	1.924022e-07
Proteobacteria__Gammaproteobacteria__Burkholderiales__Methylophilaceae__Methylophilus__nan_	Height_cm	0.455475	1.942197e-07
Myxococcota__Polyangia__Haliangiales__Haliangiaceae__Haliangium__nan_	Stem_Diam	-0.455076	1.996553e-07
Proteobacteria__Gammaproteobacteria__Burkholderiales__Oxalobacteraceae__Noviherbaspirillum__suwonense_	DroughtTime	-0.454977	2.010246e-07
Bacteroidota__Bacteroidia__Sphingobacteriales__KD3-93__nan__nan_	RootDry_g	-0.454228	2.117111e-07
Actinobacteriota__Actinobacteria__Pseudonocardiales__Pseudonocardiaceae__Pseudonocardia__nan__	Stem_Diam	-0.453529	2.221608e-07
Planctomycetota__Planctomycetes__Gemmatales__Gemmataceae__Fimbriiglobus__nan_	Height_cm	0.452228	2.429276e-07
Bacteroidota__Bacteroidia__Sphingobacteriales__env.OPS_17__nan__nan_	Stem_Diam	0.451938	2.478064e-07
Cyanobacteria__Vampirivibrionia__Obscuribacterales__Obscuribacteraceae__nan__nan_	RootDry_g	0.451132	2.618619e-07
Proteobacteria__Alphaproteobacteria__Rhizobiales__Beijerinckiaceae__Bosea__nan_	RootDry_g	0.449592	2.908427e-07
Bacteroidota__nan__nan__nan__nan__nan_	RootDry_g	-0.448427	3.147735e-07
Proteobacteria__Alphaproteobacteria__Caulobacterales__Caulobacteraceae__nan__nan_	Height_cm	0.447702	3.305904e-07
Verrucomicrobiota__Verrucomicrobiae__Chthoniobacterales__Chthoniobacteraceae__Chthoniobacter__nan_	Stem_Diam	0.447638	3.320240e-07
Planctomycetota__Planctomycetes__Gemmatales__Gemmataceae__Gemmata__nan_	RootDry_g	-0.447626	3.323012e-07
Proteobacteria__Gammaproteobacteria__Burkholderiales__Comamonadaceae__nan__nan_	Stem_Diam	0.447591	3.330718e-07
Elusimicrobiota__Elusimicrobia__Lineage_IV__nan__nan__nan_	Height_cm	-0.447408	3.372232e-07
Proteobacteria__Alphaproteobacteria__Caulobacterales__Caulobacteraceae__Phenylobacterium__nan_	Stem_Diam	-0.447306	3.395575e-07
Actinobacteriota__Thermoleophilia__nan__nan__nan__nan_	Height_cm	0.446152	3.669967e-07
Proteobacteria__Alphaproteobacteria__Rhizobiales__Hyphomicrobiaceae__Hyphomicrobium__zavarzinii_	RootDry_g	-0.445224	3.906013e-07
Verrucomicrobiota__Verrucomicrobiae__Pedosphaerales__Pedosphaeraceae__nan__nan_	Height_cm	-0.444872	3.999165e-07
Proteobacteria__Alphaproteobacteria__Rhizobiales__Rhizobiaceae__Mesorhizobium__nan_	RootDry_g	-0.444872	3.999268e-07
Proteobacteria__Alphaproteobacteria__Caulobacterales__Caulobacteraceae__nan__nan_	DroughtTime	0.444246	4.170312e-07
Verrucomicrobiota__Verrucomicrobiae__Pedosphaerales__Pedosphaeraceae__nan__nan_	LMA	-0.444201	4.182832e-07
Patescibacteria__Saccharimonadia__Saccharimonadales__Saccharimonadaceae__TM7a__nan__T	Height_cm	0.443960	4.250729e-07
Proteobacteria__Alphaproteobacteria__Rhizobiales__Beijerinckiaceae__Methylobacterium-Methylorubrum__nan_	DroughtTime	-0.443895	4.269269e-07
Proteobacteria__Alphaproteobacteria__Acetobacterales__Acetobacteraceae__nan__nan_	RootDry_g	-0.443776	4.303406e-07
Proteobacteria__Gammaproteobacteria__Burkholderiales__Burkholderiaceae__Ralstonia__nan_	RootDry_g	-0.443481	4.388851e-07
Proteobacteria__Alphaproteobacteria__Sphingomonadales__Sphingomonadaceae__Sphingomonas__sediminicola_	Height_cm	0.443374	4.420352e-07
Myxococcota__Myxococcia__Myxococcales__Myxococcaceae__nan__nan_	RootDry_g	-0.442944	4.548586e-07
Bacteroidota__Bacteroidia__Sphingobacteriales__env.OPS_17__nan__nan_	Height_cm	-0.442400	4.716245e-07
Proteobacteria__Gammaproteobacteria__Burkholderiales__Comamonadaceae__Curvibacter__nan_	RootDry_g	0.441254	5.088799e-07
Proteobacteria__Alphaproteobacteria__Rhizobiales__Hyphomicrobiaceae__Hyphomicrobium__nan_	RootDry_g	-0.440923	5.201626e-07
Proteobacteria__Gammaproteobacteria__Burkholderiales__Gallionellaceae__Gallionella__nan_	Stem_Diam	-0.440803	5.242973e-07
WPS-2__nan__nan__nan__nan__nan_	Stem_Diam	0.440439	5.370566e-07
Proteobacteria__Alphaproteobacteria__Rhizobiales__Xanthobacteraceae__Pseudolabrys__nan_	Height_cm	0.438954	5.922306e-07
Crenarchaeota__Nitrososphaeria__Nitrososphaerales__Nitrososphaeraceae__nan__nan_	RootDry_g	-0.438603	6.060566e-07
Bacteroidota__Bacteroidia__nan__nan__nan__nan_	Height_cm	-0.438306	6.179812e-07
Actinobacteriota__Actinobacteria__Frankiales__nan__nan__nan_	RootDry_g	-0.438045	6.286431e-07
Verrucomicrobiota__Verrucomicrobiae__Verrucomicrobiales__Verrucomicrobiaceae__nan__nan_	RootDry_g	-0.437929	6.334360e-07
Bacteroidota__Bacteroidia__Cytophagales__Microscillaceae__Ohtaekwangia__nan_	Height_cm	-0.437416	6.550943e-07
Actinobacteriota__Actinobacteria__Micrococcales__Intrasporangiaceae__Terrabacter__nan_	DroughtTime	-0.437077	6.697747e-07
Verrucomicrobiota__Verrucomicrobiae__Verrucomicrobiales__Verrucomicrobiaceae__Roseimicrobium__nan_	RootDry_g	-0.436687	6.870182e-07
Proteobacteria__Alphaproteobacteria__Ferrovibrionales__Ferrovibrionaceae__Ferrovibrio__nan_	DroughtTime	0.435576	7.385975e-07
Proteobacteria__Alphaproteobacteria__Rhizobiales__Rhizobiales_Incertae_Sedis__nan__nan_	RootDry_g	-0.434747	7.794111e-07
Proteobacteria__Alphaproteobacteria__Rhizobiales__Rhizobiales_Incertae_Sedis__nan__nan_	Height_cm	0.434007	8.177045e-07
Bacteroidota__Bacteroidia__Sphingobacteriales__env.OPS_17__nan__nan_	RootDry_g	0.433994	8.183731e-07
Bacteroidota__Bacteroidia__Sphingobacteriales__nan__nan__nan_	Stem_Diam	-0.433582	8.404974e-07
Proteobacteria__Alphaproteobacteria__Sphingomonadales__Sphingomonadaceae__Sphingobium__nan_	RootDry_g	-0.433101	8.670116e-07
Planctomycetota__Planctomycetes__Planctomycetales__Rubinisphaeraceae__SH-PL14__nan_	Stem_Diam	-0.432645	8.928653e-07
Proteobacteria__Gammaproteobacteria__Burkholderiales__Comamonadaceae__nan__nan_	Stem_Diam	-0.431776	9.441894e-07
Proteobacteria__Alphaproteobacteria__Rhizobiales__Hyphomicrobiaceae__Hyphomicrobium__zavarzinii_	Stem_Diam	-0.431586	9.557687e-07
Myxococcota__Polyangia__Polyangiales__Polyangiaceae__Pajaroellobacter__nan_	Height_cm	0.431572	9.566027e-07
Bacteroidota__Bacteroidia__Sphingobacteriales__env.OPS_17__nan__nan_	Height_cm	-0.431086	9.869098e-07
Proteobacteria__Alphaproteobacteria__Reyranellales__Reyranellaceae__Reyranella__nan_	Height_cm	0.429946	1.061601e-06
Bdellovibrionota__Oligoflexia__0319-6G20__nan__nan__nan_	Height_cm	0.429853	1.067928e-06
Bacteroidota__Bacteroidia__Sphingobacteriales__env.OPS_17__nan__nan_	LMA	-0.429824	1.069859e-06
Proteobacteria__Gammaproteobacteria__Burkholderiales__Comamonadaceae__Methylibium__petroleiphilum_	Height_cm	-0.429046	1.124305e-06
Proteobacteria__Gammaproteobacteria__Salinisphaerales__Solimonadaceae__Nevskia__nan_	Stem_Diam	0.428614	1.155588e-06
Actinobacteriota__Actinobacteria__Pseudonocardiales__Pseudonocardiaceae__Pseudonocardia__nan__	Stem_Diam	-0.428458	1.167144e-06
Proteobacteria__nan__nan__nan__nan__nan_	RootDry_g	-0.427989	1.202420e-06
Actinobacteriota__Actinobacteria__Pseudonocardiales__Pseudonocardiaceae__Pseudonocardia__nan__	RootDry_g	-0.427397	1.248392e-06
Proteobacteria__Alphaproteobacteria__Caulobacterales__Caulobacteraceae__Asticcacaulis__nan_	Height_cm	0.426907	1.287756e-06
Proteobacteria__Alphaproteobacteria__Sphingomonadales__Sphingomonadaceae__Sphingobium__nan_	Stem_Diam	-0.426705	1.304234e-06
Proteobacteria__Gammaproteobacteria__Salinisphaerales__Solimonadaceae__Polycyclovorans__nan_	Height_cm	-0.426522	1.319435e-06
Proteobacteria__Alphaproteobacteria__Acetobacterales__Acetobacteraceae__nan__nan_	Height_cm	0.426168	1.349245e-06
Bacteroidota__nan__nan__nan__nan__nan_	LMA	-0.425907	1.371670e-06
Actinobacteriota__Actinobacteria__Pseudonocardiales__Pseudonocardiaceae__Pseudonocardia__nan__	RootDry_g	-0.425575	1.400612e-06

Table 20: *ASV level*. Statistically significant differences between 212 ASVs (108 are unique) and plant traits based on Spearman's rank correlation coefficient with Holm–Bonferroni correction. The actual names of ASVs are not printed due to space, each ASV represented as *phylum_class_order_family_genus_species*. Part 2.
